# Inflammatory factors and risk of lung adenocarcinoma: a Mendelian randomization study mediated by blood metabolites

**DOI:** 10.3389/fendo.2024.1446863

**Published:** 2024-08-27

**Authors:** Zheng Ding, Juan Chen, Bohan Li, Xinyu Ji

**Affiliations:** ^1^ Department of Cardiac Surgery, The First Affiliated Hospital of China Medical University, Liaoning, Shenyang, China; ^2^ Department of Medical Oncology, The First Affiliated Hospital of China Medical University, Liaoning, Shenyang, China; ^3^ Department of Urinary Surgery, The First Affiliated Hospital of China Medical University, Liaoning, Shenyang, China; ^4^ Department of Thoracic Surgery, The First Affiliated Hospital of China Medical University, Liaoning, Shenyang, China

**Keywords:** lung adenocarcinoma, inflammatory factors, blood metabolites, causal inference, mediation analysis, mendelian randomization

## Abstract

**Background:**

Lung adenocarcinoma (LUAD) is the most common type of lung cancer, and its pathogenesis remains not fully elucidated. Inflammation and metabolic dysregulation are considered to play crucial roles in LUAD development, but their causal relationships and specific mechanisms remain unclear.

**Methods:**

This study employed a two-sample Mendelian randomization (MR) approach to systematically evaluate the causal associations between 91 circulating inflammatory factors, 1,400 serum metabolites, and LUAD. We utilized LUAD genome-wide association studies (GWAS) data from the FinnGen biobank and GWAS data of metabolites and inflammatory factors from the GWAS catalog to conduct two-sample MR analyses. For the identified key metabolites, we further used mediator MR to investigate their mediating effects in the influence of IL-17A on LUAD and explored potential mechanisms through protein-protein interaction and functional enrichment analyses.

**Results:**

The MR analyses revealed that IL-17A (OR 0.78, 95%CI 0.62-0.99) was negatively associated with LUAD, while 71 metabolites were significantly associated with LUAD. Among them, ferulic acid 4-sulfate may play a crucial mediating role in the suppression of LUAD by IL-17A (OR 0.87, 95%CI 0.78-0.97). IL-17A may exert its anti-LUAD effects through extensive interactions with genes related to ferulic acid 4-sulfate metabolism (such as SULT1A1, CYP1A1, etc.), inhibiting oxidative stress and inflammatory responses, as well as downstream tumor-related pathways of ferulic acid 4-sulfate (such as MAPK, NF-κB, etc.).

**Conclusion:**

This study discovered causal associations between IL-17A, multiple serum metabolites, and LUAD occurrence, revealing the key role of inflammatory and metabolic dysregulation in LUAD pathogenesis. Our findings provide new evidence-based medical support for specific inflammatory factors and metabolites as early predictive and risk assessment biomarkers for LUAD, offering important clues for subsequent mechanistic studies and precision medicine applications.

## 1 Introduction

Lung cancer remains the leading cause of cancer-related mortality worldwide. According to the latest global cancer statistics, there were 2.2 million new lung cancer cases and 1.8 million deaths in 2020 (1). Traditionally, lung cancer is divided into two main types: small cell lung cancer (SCLC) and non-small cell lung cancer (NSCLC). NSCLC accounts for approximately 85% of all lung cancers and mainly comprises two major subtypes: lung adenocarcinoma (LUAD) and lung squamous cell carcinoma (LUSC), with LUAD representing about 70% of NSCLC (2, 3). Although smoking is one of the primary risk factors for LUAD, the incidence of LUAD in non-smokers has been increasing in recent years (4), suggesting that there may be other important etiological factors and mechanisms beyond smoking that remain to be elucidated.

Inflammation has emerged as a critical hallmark of cancer, contributing to various stages of tumorigenesis, including initiation, promotion, malignant conversion, invasion, and metastasis. Chronic inflammation can create a tumor-promoting microenvironment by inducing angiogenesis, suppressing adaptive immunity, and promoting tumor cell survival and proliferation (5, 6). Numerous epidemiological studies have demonstrated that chronic inflammatory conditions, such as chronic obstructive pulmonary disease (COPD) and pulmonary fibrosis, are associated with an increased risk of lung cancer, particularly LUAD (7, 8). Moreover, elevated levels of circulating inflammatory markers, such as C-reactive protein (CRP), interleukin-6 (IL-6), and tumor necrosis factor-α (TNF-α), have been linked to poor prognosis and increased mortality in LUAD patients (9, 10). However, the causal role of specific inflammatory factors in LUAD development remains to be established.

Metabolic reprogramming, another hallmark of cancer, is characterized by altered energy metabolism and nutrient utilization to support the rapid growth and proliferation of cancer cells (11). The Warburg effect, a shift from oxidative phosphorylation to aerobic glycolysis, is a well-recognized metabolic phenotype in many cancer types, including LUAD (12, 13). In addition, cancer cells exhibit increased glutamine metabolism and lipid synthesis to meet their heightened demands for biosynthetic precursors and energy (14, 15). Interestingly, recent studies have revealed that metabolic alterations can modulate inflammatory responses and shape the tumor microenvironment (16). For example, the accumulation of lactate, a byproduct of aerobic glycolysis, can promote the polarization of tumor-associated macrophages towards an immunosuppressive M2 phenotype (17). Conversely, inflammatory signaling pathways, such as NF-κB and STAT3, can regulate the expression of metabolic enzymes and transporters, thereby reprogramming cellular metabolism (18, 19). Despite these intriguing findings, the complex interplay between inflammation and metabolism in the context of LUAD development remains poorly understood.

Mendelian randomization (MR) analysis has emerged as a powerful tool to assess the causal effects of exposures on outcomes by leveraging genetic variants as instrumental variables (20). By utilizing genetic variants that are randomly allocated at conception and not influenced by confounding factors or reverse causation, MR can provide more robust evidence for causal inference compared to traditional observational studies (21). In recent years, MR has been increasingly applied to investigate the causal roles of circulating biomarkers, such as inflammatory factors and metabolites, in various diseases, including cancer (22, 23). However, to date, no comprehensive MR study has systematically evaluated the causal effects of blood inflammatory factors and metabolites on LUAD risk.

In this study, we aim to conduct a two-sample MR analysis to systematically assess the causal relationships between blood inflammatory factors, metabolites, and LUAD risk using summary-level data from large-scale genome-wide association studies (GWAS). Importantly, we will employ a mediation MR design to investigate the potential mediating role of metabolites in the causal pathway from inflammatory factors to LUAD development. By integrating genetic, transcriptomic, and metabolomic data, we seek to unravel the complex molecular mechanisms underlying the inflammation-metabolism-LUAD axis and identify novel targets and strategies for the prevention, early detection, and treatment of LUAD. Our findings may provide new insights into the etiology of LUAD and contribute to the development of precision medicine approaches for this devastating disease.

## 2 Methods

### 2.1 Data sources

This study utilized three main data sources. First, we extracted data on 1,590 lung adenocarcinoma (LUAD) cases and 314,193 controls from the Finnish FinnGen biobank database (https://finngen.gitbook.io/documentation/data-download), with the dataset identifier C3_NSCLC_ADENO_EXALLC. Second, we leveraged GWAS results for 1,400 metabolites from the GWAS Catalog database (https://www.ebi.ac.uk/gwas/) (ID: GCST90199621 to GCST90201020) (24). Finally, we incorporated GWAS data for 91 circulating inflammatory factors from the Protein Research Department of Public Health and Primary Care at the University of Cambridge (https://www.phpc.cam.ac.uk/ceu/proteins and GWAS Catalog ID: GCST90274758 to GCST90274848) (25). Besides, the study participants in these three databases were all of European ancestry.

### 2.2 Selection of instrumental variables

MR is a powerful genetic epidemiology method that uses genetic variants as instrumental variables to infer causal relationships between exposures and outcomes. This approach leverages the random assortment of alleles during meiosis to mimic a randomized controlled trial, thereby minimizing confounding and reverse causation issues often encountered in observational studies.

To assess the causal relationships between blood metabolites, inflammatory factors, and LUAD, we employed a two-sample MR analysis approach. Two-sample MR allows us to use summary-level data from large-scale GWAS, increasing statistical power and enabling the investigation of multiple exposures simultaneously. MR analysis relies on three key assumptions: (1) the selected instrumental variables (genetic variants) must be strongly associated with blood metabolites or inflammatory factors; (2) the genetic variants must be independent of any potential confounders; and (3) the genetic variants must affect LUAD only through blood metabolites or inflammatory factors, and not through any other direct causal pathways (26). If assumption (1) is not met, MR analysis cannot be performed; if assumptions (2) and/or (3) are violated, it may lead to false-positive results.

To identify strong instrumental variables that satisfy assumption (1), we conducted an association analysis using summary data from GWAS. Considering that the number of single nucleotide polymorphisms (SNPs) obtained at a P-value threshold of 5×10^-8^ might be limited and insufficient to support further research, we relaxed the threshold to a P-value less than 1×10^-5^. Moreover, to ensure the independence of the selected instrumental variables, we performed linkage disequilibrium (LD) pruning on the variant loci using PLINK software, with a physical distance threshold of 10,000 kb and an R2 threshold of 0.001. By applying these stringent selection criteria, we ultimately obtained a set of strong instrumental variables that were closely associated with blood metabolites or inflammatory factors and were independent of each other, thereby minimizing the possibility of violating MR assumptions (2) and (3).

To further ensure the validity of the instrumental variables, we employed the F-statistic to assess their strength and excluded SNPs with an F-value less than 10. The F-statistic is calculated using the following formula: F = R^2^ × (N-2)/(1-R^2^), where R^2^ represents the proportion of variance in the exposure variable explained by the SNP, and N represents the sample size of the exposure data. The R^2^ value is calculated as: R^2^ = 2 × (1-MAF) × MAF × β^2^, where MAF is the minor allele frequency, and β is the effect size of the SNP on the exposure variable. By implementing these filtering criteria, we aimed to select informative and reliable instrumental variables for the MR analysis.

Furthermore, to explore the potential role of blood metabolites and inflammatory factors in the pathogenesis of LUAD, we also performed mediator MR analysis. Mediator MR analysis can help us assess the role of a mediating factor in the causal pathway between an exposure factor and an outcome. By comprehensively employing both two-sample MR and mediator MR methods, we can gain a more comprehensive understanding of the causal relationships between blood metabolites, inflammatory factors, and LUAD, as well as their potential mechanisms.

### 2.3 Statistical analysis

In this study, we employed multiple MR methods to comprehensively assess the causal relationships between blood inflammatory factors, metabolites, and LUAD, including Inverse-Variance Weighted (IVW), MR-Egger regression, Weighted Median (WM), Simple Mode, and Weighted Mode methods. Considering the robustness of the IVW method in causal inference (27), we selected it as the primary method for estimating causal effects.

To evaluate the robustness of the MR results, we performed several sensitivity analyses. First, we used the leave-one-out cross-validation method to assess the influence of individual SNPs on the overall causal effect estimates. Second, we employed MR-PRESSO (Pleiotropy Residual Sum and Outlier) and MR-TRYX (Treasure your exceptions) methods to detect and correct for pleiotropy bias. Additionally, we used Cochran’s Q test to assess the heterogeneity of causal effect estimates and the intercept term of MR-Egger regression to test for the presence of directional pleiotropy bias (28).

To further investigate the causal relationships between blood inflammatory factors, metabolites, and LUAD, we employed a two-step MR analysis to evaluate the mediating role of metabolites in the causal relationship between inflammatory factors and LUAD. In the first step, we conducted a two-sample MR analysis to estimate the total causal effect (α) of inflammatory factors on LUAD. We primarily used the IVW method and performed sensitivity analyses using MR-Egger regression and WM methods.

In the second step, we performed two separate two-sample MR analyses. First, we assessed the causal relationship between inflammatory factors and metabolites, obtaining the causal effect estimate β1. Then, we assessed the causal relationship between metabolites and LUAD, obtaining the causal effect estimate β2. In both analyses, we again used the IVW method as the primary method and MR-Egger regression and WM methods for sensitivity analyses. It is worth noting that when assessing the causal relationship between metabolites and LUAD, we needed to exclude genetic variants associated with inflammatory factors to avoid violating the key assumptions of MR. This can be achieved by conducting conditional analyses on inflammatory factors and metabolites, i.e., including inflammatory factors as covariates in the model when assessing the causal relationship between metabolites and LUAD.

Finally, we calculated the indirect causal effect estimate β3 as the product of β1 and β2. We then divided β3 by the total causal effect estimate α to obtain the proportion of the mediating effect. To assess the significance of the mediating effect, we used the Bootstrap method to calculate the confidence intervals and p-values for the indirect effect.

All MR analyses were performed using the R language (version 4.4.1) and related R packages such as “TwoSampleMR” and “MRInstruments.”

## 3 Results

**Figure 1:**
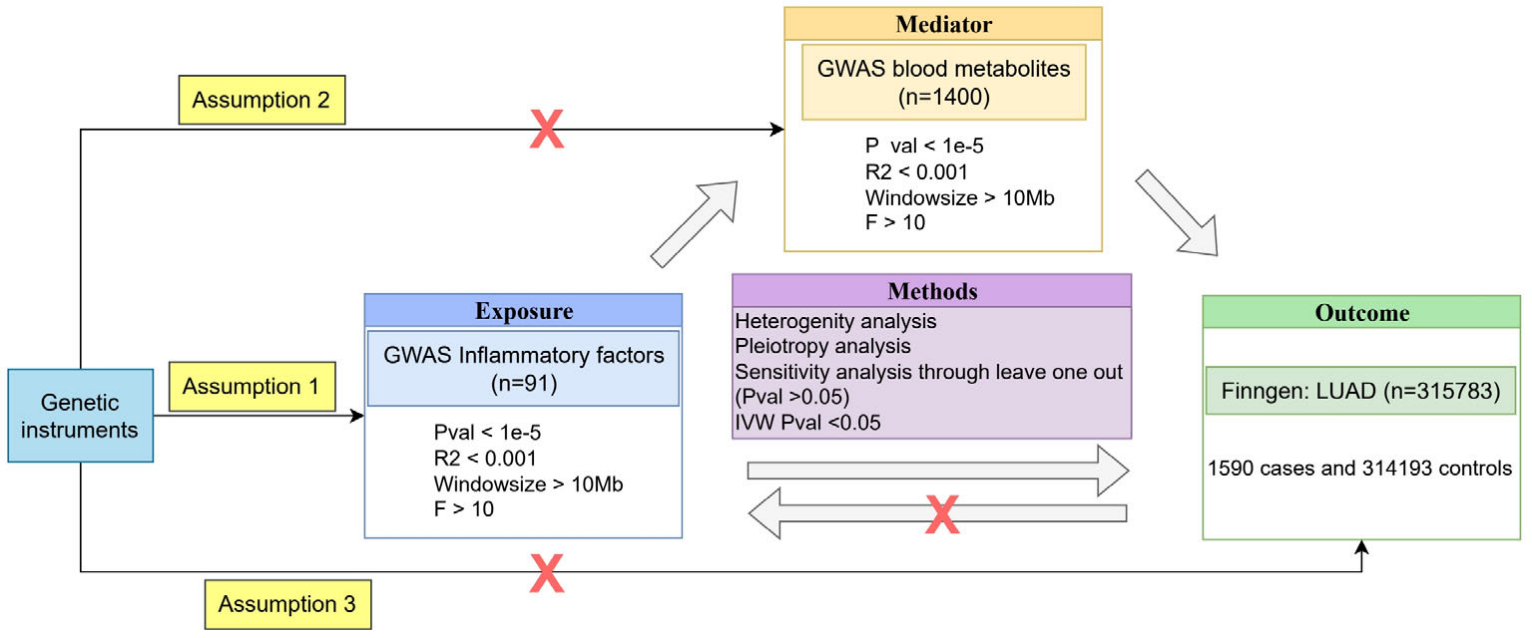
MR Analysis Process Flowchart.

All MR analysis process are shown in **Figure 1**.

### 3.1 Assessment of the causal relationship between circulating inflammatory factors and LUAD

**Figure 2:**
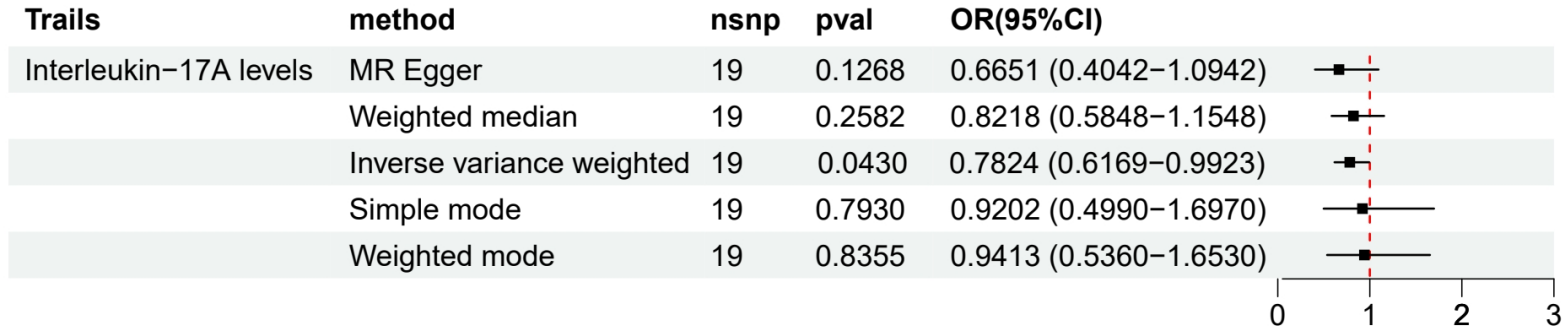
Forest map of MR results of IL-7A and LUAD.

We used a two-sample MR approach to evaluate the causal relationship between 91 circulating inflammatory factors and LUAD. Preliminary results (**Figure 2**) showed that among all the analyzed inflammatory factors, only Interleukin-17A (IL-17A) had a statistically significant difference and was further identified as a protective factor for LUAD based on the OR value (OR 95% CI = 0.7824 (0.6169−0.9923), p = 0.0430). This result suggests that an increase in IL-17A levels may have a causal relationship with a reduced risk of LUAD (β = -0.2454). However, no significant causal relationships were observed between other inflammatory factors and LUAD.

To assess the robustness of the results, we performed multiple sensitivity analyses (**Supplementary Material S1**). MR-Egger regression and WM methods yielded results consistent with the IVW method, further supporting the protective causal relationship between IL-17A and LUAD. Moreover, Cochran’s Q test did not detect significant heterogeneity (p > 0.05), and MR-PRESSO and MR-TRYX analyses did not identify substantial pleiotropy bias.

To investigate whether LUAD conversely affects circulating inflammatory factor levels, we performed a reverse MR analysis. The results showed that LUAD had no significant causal effect on any of the 91 circulating inflammatory factors. This finding excludes the possibility that LUAD itself leads to changes in IL-17A levels, further supporting the unidirectional causal effect of IL-17A on LUAD.

### 3.2 Assessment of the causal relationship between metabolites and LUAD

We used a two-sample MR approach to evaluate the causal relationship between various metabolites and LUAD. Based on the consistency of different MR methods, we classified the results into three evidence levels: meeting inclusion criteria (only the IVW method showed statistical significance), strong evidence (2-3 methods showed statistical significance), and very strong evidence (≥4 methods showed statistical significance).

**Figure 3:**
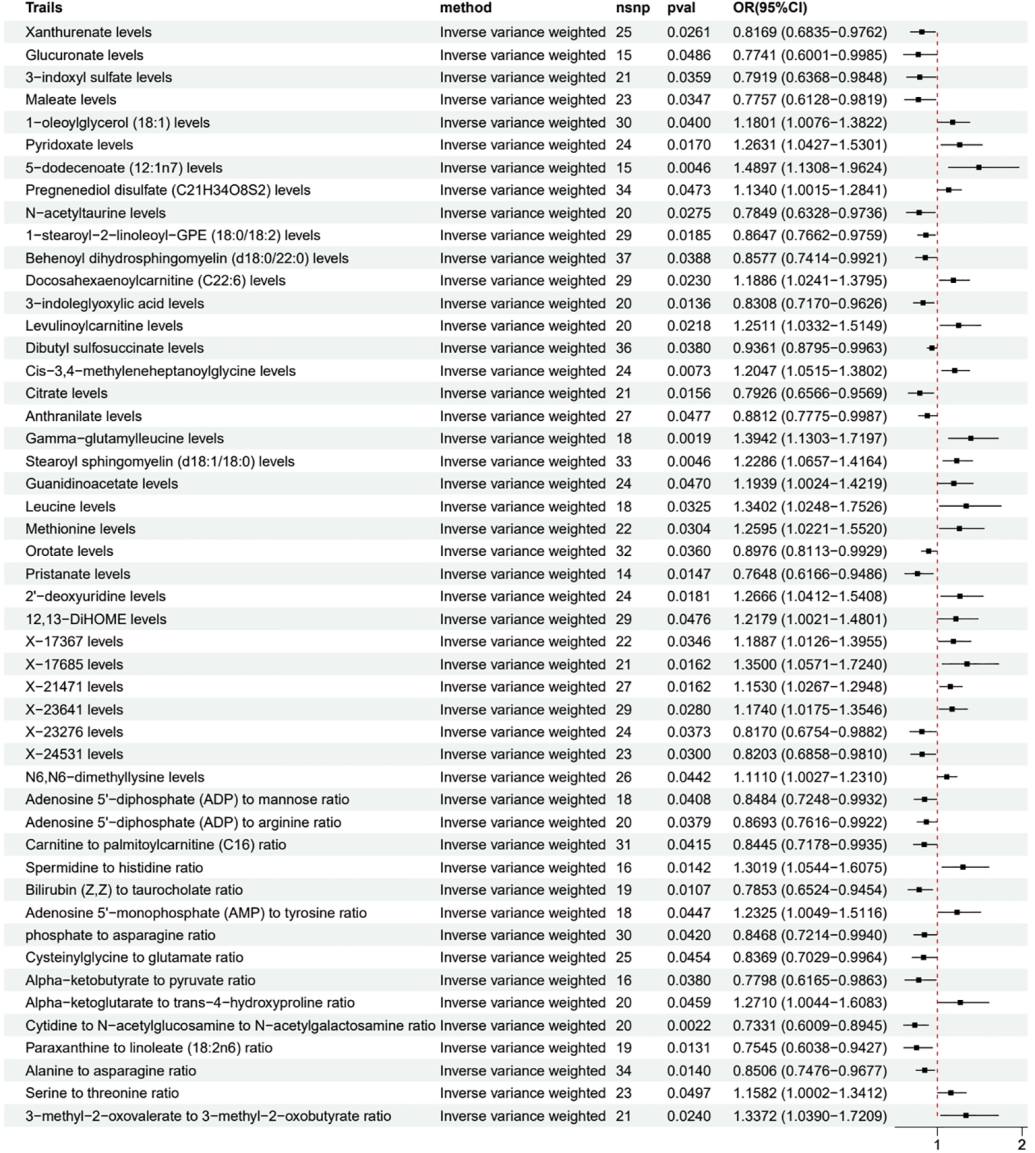
Forest plot of MR analysis for metabolites meeting inclusion criteria and LUAD risk.

Among the metabolites meeting the inclusion criteria (**Figure 3**), we found that several metabolites may be associated with a reduced risk of LUAD, including Xanthurenate (OR 0.82, 95% CI 0.68-0.98), Glucuronate (OR 0.77, 95% CI 0.60-1.00), and 3-indoxyl sulfate (OR 0.79, 95% CI 0.64-0.98). Simultaneously, some metabolites such as 1-oleoylglycerol (18:1) (OR 1.18, 95% CI 1.01-1.38), Pyridoxate (OR 1.26, 95% CI 1.04-1.53), and 5-dodecenoate (12:1n7) (OR 1.49, 95% CI 1.13-1.96) may be associated with an increased risk of LUAD.

**Figure 4:**
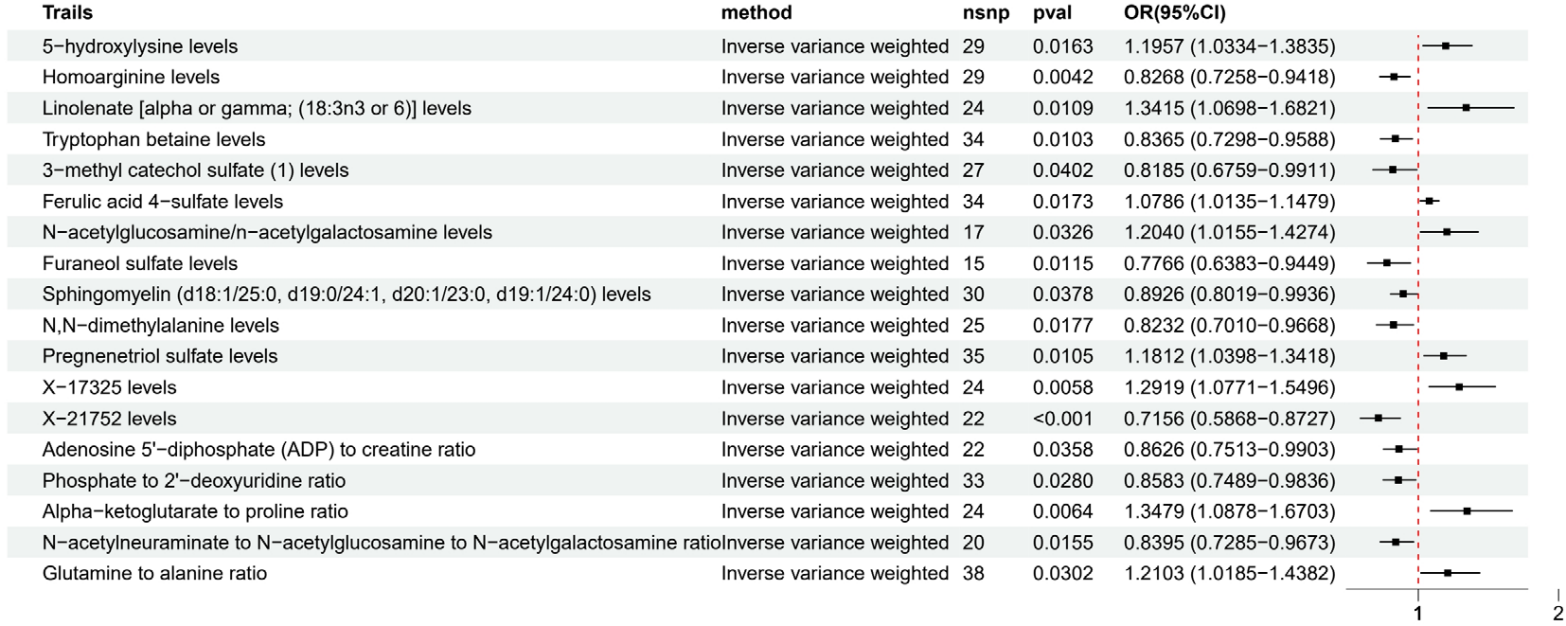
Forest plot of MR analysis for metabolites with strong evidence and LUAD risk.

Among the metabolites with strong evidence (**Figure 4**), 5-hydroxylysine (OR 1.20, 95% CI 1.03-1.38), Linolenate [alpha or gamma; (18:3n3 or 6)] (OR 1.34, 95% CI 1.07-1.68), and Pregnenetriol sulfate (OR 1.18, 95% CI 1.04-1.34) were associated with an increased risk of LUAD, while Homoarginine (OR 0.83, 95% CI 0.73-0.94), Tryptophan betaine (OR 0.84, 95% CI 0.73-0.96), and Furaneol sulfate (OR 0.78, 95% CI 0.64-0.94) may have a protective effect.

**Figure 5:**
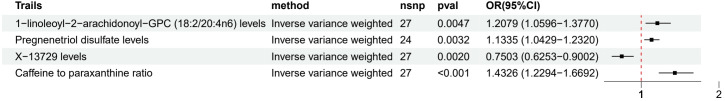
Forest plot of MR analysis for metabolites with very strong evidence and LUAD risk.

Metabolites with very strong evidence (**Figure 5**) included 1-linoleoyl-2-arachidonoyl-GPC (18:2/20:4n6) (OR 1.21, 95% CI 1.06-1.38), Pregnenetriol disulfate (OR 1.13, 95% CI 1.04-1.23), X-13729 (OR 0.75, 95% CI 0.63-0.90), and Caffeine to paraxanthine ratio (OR 1.43, 95% CI 1.23-1.67), suggesting that the causal relationships between these metabolites and LUAD are more reliable.

Additionally, we found associations between some metabolite ratios and LUAD risk. An increased Spermidine to histidine ratio (OR 1.30, 95% CI 1.05-1.61) and a decreased Bilirubin (Z,Z) to taurocholate ratio (OR 0.79, 95% CI 0.65-0.95) were associated with an increased risk of LUAD, while a decreased Cytidine to N-acetylglucosamine to N-acetylgalactosamine ratio (OR 0.73, 95% CI 0.60-0.89) and a decreased Alanine to asparagine ratio (OR 0.85, 95% CI 0.75-0.97) may have a protective effect. The leave-one-out, heterogeneity, and pleiotropy analyses for each metabolite and LUAD are presented in **Supplementary Material S2**.

### 3.3 Assessment of the causal relationship between circulating inflammatory factors and metabolites

To explore the potential mediating role of metabolites between inflammatory factors and LUAD, we further evaluated the causal relationship between IL-17A (the protective inflammatory factor for LUAD identified in the previous section) and related metabolites. We used a two-sample MR approach, with IL-17A as the exposure and metabolites as the outcome, to test the causal relationship between the two.

**Figure 6:**
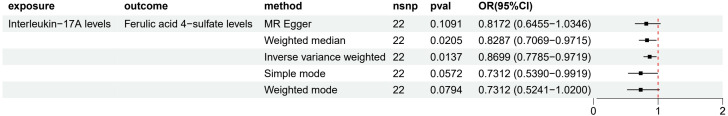
Forest plot of MR analysis for the causal effect of IL-17A on metabolites.

The results from the IVW method (**Figure 6**) showed a significant negative causal relationship between IL-17A and Ferulic acid 4-sulfate levels (OR 0.87, 95% CI 0.78-0.97, p = 0.0137), suggesting that an increase in IL-17A levels may lead to a decrease in Ferulic acid 4-sulfate levels (β1 = - 0.1394). Considering the previous results showing a positive correlation between Ferulic acid 4-sulfate levels and LUAD risk (OR 1.08, 95% CI 1.01-1.15, p = 0.0173), this finding suggests that Ferulic acid 4-sulfate may play a mediating role in the protective effect of IL-17A on LUAD. In other words, IL-17A may reduce the risk of LUAD by lowering Ferulic acid 4-sulfate levels. For other analysis results, such as heterogeneity, pleiotropy and sensitivity analysis, the **Supplementary Material S3** files showed the details.

However, we did not find significant causal relationships between IL-17A and other metabolites (p > 0.05). Moreover, for other circulating inflammatory factors, we did not observe significant causal relationships between them and metabolites. This suggests that metabolites may only play an important mediating role in the pathway of IL-17A, while their role in the pathogenic mechanisms of other inflammatory factors in LUAD is limited.

### 3.4 Functional inference of IL-17A on Ferulic acid 4-sulfate-related gene metabolic networks

**Figure 7:**
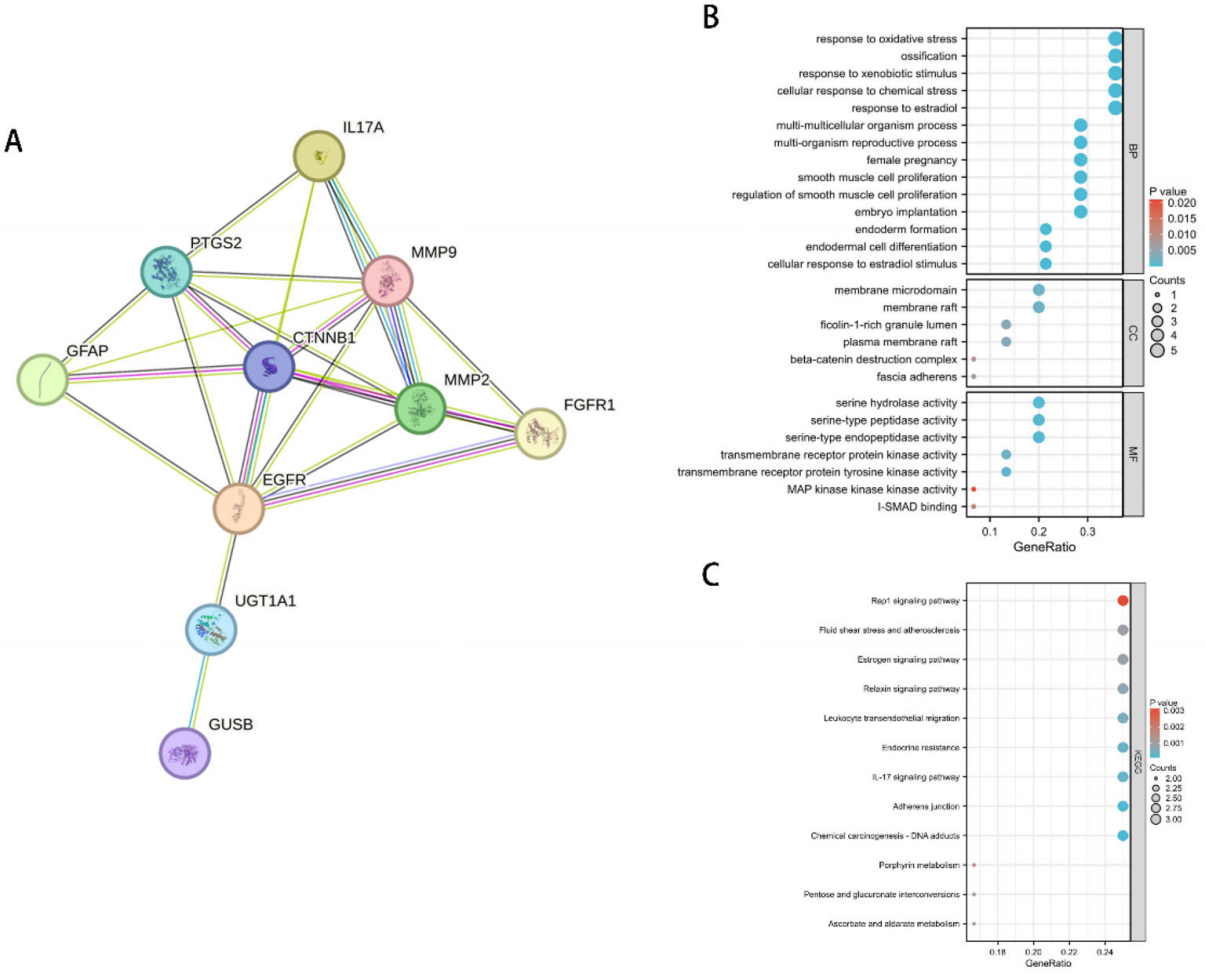
Regulatory network and functional enrichment analysis of IL-17A on Ferulic acid 4-sulfate-related genes. **(A)** PPI network of IL-17A and Ferulic acid 4-sulfate-related genes. **(B)** GO functional enrichment bubble plot of Ferulic acid 4-sulfate-related genes. **(C)** KEGG pathway enrichment bar plot of Ferulic acid 4-sulfate-related genes.

The previous MR analysis results suggest that IL-17A may inhibit the occurrence and development of LUAD by reducing Ferulic acid 4-sulfate levels. To further explore the underlying molecular mechanisms, we used the GeneCards database (https://www.genecards.org/) to query the genes related to Ferulic acid 4-sulfate and constructed a protein-protein interaction (PPI) network with the IL-17A gene (**Figure 7A**). The results showed that the genes related to Ferulic acid 4-sulfate have extensive interactions with IL-17A within the cell, suggesting that IL-17A may regulate the expression or activity of these genes, thereby reducing Ferulic acid 4-sulfate levels.

To reveal the functional enrichment of Ferulic acid 4-sulfate-related genes regulated by IL-17A, we performed Gene Ontology (GO) and Kyoto Encyclopedia of Genes and Genomes (KEGG) pathway enrichment analyses on the genes in the PPI network. The results of the GO analysis (**Figure 7B**) showed that these genes were mainly enriched in biological processes such as response to oxidative stress, response to xenobiotic stimulus, and response to estradiol, as well as cellular components such as extracellular matrix and adherens junction. This suggests that IL-17A may inhibit the occurrence and development of LUAD by suppressing the oxidative stress and inflammatory responses involving Ferulic acid 4-sulfate-related genes and by affecting the extracellular matrix and cell junctions, thereby reshaping the tumor microenvironment.

The results of the KEGG pathway enrichment analysis (**Figure 7C**) showed that Ferulic acid 4-sulfate-related genes were mainly enriched in pathways such as Rap1 signaling pathway, Fluid shear stress and atherosclerosis, Estrogen signaling pathway, and Relaxin signaling pathway. This suggests that IL-17A may exert its anti-LUAD effect by inhibiting these signaling pathways, thereby reducing Ferulic acid 4-sulfate levels.

## 4 Discussion

This study comprehensively evaluated the causal relationships among circulating inflammatory factors, serum metabolites, and lung adenocarcinoma (LUAD) using Mendelian randomization (MR) analysis. Among 91 circulating inflammatory factors and 1,400 serum metabolites, we identified one inflammatory factor (IL-17A) and 71 serum metabolites that exhibited potential causal associations with LUAD. Specifically, we first employed a two-sample MR approach to assess the causal relationship between circulating inflammatory factors and LUAD. The results revealed that IL-17A was inversely associated with LUAD risk (OR 0.78, 95% CI 0.62-0.99), suggesting a protective role of IL-17A in LUAD pathogenesis. Subsequently, we applied the same method to evaluate the causal relationship between serum metabolites and LUAD. Based on the consistency of results from different MR methods, we classified the metabolites into three evidence levels. Among the metabolites meeting the inclusion criteria, several metabolites such as xanthurenate, glucuronate, and 3-indoxyl sulfate were found to be potentially associated with a reduced risk of LUAD, while 1-oleoylglycerol (18:1), pyridoxate, and 5-dodecenoate (12:1n7) were potentially associated with an increased risk of LUAD. In metabolites with higher evidence levels, we further identified multiple metabolites associated with an increased (e.g., 5-hydroxylysine and linolenate) or decreased (e.g., homoarginine and tryptophan betaine) risk of LUAD. Additionally, changes in the ratios of certain metabolites were also related to LUAD risk. To explore the potential mechanisms underlying the anti-LUAD effects of IL-17A, we further assessed the causal relationship between IL-17A and metabolites using the mediation Mendelian randomization approach. The results showed that IL-17A significantly reduced the levels of ferulic acid 4-sulfate (OR 0.87, 95% CI 0.78-0.97), which was positively correlated with LUAD risk. This finding suggests that ferulic acid 4-sulfate may play a crucial mediating role in the suppression of LUAD by IL-17A. Subsequent protein-protein interaction network and functional enrichment analyses further revealed that IL-17A might exert its anti-LUAD effects by inhibiting oxidative stress, inflammatory responses, and tumor-related signaling pathways involving ferulic acid 4-sulfate-related genes.

Among the 91 circulating inflammatory factors analyzed, only IL-17A exhibited a significant negative correlation with LUAD, while no significant causal associations were found between other inflammatory factors and LUAD. This result suggests that the protective effect of IL-17A on LUAD may be specific and independent of changes in other inflammatory factors. Moreover, previous studies on the relationship between IL-17A and lung cancer have yielded contradictory results. Some studies have reported the overexpression of IL-17A in lung cancer tissues and its promotion of lung cancer progression and metastasis (29, 30). However, other studies have reported that IL-17A can inhibit lung cancer growth and angiogenesis (31, 32). These discrepancies may be attributed to factors such as study design, sample size, and tumor heterogeneity. Unlike previous studies, we utilized the MR analysis approach based on genome-wide association study data to evaluate the causal relationship between IL-17A and LUAD at the population level, avoiding the influence of potential confounding factors and reverse causality. In comparison, our results provide more reliable evidence supporting the protective role of IL-17A against LUAD. This finding not only highlights the unique role of IL-17A in LUAD pathogenesis but also provides a solid theoretical basis for targeting IL-17A as a specific target for LUAD prevention and treatment.

In recent years, metabolomics research has been widely applied in the field of lung cancer, with numerous studies revealing that metabolite profiles in serum, urine, and tissue samples are closely associated with the occurrence, progression, and prognosis of lung cancer (33–35). However, due to the case-control design employed in most previous studies, it is challenging to distinguish whether metabolite changes are the cause or consequence of lung cancer. Furthermore, factors such as small sample sizes and differences in metabolomics platforms have limited the reproducibility and generalizability of previous research findings.

In contrast to existing studies, the present study employed a two-sample Mendelian randomization (MR) approach to systematically evaluate the causal relationship between 1,400 serum metabolites and lung adenocarcinoma (LUAD). The results demonstrated that changes in the levels of multiple metabolites were significantly associated with LUAD risk. Among the metabolites with strong and very strong evidence levels, various categories were identified, including amino acids and their derivatives, lipids, nucleotides, and organic acids, involving multiple metabolic pathways. In terms of amino acid metabolism, we discovered that several amino acid derivatives, such as 5-hydroxylysine, homoarginine, tryptophan betaine, and N,N-dimethylalanine, were significantly associated with LUAD risk (36). Notably, 5-hydroxylysine is a derivative of hydroxylysine and is involved in collagen synthesis and cross-linking (37), while homoarginine is a homolog of arginine and has a regulatory effect on nitric oxide synthase activity (38). These findings suggest that amino acid metabolic disturbances may contribute to the occurrence and development of LUAD through multiple processes, such as post-translational protein modifications and cell signaling transduction. Regarding lipid metabolism, we found that several lipid metabolites, including linolenate, 1-linoleoyl-2-arachidonoyl-GPC (18:2/20:4n6), and sphingomyelin (d18:1/25:0, d19:0/24:1, d20:1/23:0, d19:1/24:0), were significantly associated with LUAD risk (39–41). Linolenate is an ω-3 polyunsaturated fatty acid with various biological activities, such as anti-inflammatory and anti-tumor effects (40), while sphingomyelin is an essential membrane lipid involved in multiple biological processes, including cell apoptosis and proliferation (42). These results suggest that lipid metabolic dysregulation may play a crucial role in the pathological process of LUAD by influencing the composition and function of cell membranes and the activity of related signaling pathways. In terms of nucleotide metabolism, we discovered that pregnenolone steroid metabolites, such as pregnenetriol disulfate and pregnenetriol sulfate, were significantly associated with LUAD risk (43). These metabolites are precursors or metabolic products of steroid hormones, indicating that alterations in steroid hormone metabolism may be closely related to the occurrence and development of LUAD. Additionally, we found that several sulfur-containing metabolites, such as ferulic acid 4-sulfate and 3-methyl catechol sulfate, were significantly associated with LUAD risk, revealing the important role of phase II metabolic reactions, such as sulfation, in the metabolomic changes associated with LUAD (44, 45).

Apart from changes in individual metabolite levels, we also discovered that alterations in multiple metabolite ratios were significantly associated with LUAD risk. For instance, elevated ratios of spermidine/histidine, alpha-ketoglutarate/trans-4-hydroxyproline, and serine/threonine were significantly associated with an increased risk of LUAD (46–49), while elevated ratios of adenosine 5’-diphosphate (ADP)/mannose, phosphate/asparagine, and bilirubin (Z,Z)/taurocholate were significantly associated with a decreased risk of LUAD (50–52). These findings suggest that the relative balance between metabolites may play a critical regulatory role in the occurrence and development of LUAD, and changes in metabolite ratios may reflect disturbances in specific metabolic pathways.

Further mediator analysis suggested that IL-17A may indirectly suppress the occurrence of LUAD by reducing the levels of ferulic acid 4-sulfate. This finding not only reveals the protective role of IL-17A in LUAD pathogenesis but also indicates that ferulic acid 4-sulfate may be an important downstream metabolite through which IL-17A exerts its anti-tumor effects. Subsequent bioinformatics analysis revealed extensive protein-protein interactions and co-expression relationships between IL-17A and ferulic acid 4-sulfate metabolism-related genes, such as SULT1A1 and CYP1A1. Furthermore, ferulic acid 4-sulfate metabolism-related genes were significantly enriched in key biological processes involved in tumor occurrence and development, such as oxidative stress and inflammatory responses. Among these genes, SULT1A1, CYP1A1, and others encode enzymes involved in the metabolism of endogenous and exogenous compounds, and their increased activity can lead to DNA damage, cell apoptosis, and other pro-tumorigenic effects. As an important anti-inflammatory factor, IL-17A may exert its anti-tumor effects by inhibiting the expression of these genes, thereby reducing the levels of oxidative stress and inflammatory responses in the body. This hypothesis is consistent with previous research findings on the role of IL-17A in tumor immunity (53), but its specific molecular mechanisms still require further experimental validation. In addition to directly regulating ferulic acid 4-sulfate metabolism-related genes, IL-17A may also exert anti-LUAD effects by inhibiting downstream signaling pathways of ferulic acid 4-sulfate. Previous studies have shown that ferulic acid and its derivatives can promote tumor cell proliferation, invasion, and metastasis by activating signaling pathways such as MAPK and NF-κB (54), while IL-17A can inhibit the activity of these tumor-related signaling pathways (53). Therefore, IL-17A may ultimately exert its anti-LUAD effects by reducing the levels of ferulic acid 4-sulfate and subsequently inhibiting its downstream pro-tumorigenic signaling pathways. This hypothesis needs to be confirmed through cellular and animal experiments in future studies.

Although MR analysis can effectively assess the causal relationship between metabolites and LUAD, there are still some limitations and potential biases. First, the effectiveness of MR analysis depends on the rationality of the selected instrumental variables (55). Although we used multiple independent GWAS datasets and conducted comprehensive sensitivity analyses, we cannot completely exclude the potential horizontal pleiotropy and weak instrument bias between genetic instrumental variables and LUAD. Second, MR analysis has difficulty exploring the nonlinear relationships and dynamic changes between metabolites and LUAD. Moreover, our study mainly focused on serum metabolites, while metabolite changes in other tissues and cell types and their relationship with LUAD remain to be further explored. Additionally, the metabolomics data used in this study were derived from European populations, with a relatively small sample size in the experimental group compared to the control group, and the coverage of the metabolite spectrum was limited. This may have affected our ability to identify new metabolite biomarkers for LUAD. Furthermore, differences between metabolomics platforms may also impact the reproducibility and generalizability of the results. Future studies need to conduct cross-validation using multiple metabolomics platforms in larger sample sizes and different populations to obtain more robust and comprehensive results. Integrating metabolomics with other omics data (such as proteomics and transcriptomics) and constructing multi-omics integration models will also help better understand the role of metabolite changes in the pathogenesis of LUAD. Finally, it is worth noting that although we preliminarily explored the molecular mechanisms of the interaction between IL-17A and ferulic acid 4-sulfate through bioinformatics analysis, these results are mainly based on published literature and database information and still require experimental validation in cellular and animal models. Future studies can use techniques such as gene editing and RNA interference to manipulate the expression of IL-17A and ferulic acid 4-sulfate-related genes in LUAD cell lines and mouse models, observe their effects on tumor occurrence and development, and explore the specific molecular mechanisms of their interaction. Integrating multi-omics data and constructing the regulatory network of IL-17A and ferulic acid 4-sulfate in LUAD will help to more comprehensively understand their functions and mechanisms of action.

## 5 Conclusion

In summary, this study employed two-sample MR and bioinformatics analysis to systematically evaluate the causal relationship between inflammatory factors, serum metabolites and LUAD, revealing that inflammatory and metabolic disorders play a key role in LUAD pathogenesis. The study also identified the important roles of IL-17A and ferulic acid 4-sulfate and their interaction in the occurrence and development of LUAD, as well as several previously rarely reported LUAD-related metabolites and metabolic pathways. Our research findings provide new evidence-based medical evidence for the application of specific metabolites as early prediction and risk assessment biomarkers for LUAD, and offer important clues for subsequent mechanistic studies, drug development, and precision medicine applications.

Future research can build upon these findings to further explore the molecular mechanisms of the relevant metabolites in LUAD occurrence and development, develop early diagnosis and risk prediction strategies for lung cancer based on inflammatory and metabolomic interactions, and integrate them with proteomics, transcriptomics, and other multi-omics data to construct molecular network regulatory models for LUAD. This will help to more comprehensively understand the molecular pathological processes of LUAD and promote the application of precision medicine in the field of lung cancer prevention and treatment. Additionally, the relationship between IL-17A and ferulic acid 4-sulfate levels and LUAD risk can be evaluated to develop lung cancer risk prediction models and targeted intervention strategies based on these two factors. By comprehensively utilizing these strategies, it is hoped that the prevention, diagnosis, and treatment of lung cancer can be significantly improved, bringing more benefits to lung cancer patients.

## Data Availability

The original contributions presented in the study are included in the article/**Supplementary Material**. Further inquiries can be directed to the corresponding authors.
