# Supplementary figures and images for: Inflammatory factors and risk of lung adenocarcinoma: a Mendelian randomization study mediated by blood metabolites
Source: Front Endocrinol (Lausanne). 2024 Aug 27;15:1446863. doi: 10.3389/fendo.2024.1446863 (PMC11384989; doi:10.3389/fendo.2024.1446863)

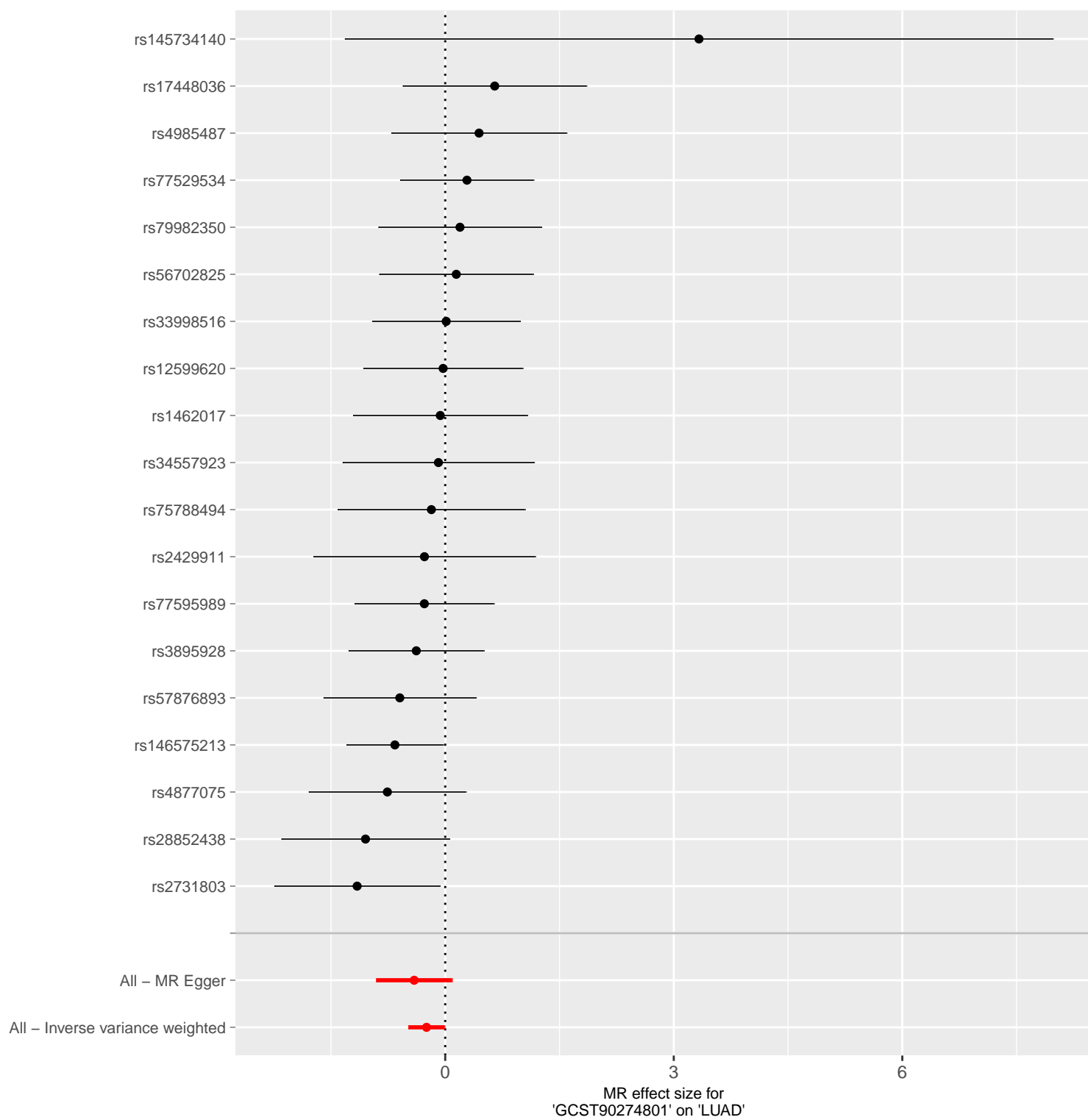

Supplement: Supplementary file 1 [file DataSheet1.zip › supplementary files/S1/GCST90274801/forest.pdf]

# MR Method

- Inverse variance weighted
- MR Egger

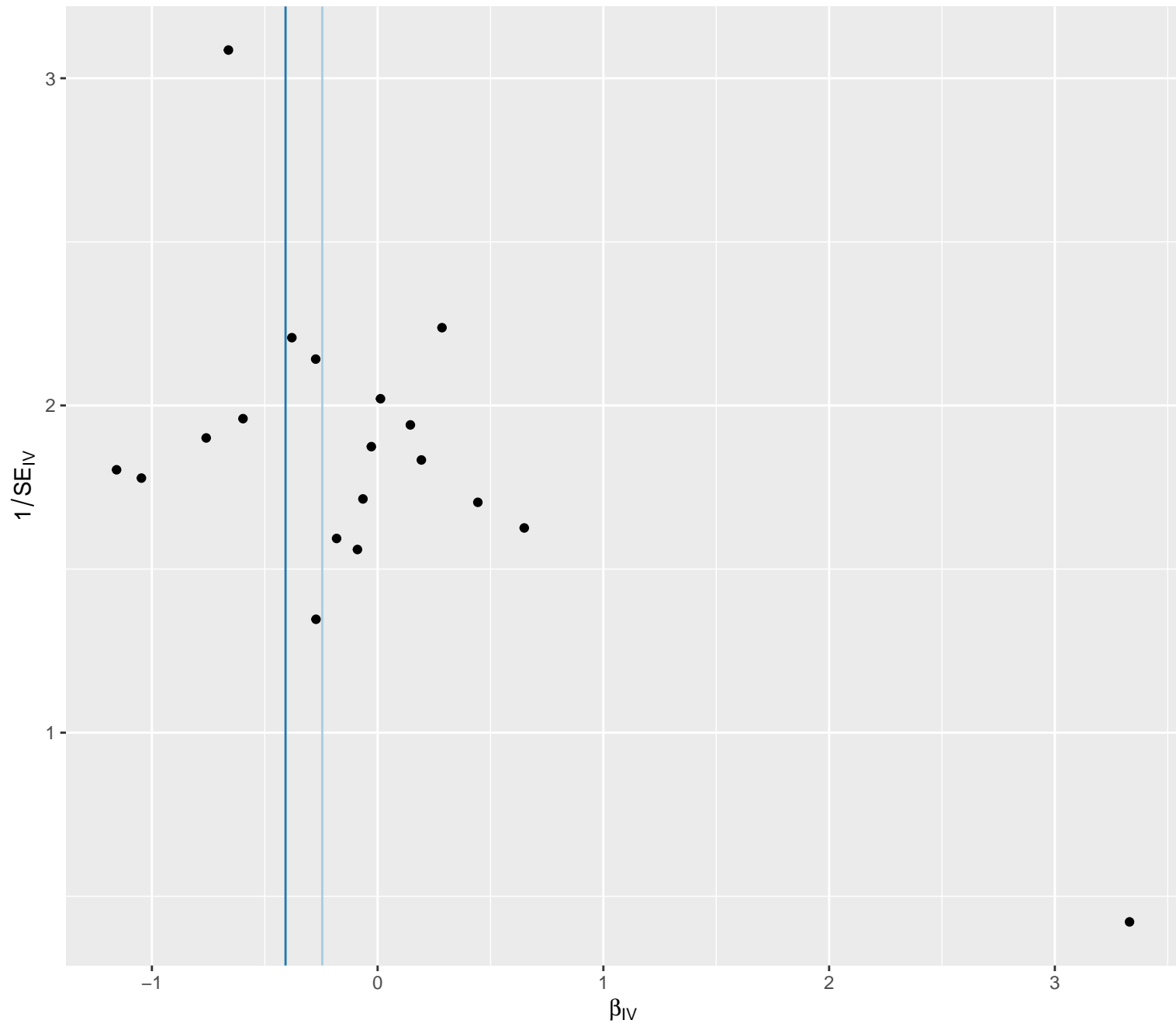

Supplement: Supplementary file 1 [file DataSheet1.zip › supplementary files/S1/GCST90274801/funnelplot.pdf]

# MR Test

- Inverse variance weighted
- MR Egger
- Simple mode
- Weighted median
- Weighted mode

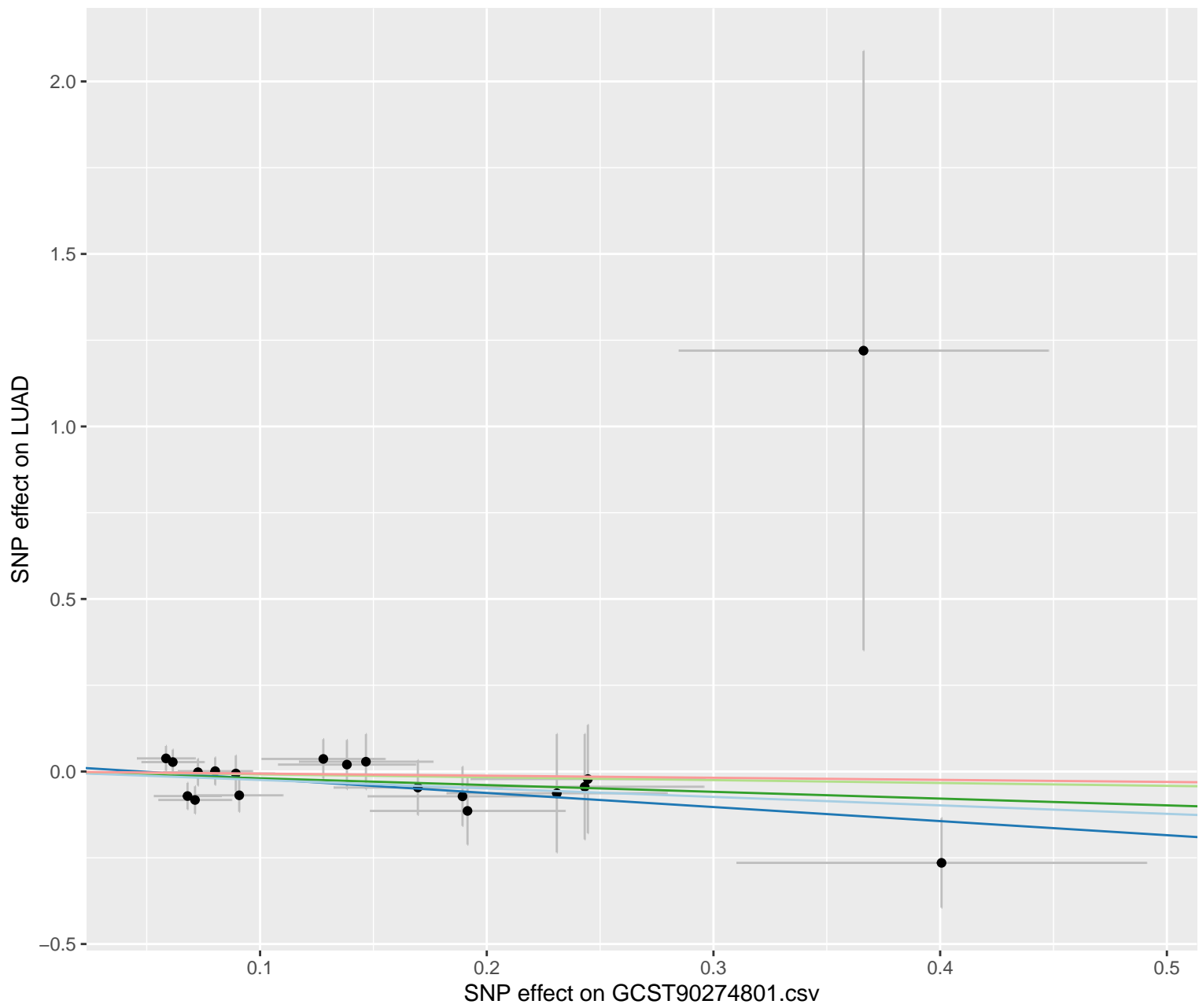

Supplement: Supplementary file 1 [file DataSheet1.zip › supplementary files/S1/GCST90274801/scatter.pdf]

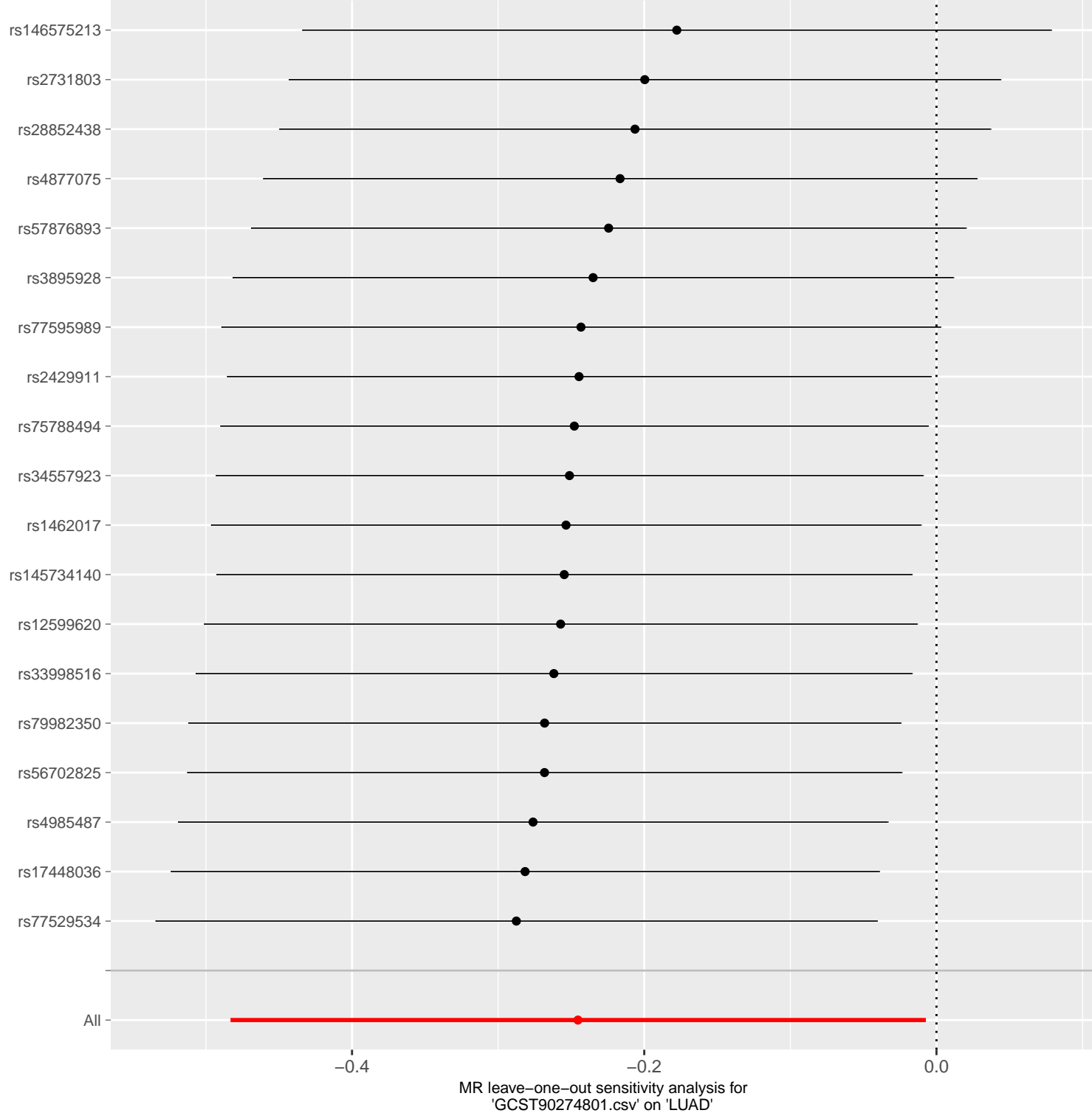

Supplement: Supplementary file 1 [file DataSheet1.zip › supplementary files/S1/GCST90274801/sensitivity-analysis.pdf]

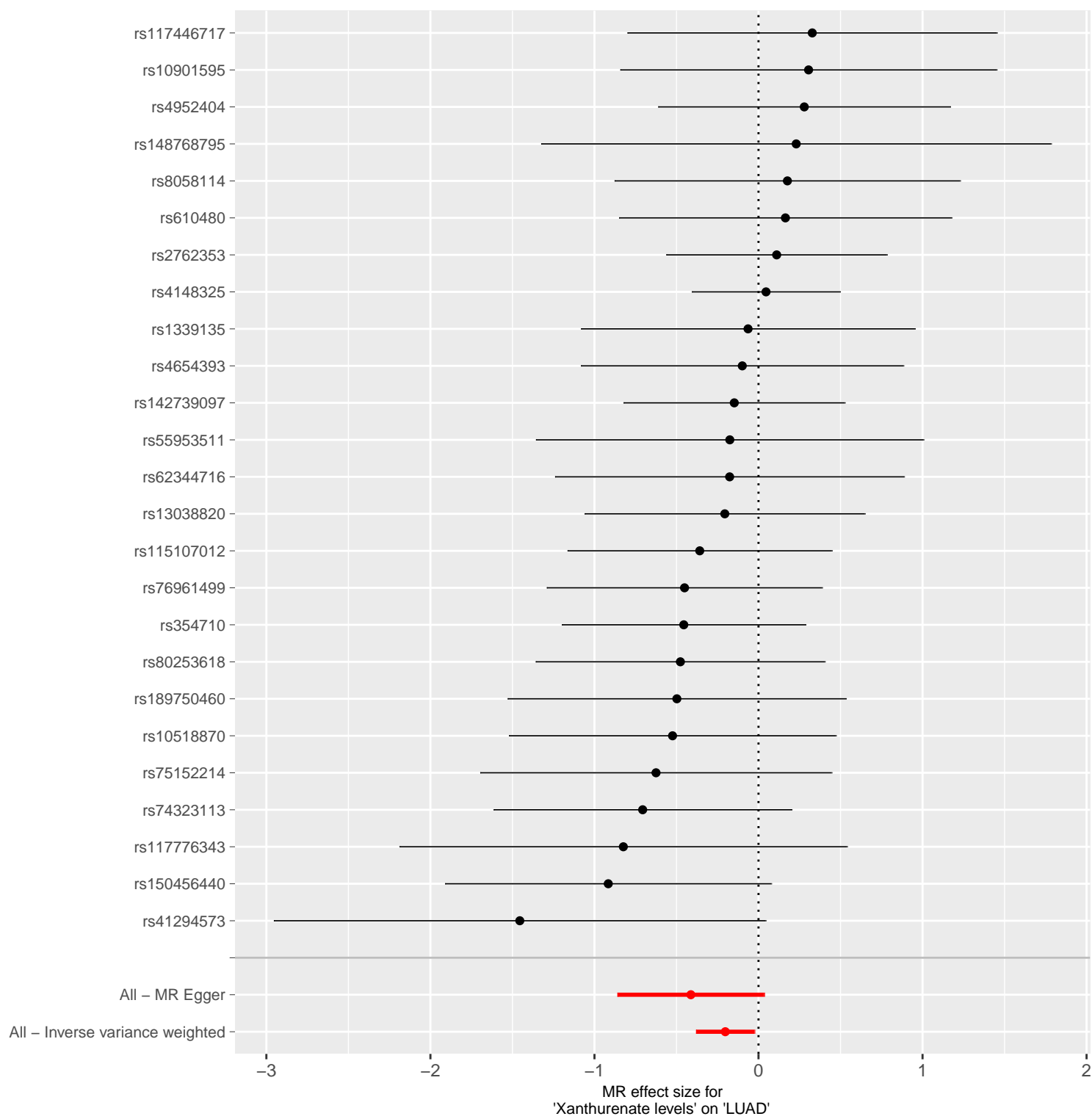

Supplement: Supplementary file 1 [file DataSheet1.zip › supplementary files/S2/GCST90199626/forest.pdf]

# MR Method

- Inverse variance weighted
- MR Egger

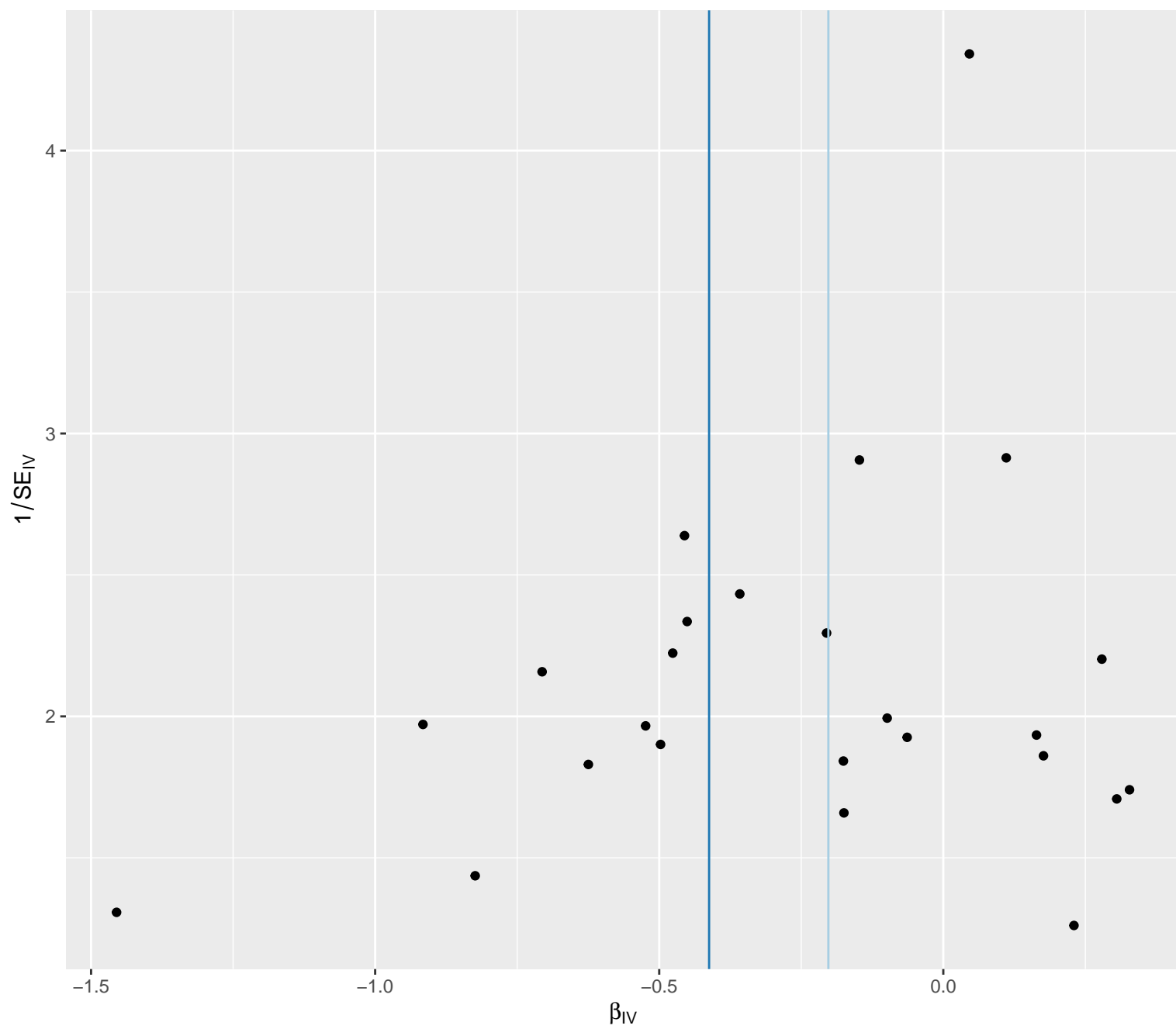

Supplement: Supplementary file 1 [file DataSheet1.zip › supplementary files/S2/GCST90199626/funnelplot.pdf]

# MR Test

- Inverse variance weighted
- MR Egger
- Simple mode
- Weighted median
- Weighted mode

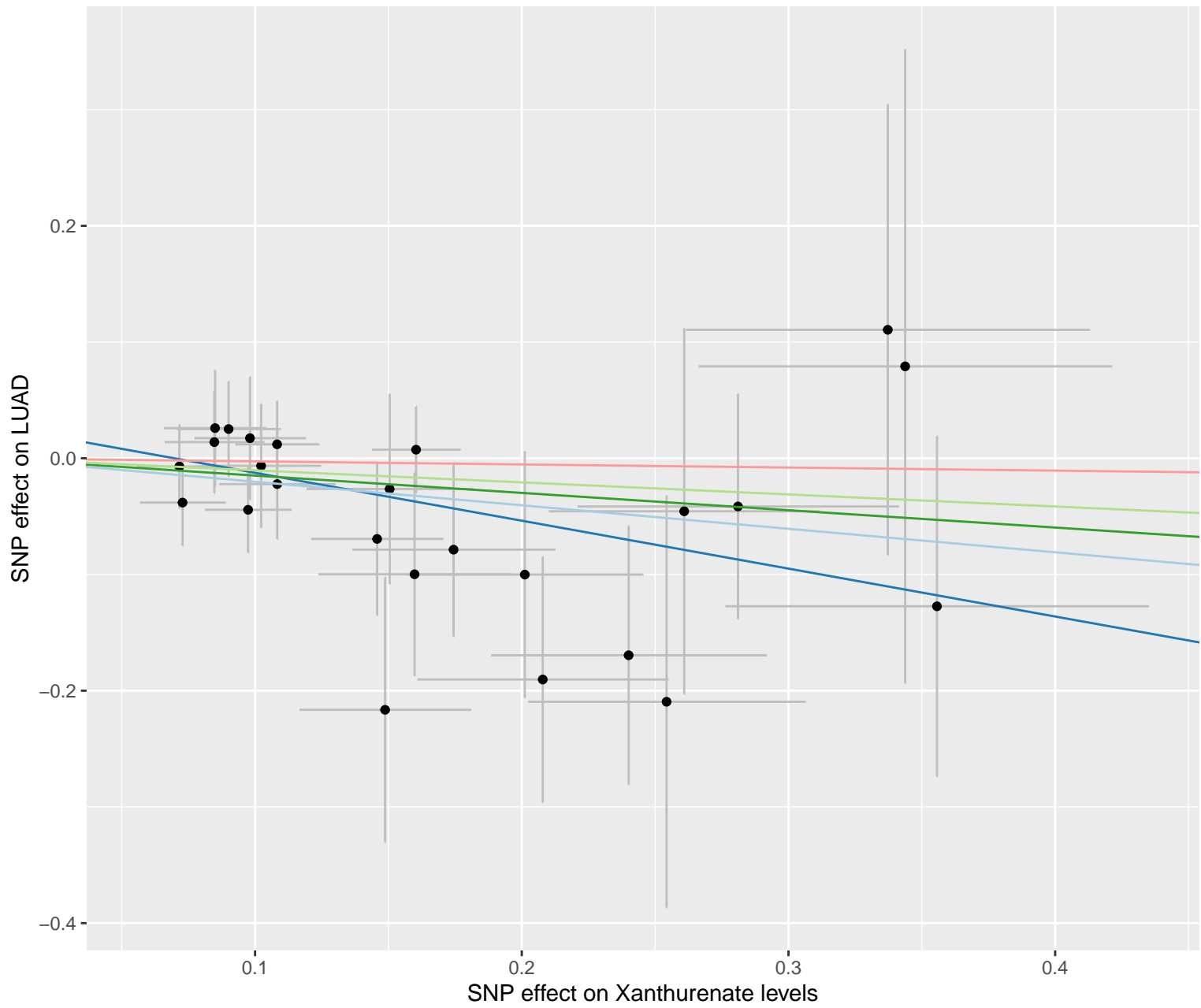

Supplement: Supplementary file 1 [file DataSheet1.zip › supplementary files/S2/GCST90199626/scatter.pdf]

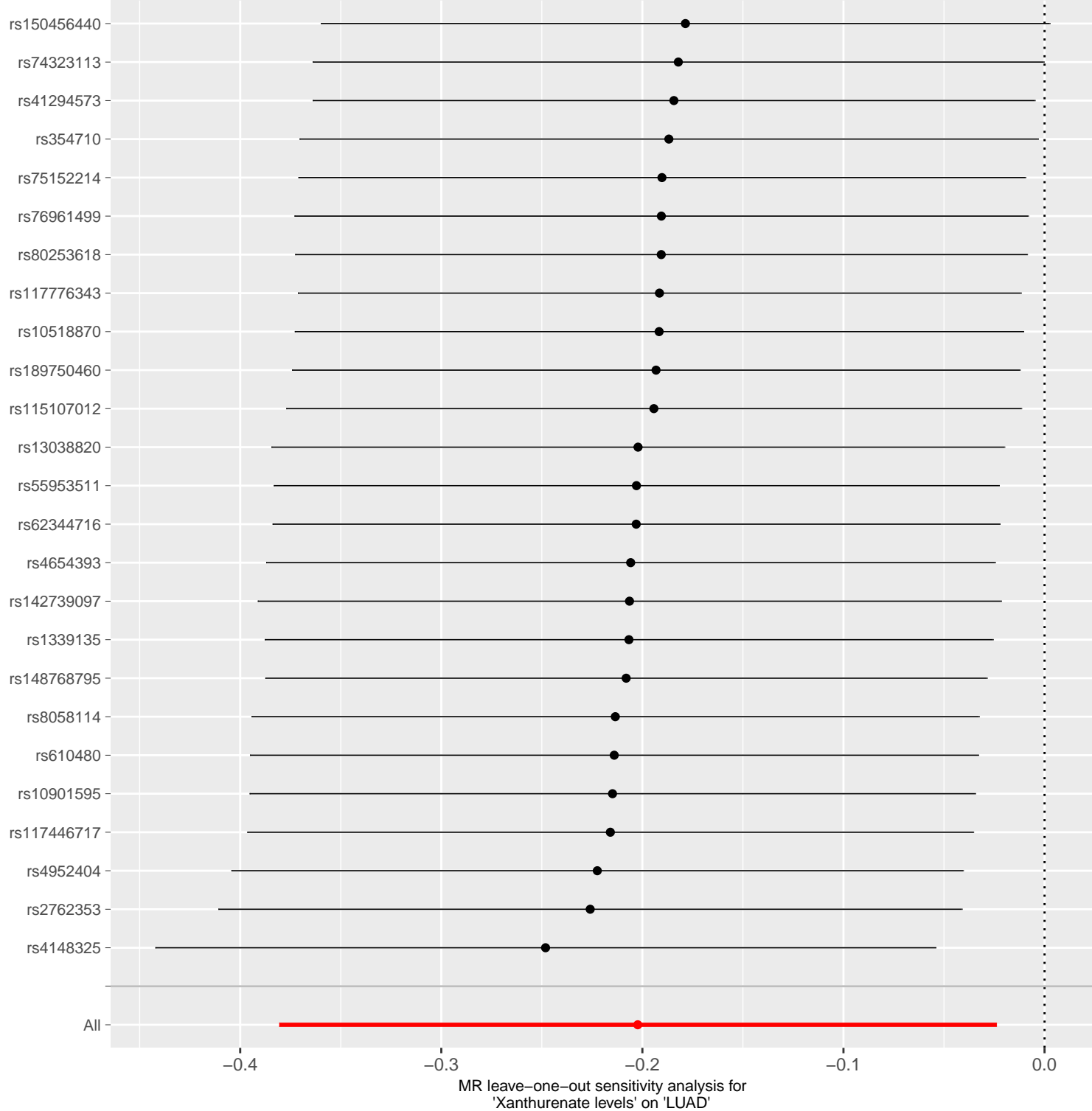

Supplement: Supplementary file 1 [file DataSheet1.zip › supplementary files/S2/GCST90199626/sensitivity-analysis.pdf]

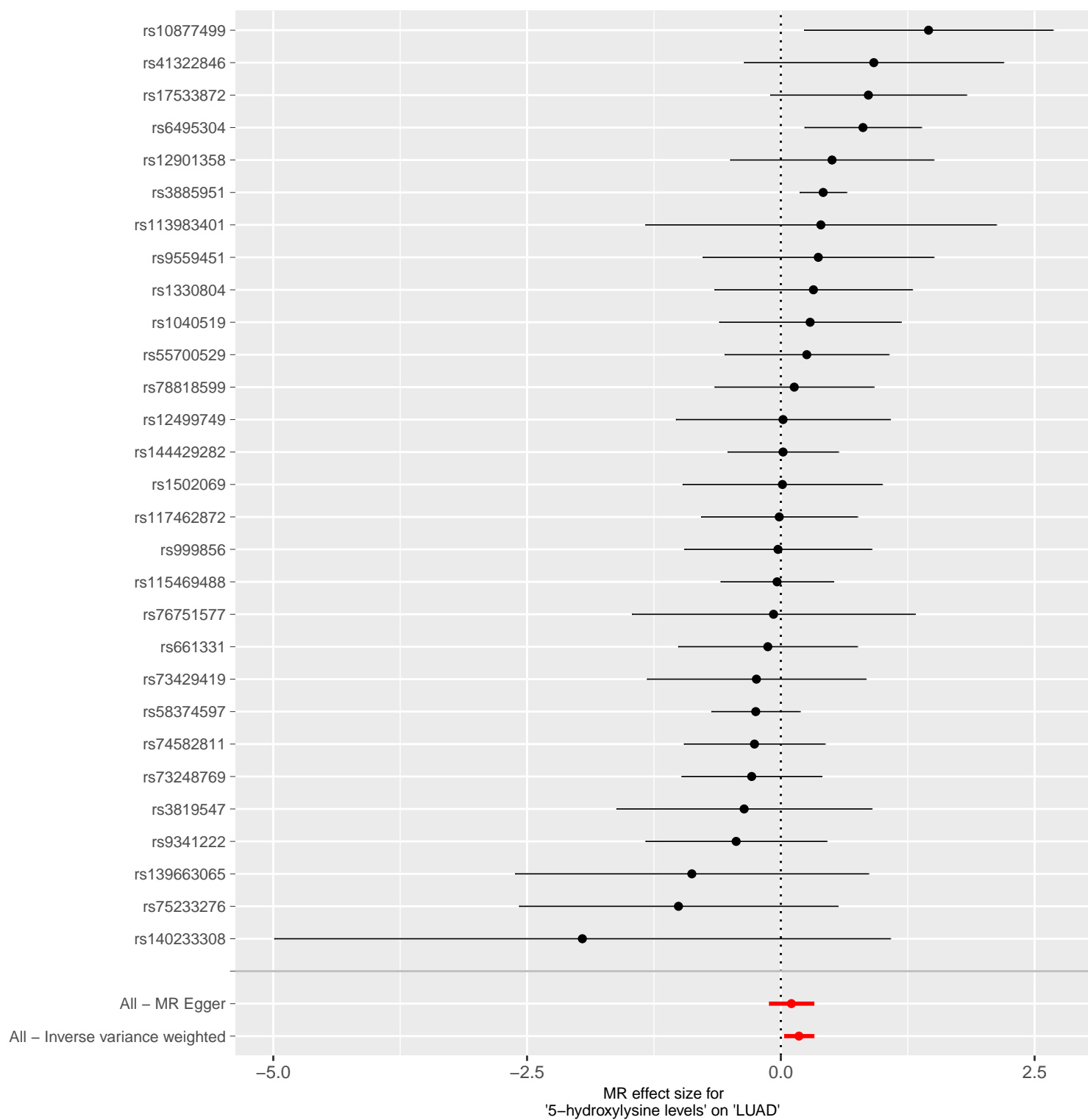

Supplement: Supplementary file 1 [file DataSheet1.zip › supplementary files/S2/GCST90199634/forest.pdf]

# MR Method

- Inverse variance weighted
- MR Egger

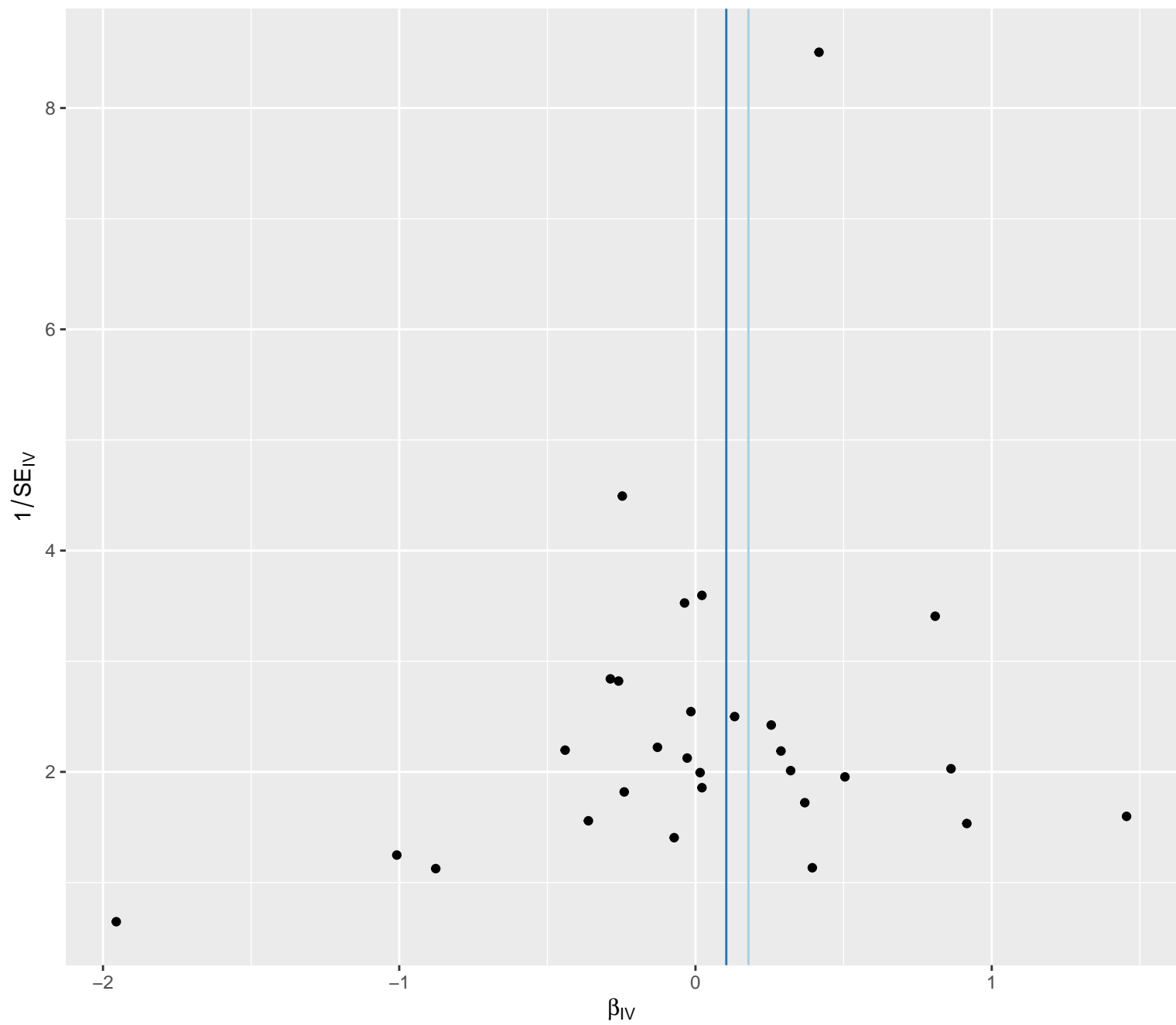

Supplement: Supplementary file 1 [file DataSheet1.zip › supplementary files/S2/GCST90199634/funnelplot.pdf]

# MR Test

- Inverse variance weighted
- MR Egger
- Simple mode
- Weighted median
- Weighted mode

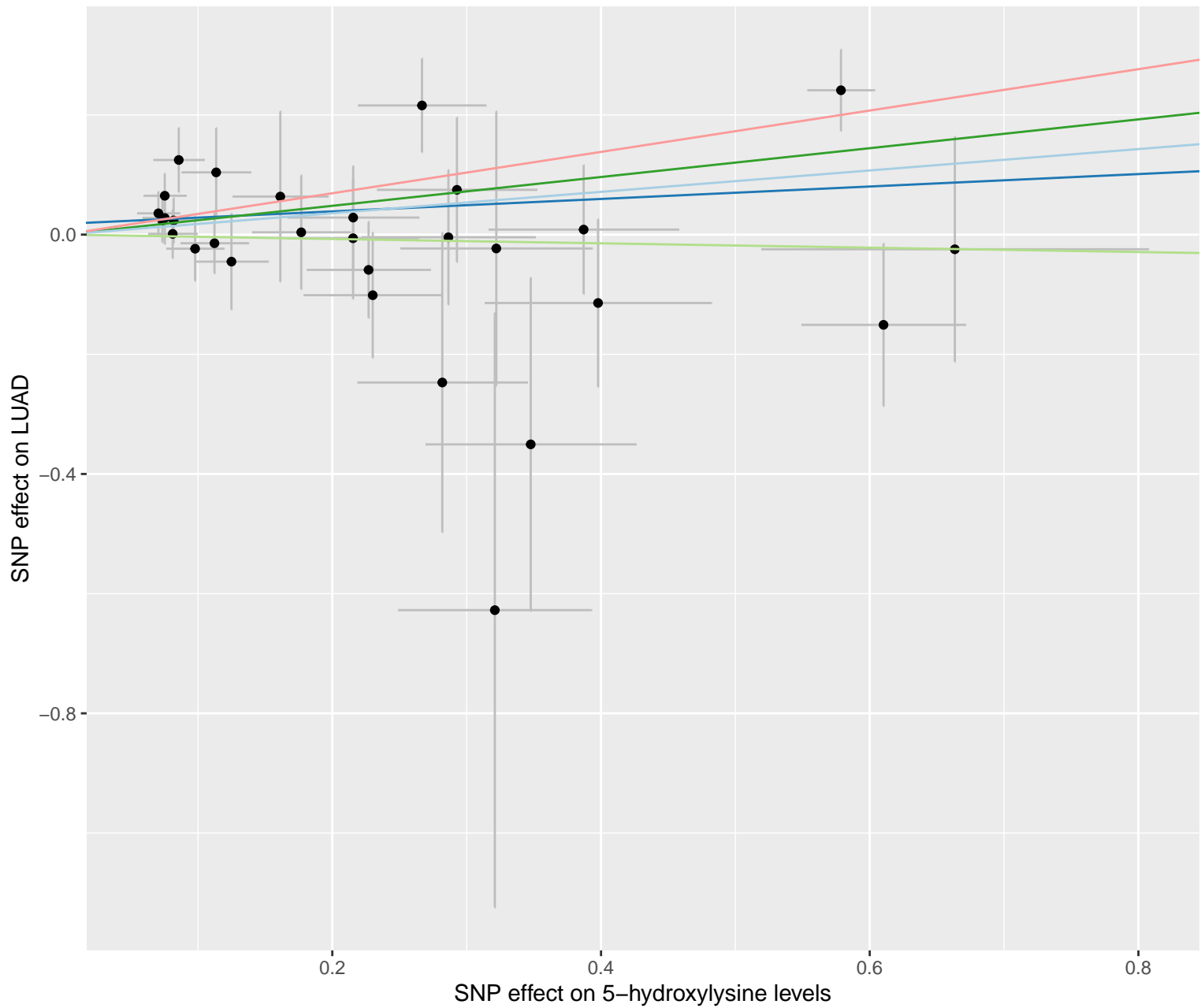

Supplement: Supplementary file 1 [file DataSheet1.zip › supplementary files/S2/GCST90199634/scatter.pdf]

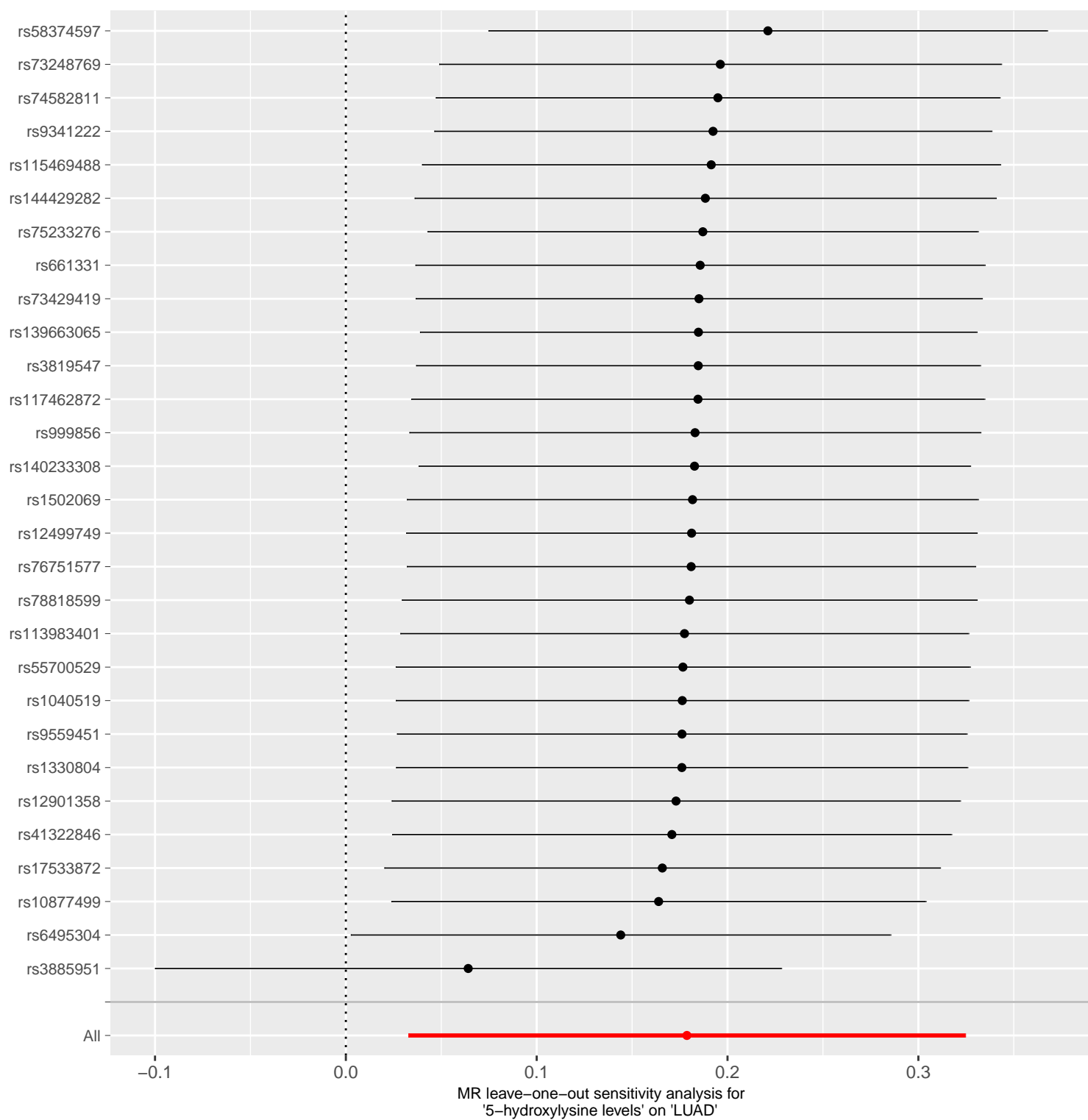

Supplement: Supplementary file 1 [file DataSheet1.zip › supplementary files/S2/GCST90199634/sensitivity-analysis.pdf]

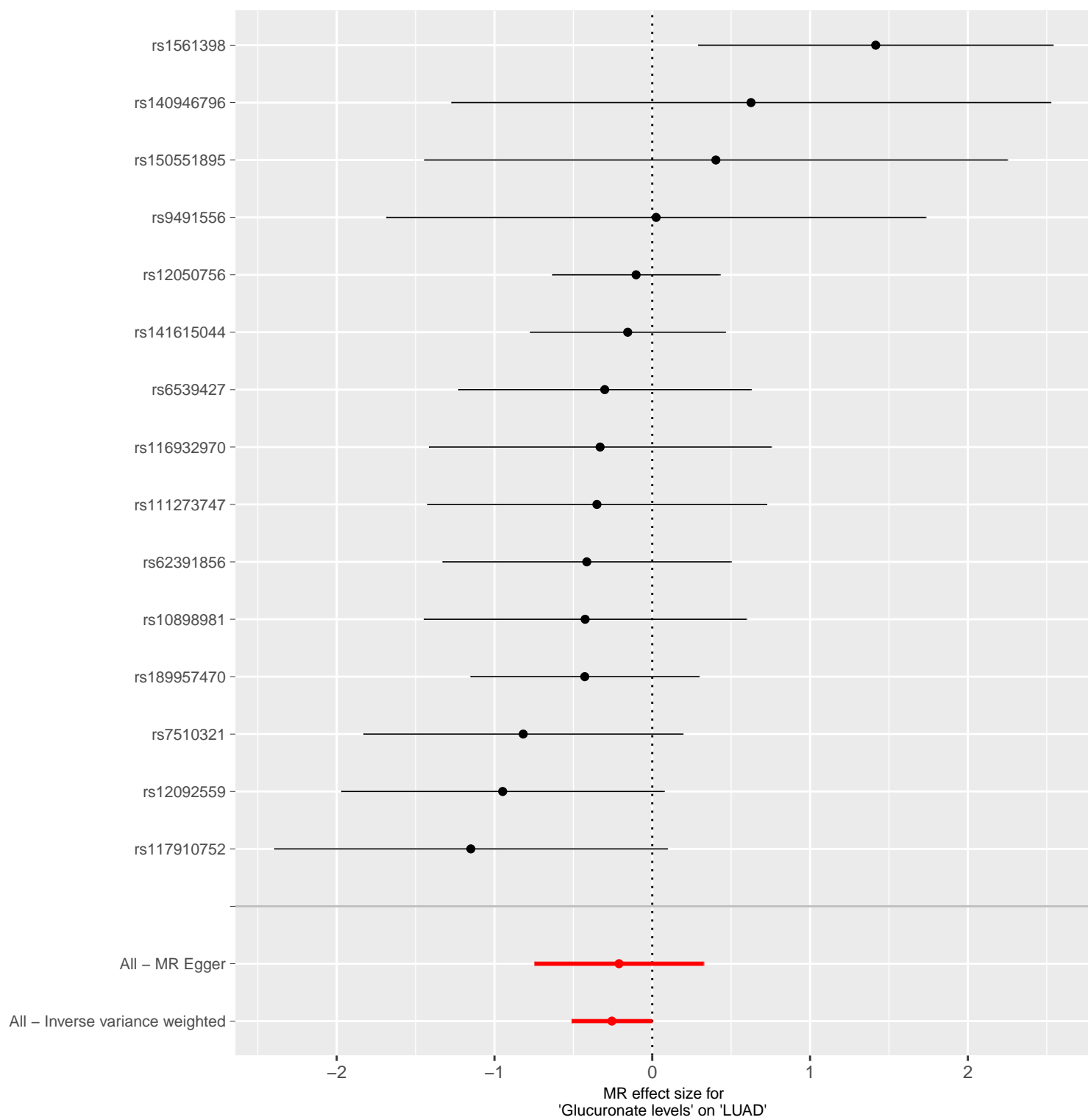

Supplement: Supplementary file 1 [file DataSheet1.zip › supplementary files/S2/GCST90199641/forest.pdf]

# MR Method

- Inverse variance weighted
- MR Egger

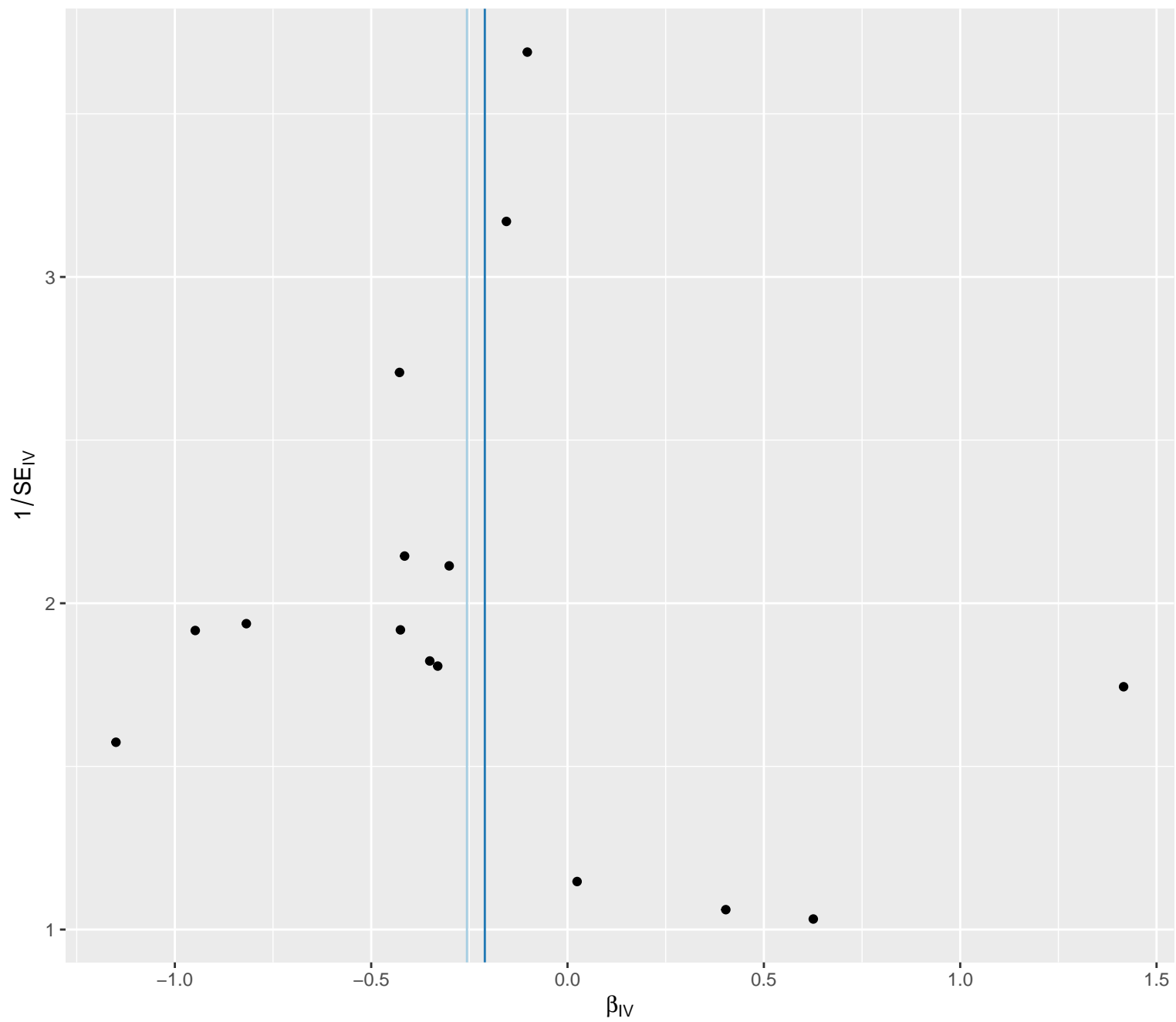

Supplement: Supplementary file 1 [file DataSheet1.zip › supplementary files/S2/GCST90199641/funnelplot.pdf]

# MR Test

- Inverse variance weighted
- MR Egger
- Simple mode
- Weighted median
- Weighted mode

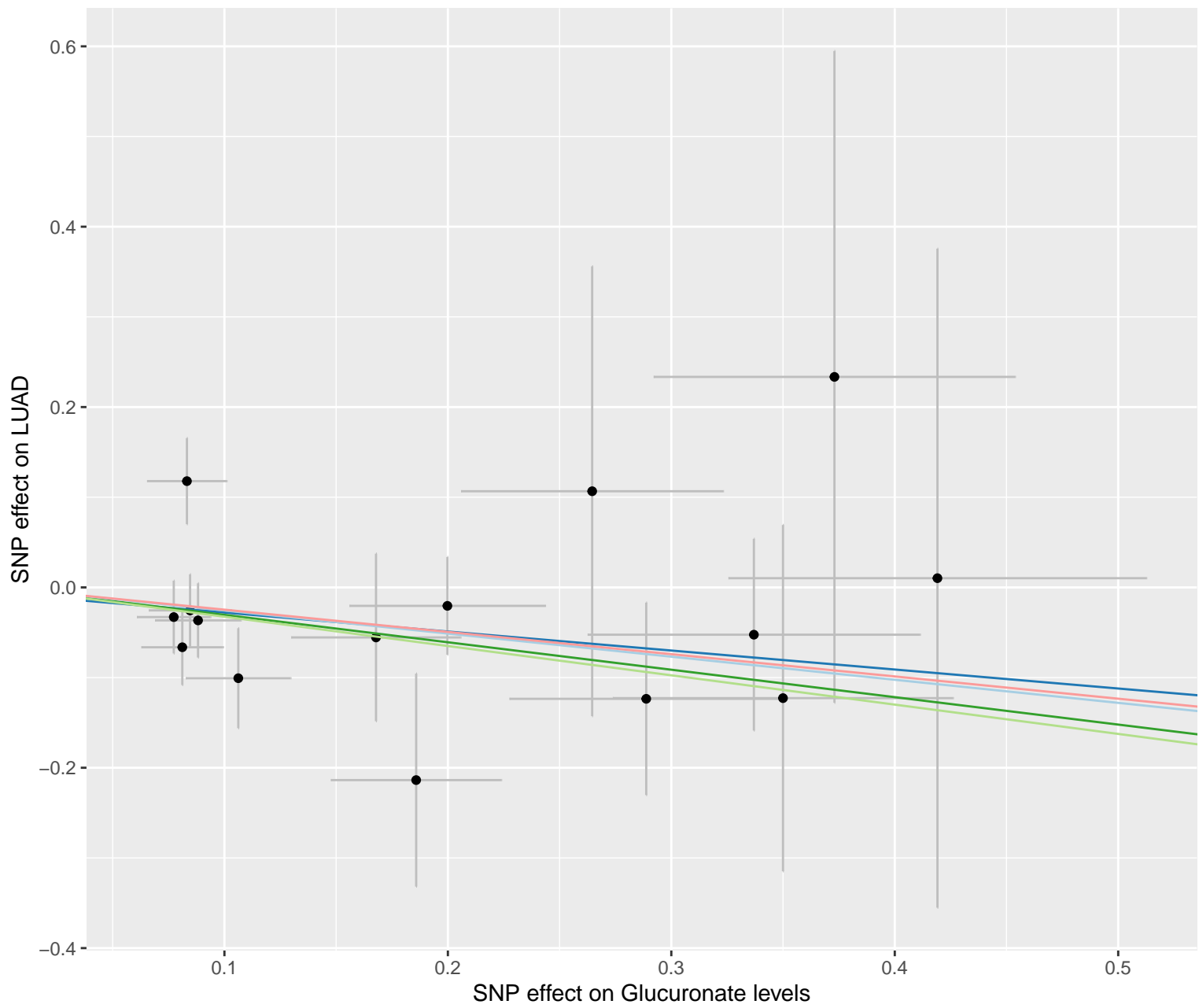

Supplement: Supplementary file 1 [file DataSheet1.zip › supplementary files/S2/GCST90199641/scatter.pdf]

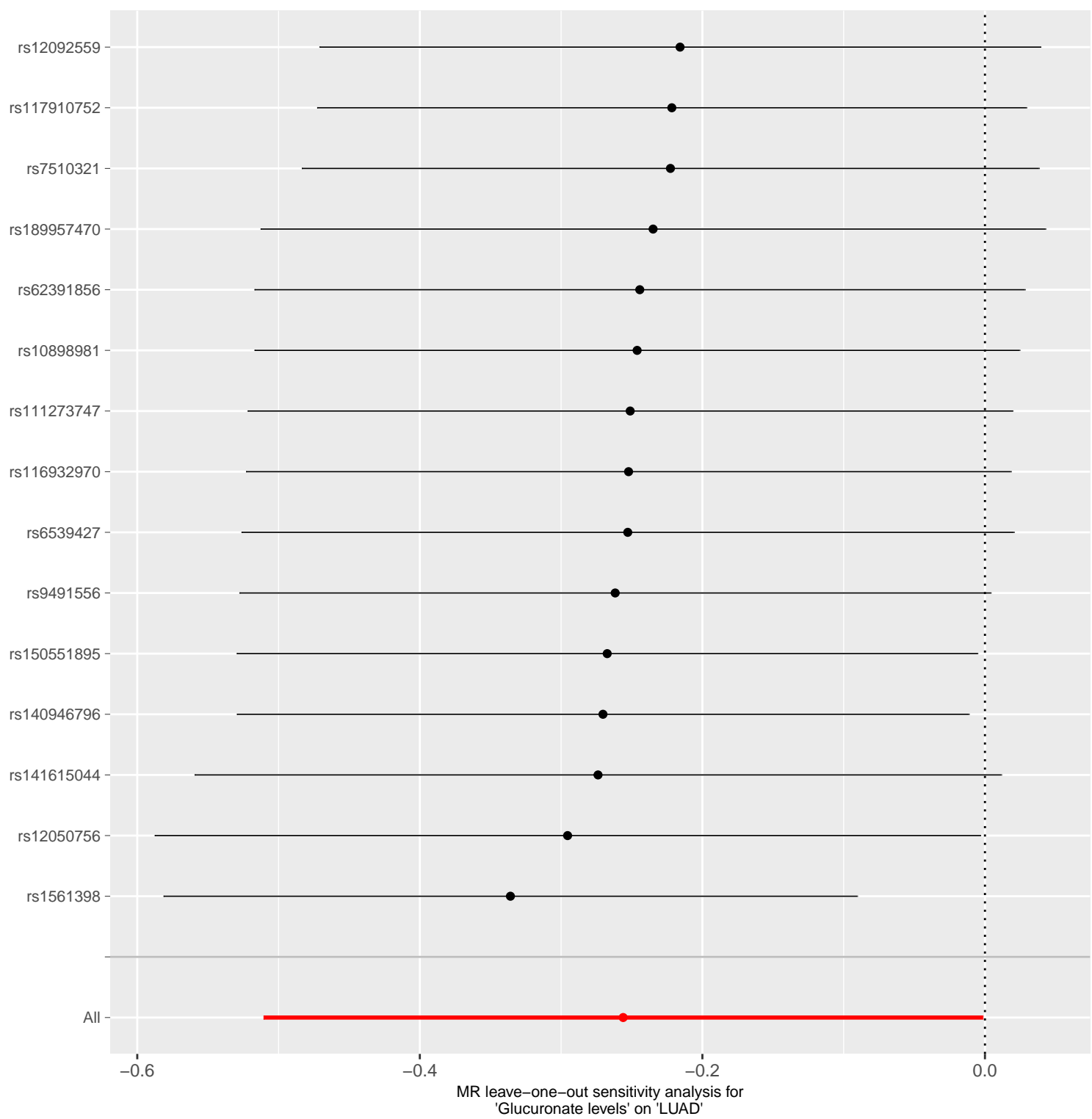

Supplement: Supplementary file 1 [file DataSheet1.zip › supplementary files/S2/GCST90199641/sensitivity-analysis.pdf]

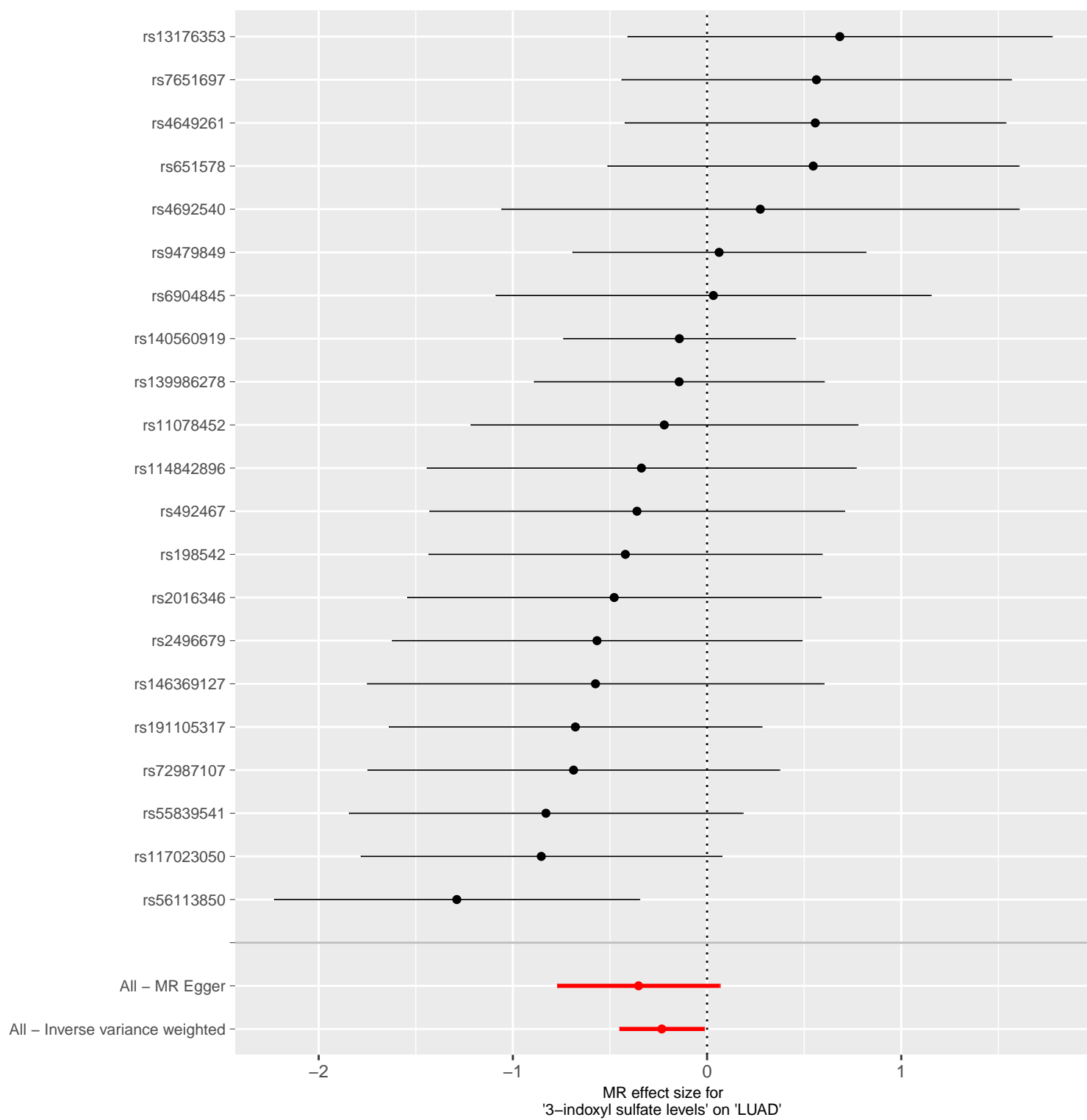

Supplement: Supplementary file 1 [file DataSheet1.zip › supplementary files/S2/GCST90199656/forest.pdf]

# MR Method

- Inverse variance weighted
- MR Egger

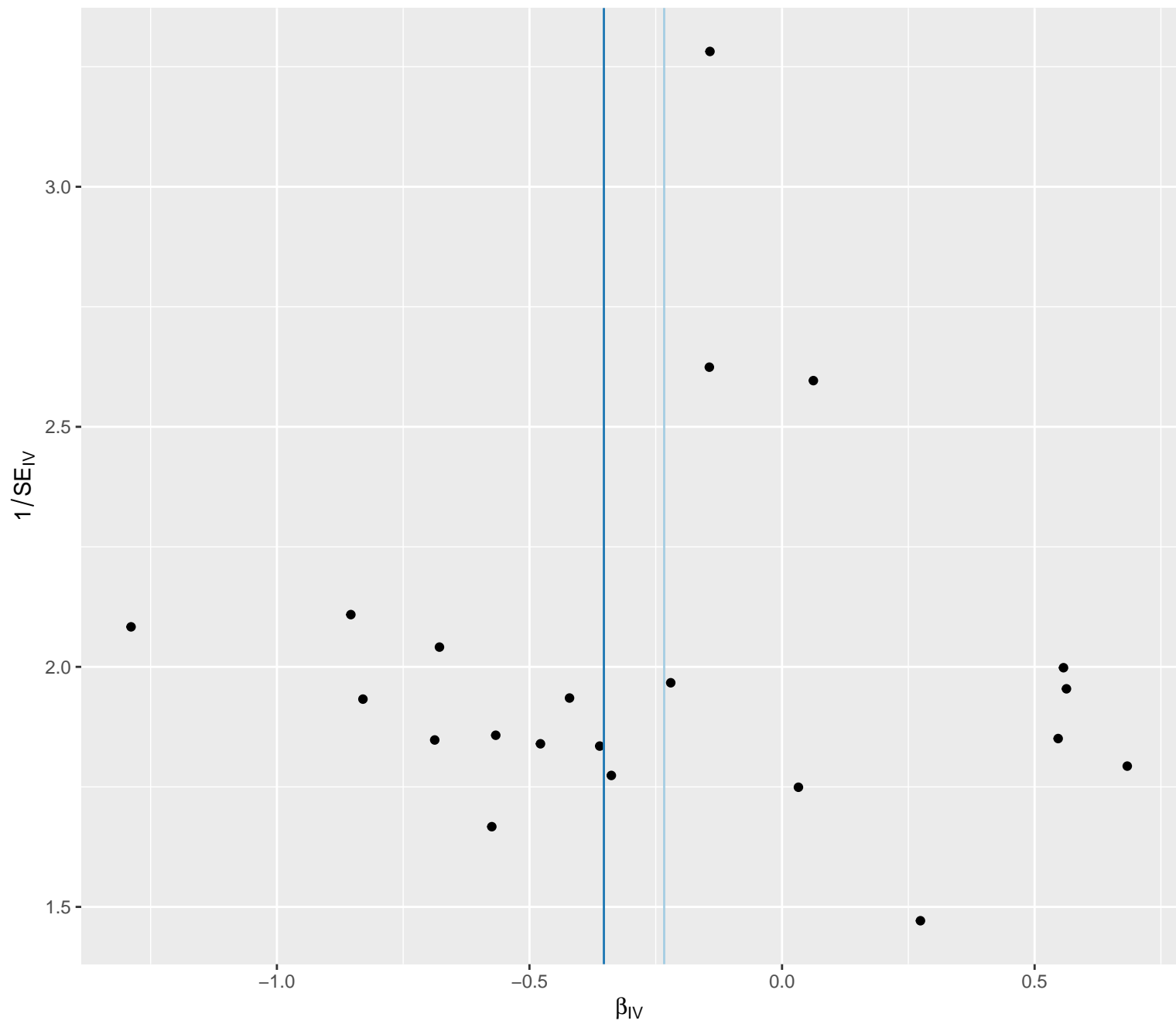

Supplement: Supplementary file 1 [file DataSheet1.zip › supplementary files/S2/GCST90199656/funnelplot.pdf]

# MR Test

- Inverse variance weighted
- MR Egger
- Simple mode
- Weighted median
- Weighted mode

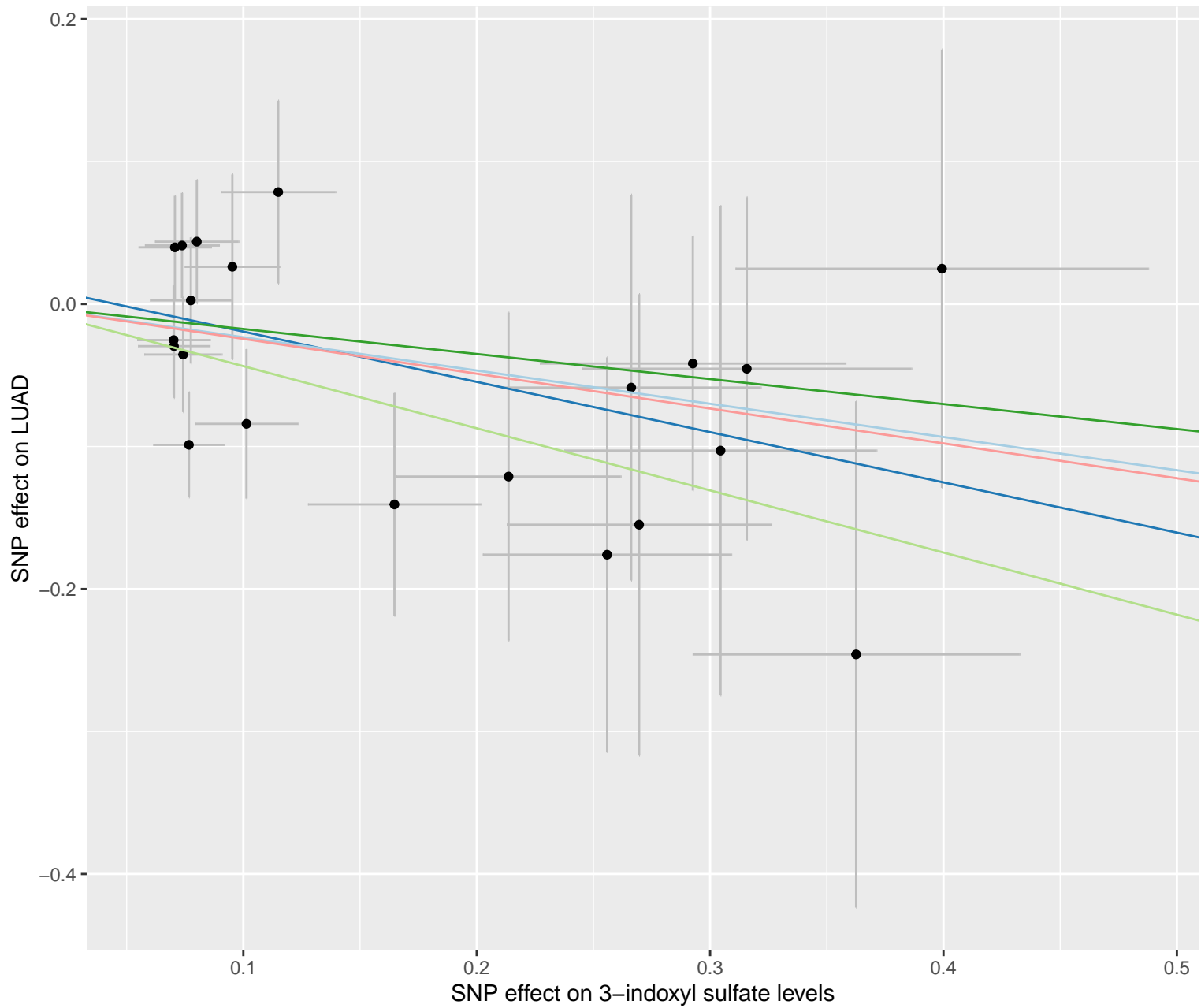

Supplement: Supplementary file 1 [file DataSheet1.zip › supplementary files/S2/GCST90199656/scatter.pdf]

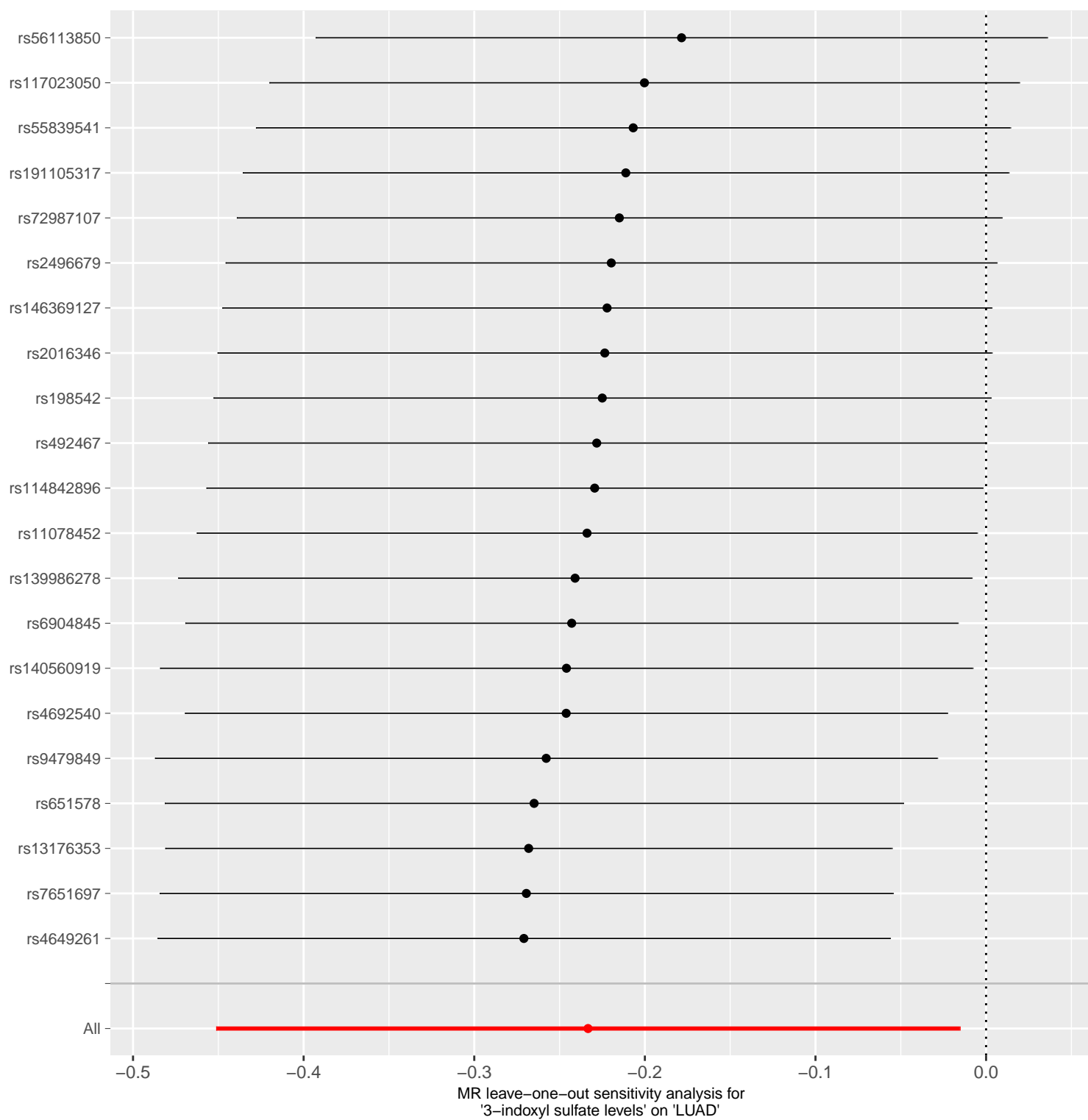

Supplement: Supplementary file 1 [file DataSheet1.zip › supplementary files/S2/GCST90199656/sensitivity-analysis.pdf]

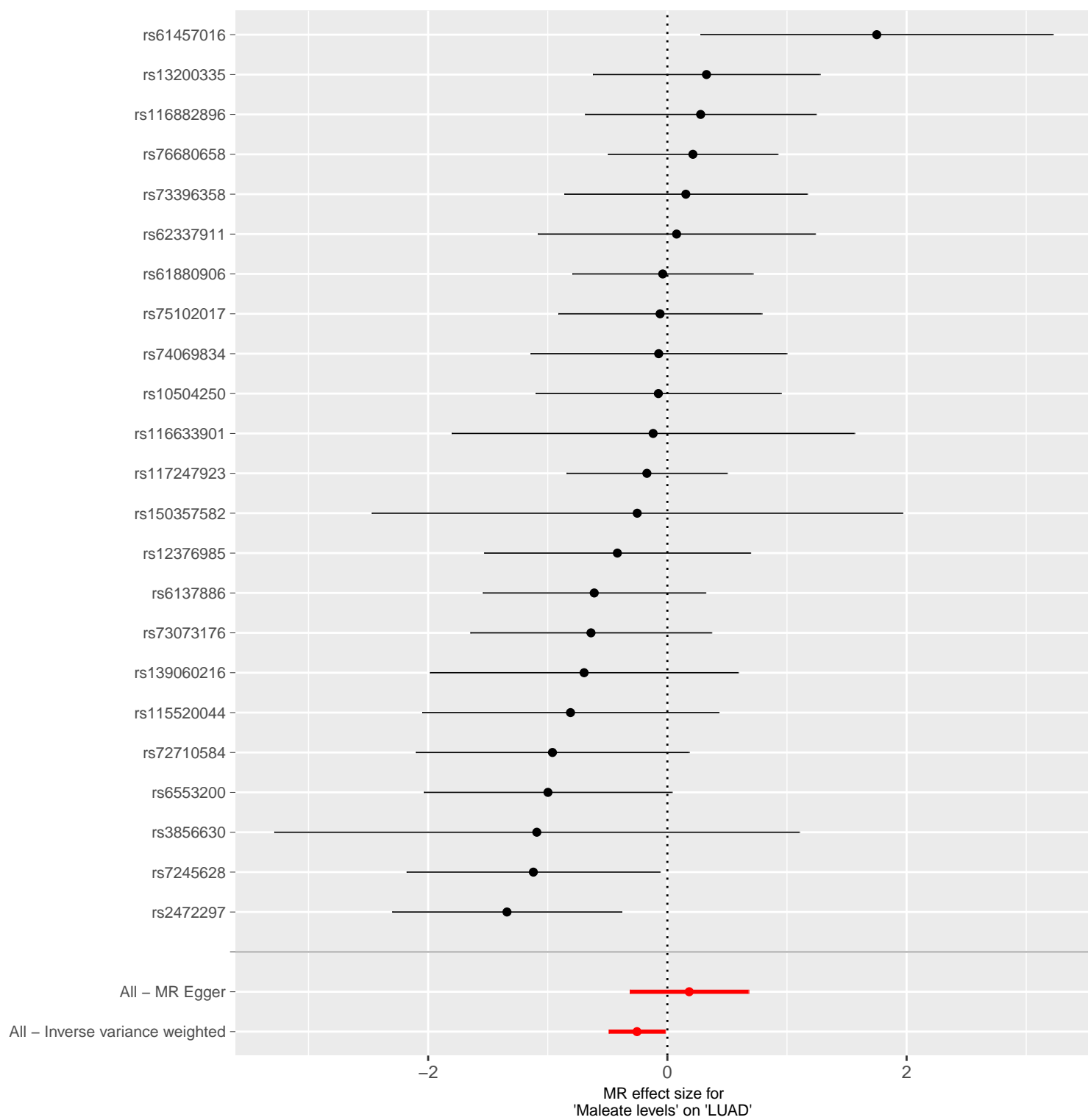

Supplement: Supplementary file 1 [file DataSheet1.zip › supplementary files/S2/GCST90199661/forest.pdf]

# MR Method

- Inverse variance weighted
- MR Egger

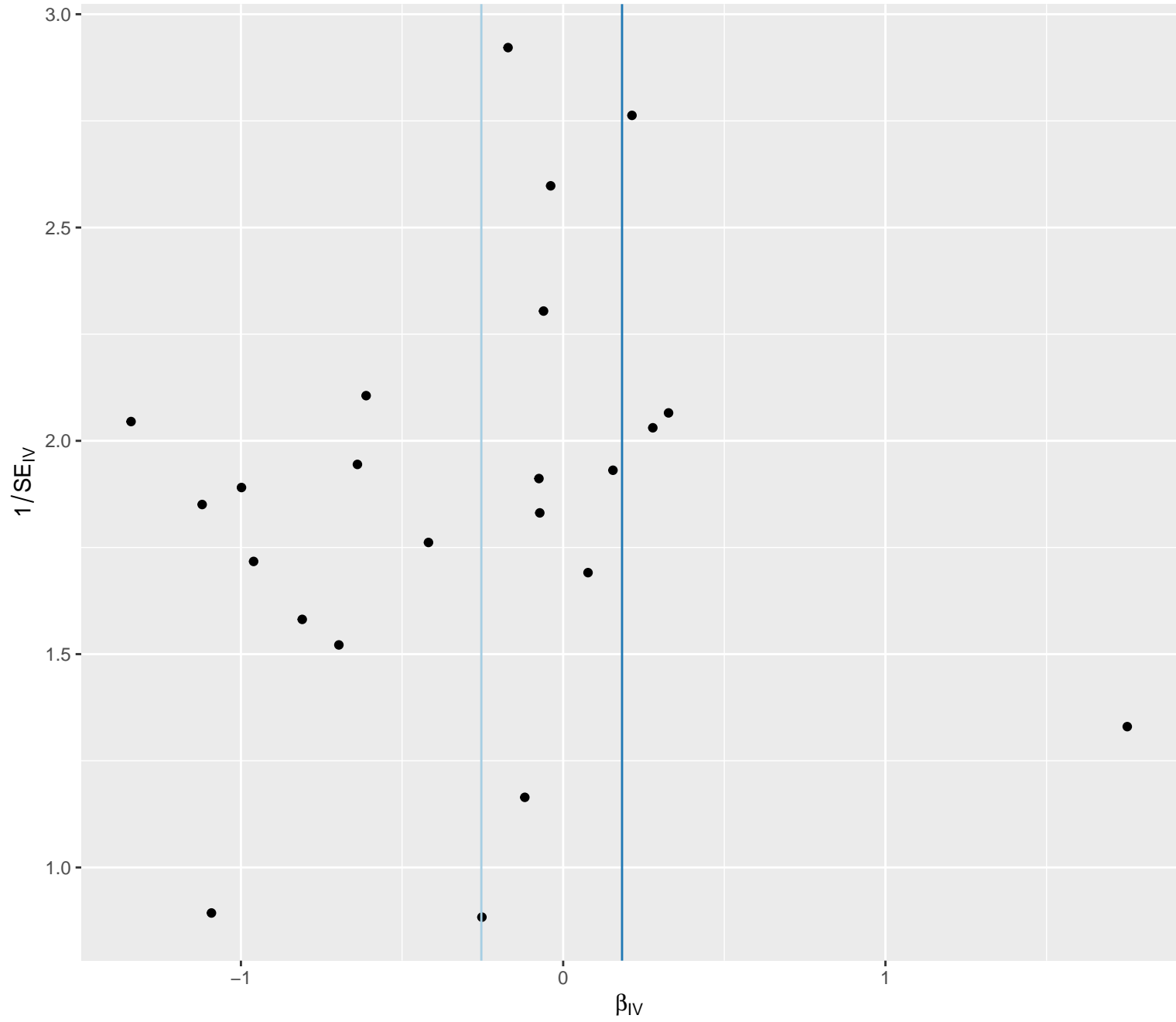

Supplement: Supplementary file 1 [file DataSheet1.zip › supplementary files/S2/GCST90199661/funnelplot.pdf]

# MR Test

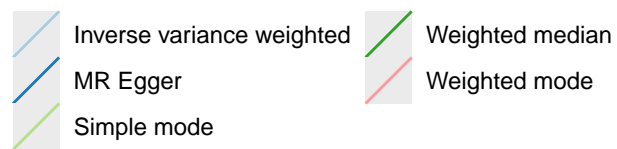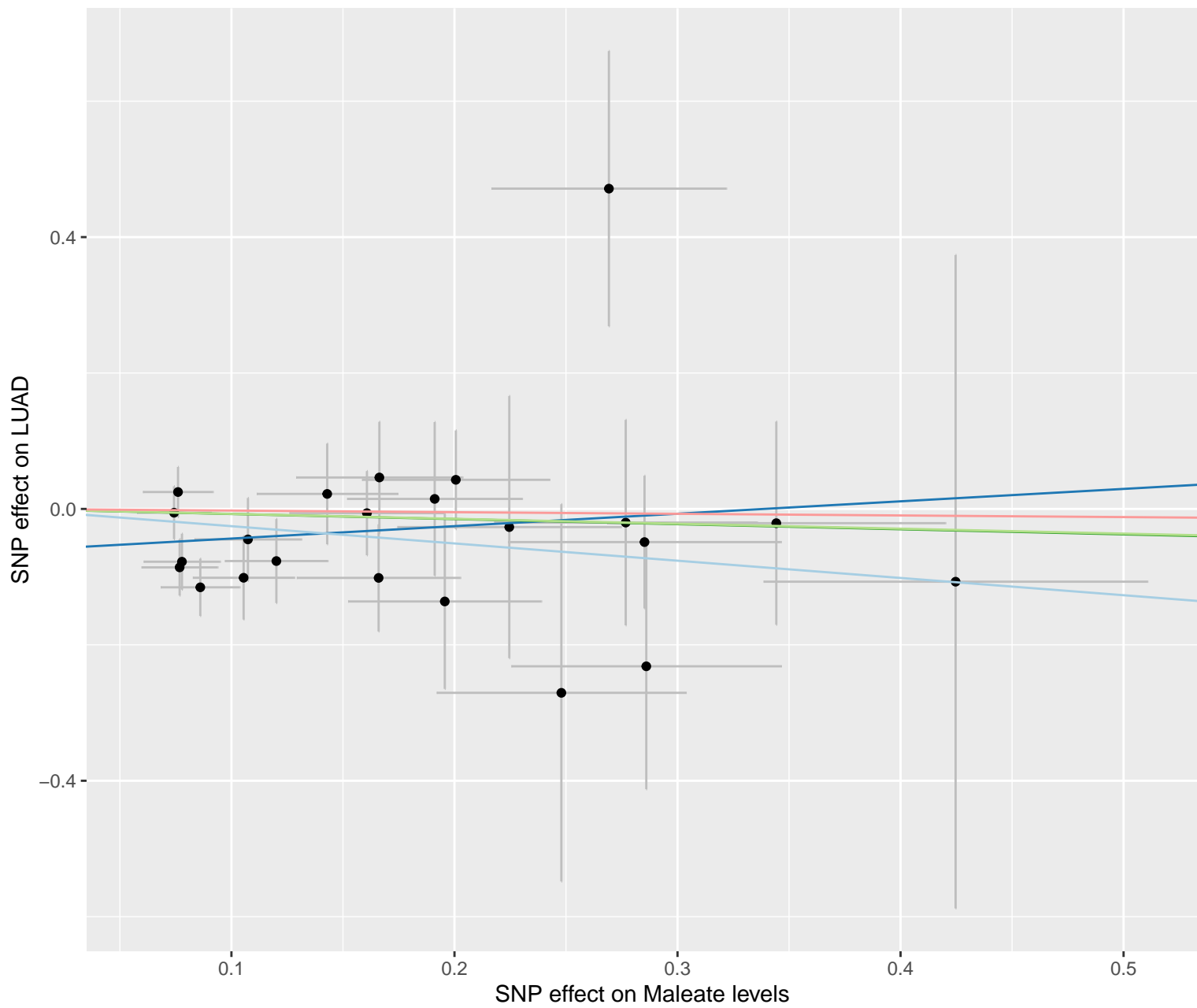

Supplement: Supplementary file 1 [file DataSheet1.zip › supplementary files/S2/GCST90199661/scatter.pdf]

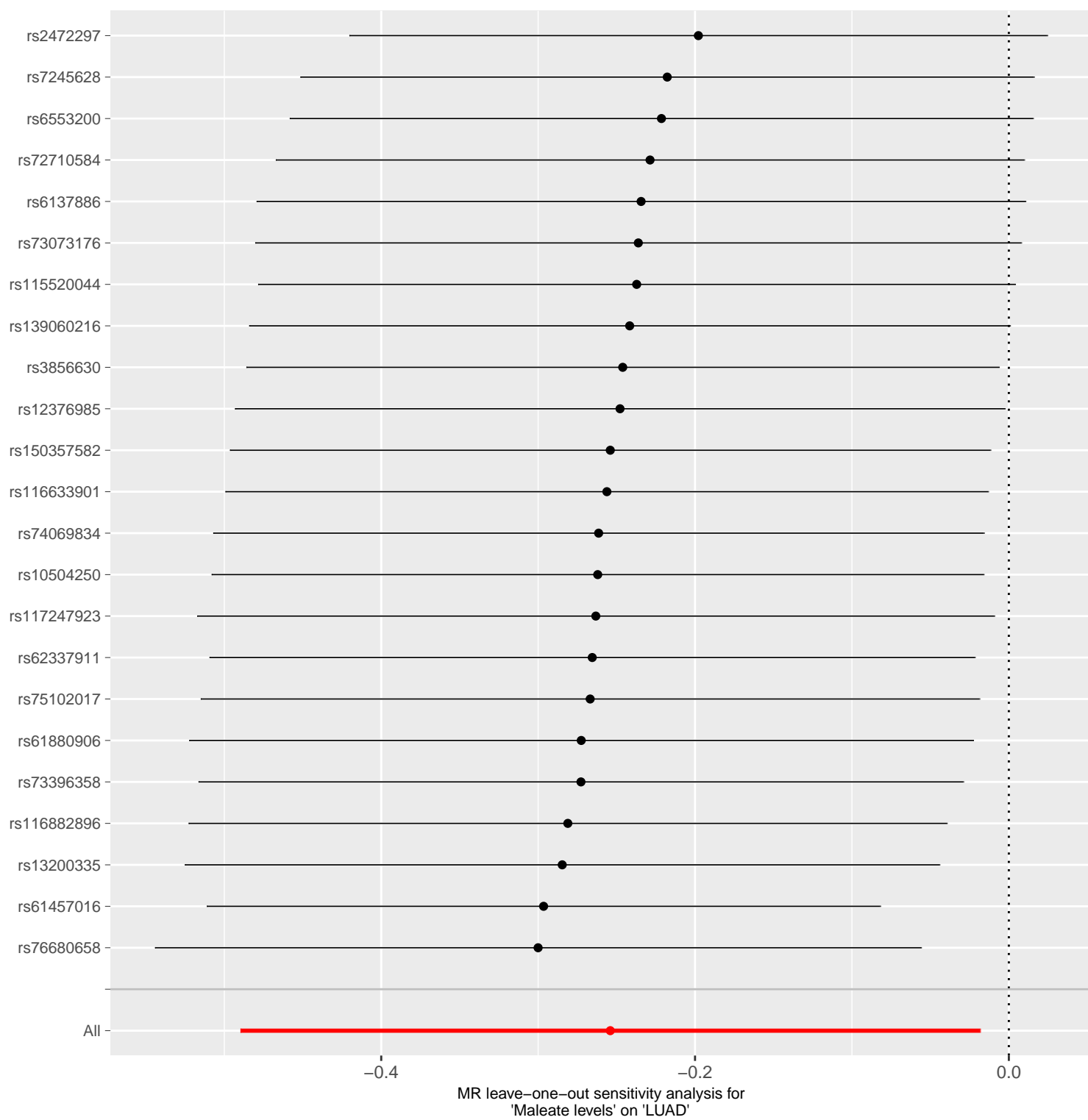

Supplement: Supplementary file 1 [file DataSheet1.zip › supplementary files/S2/GCST90199661/sensitivity-analysis.pdf]

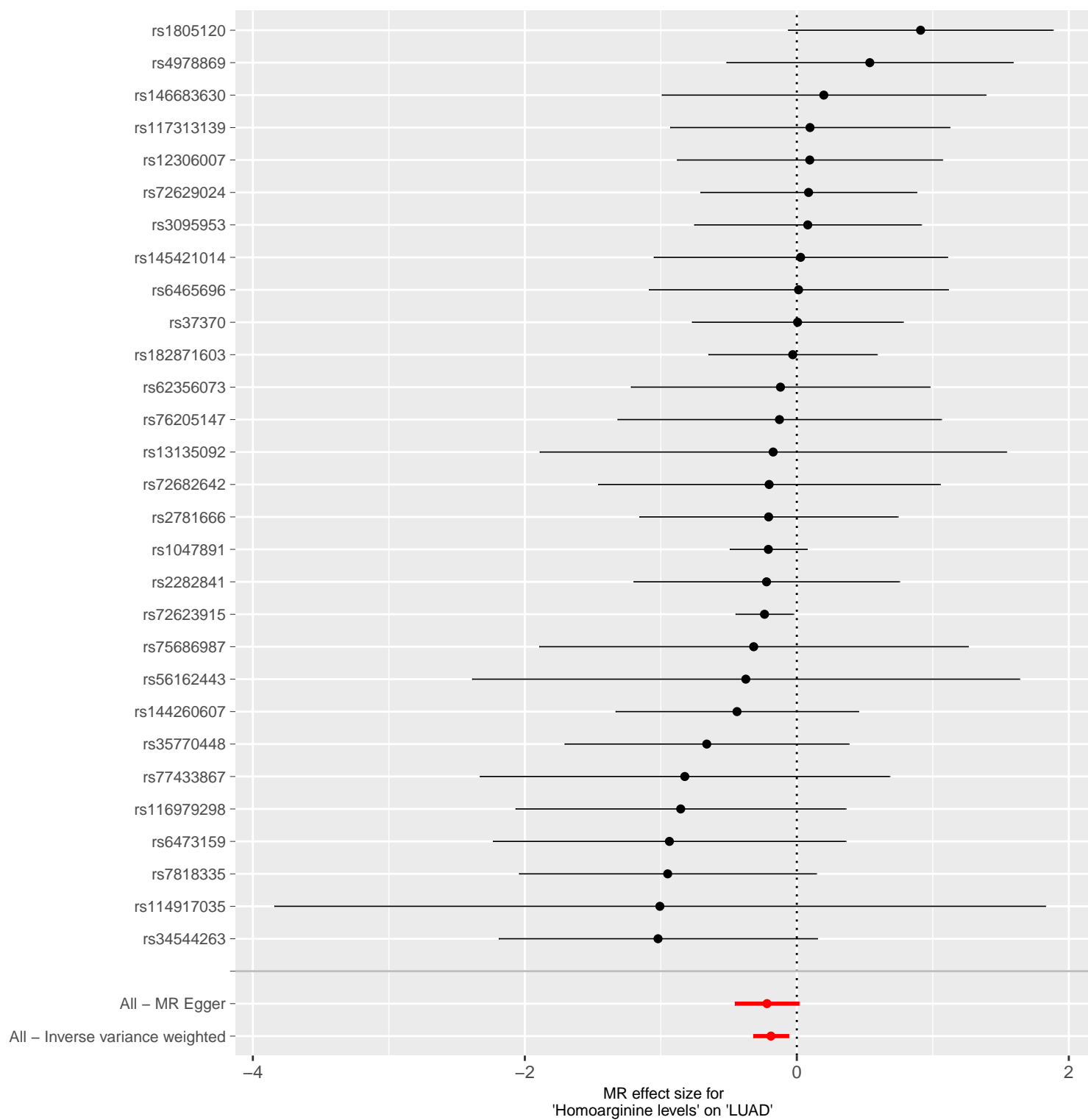

Supplement: Supplementary file 1 [file DataSheet1.zip › supplementary files/S2/GCST90199675/forest.pdf]

# MR Method

- Inverse variance weighted
- MR Egger

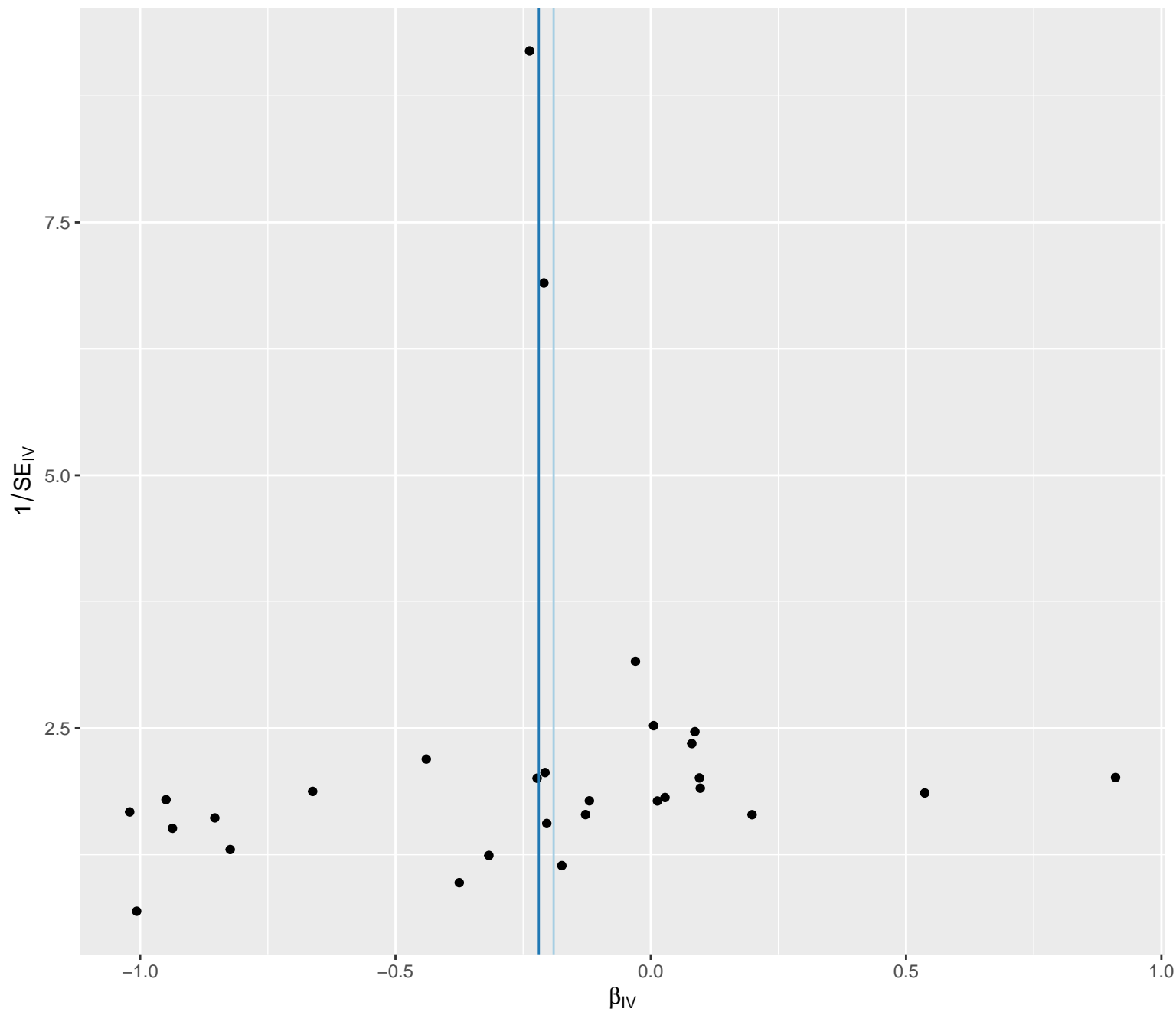

Supplement: Supplementary file 1 [file DataSheet1.zip › supplementary files/S2/GCST90199675/funnelplot.pdf]

# MR Test

- Inverse variance weighted
- MR Egger
- Simple mode
- Weighted median
- Weighted mode

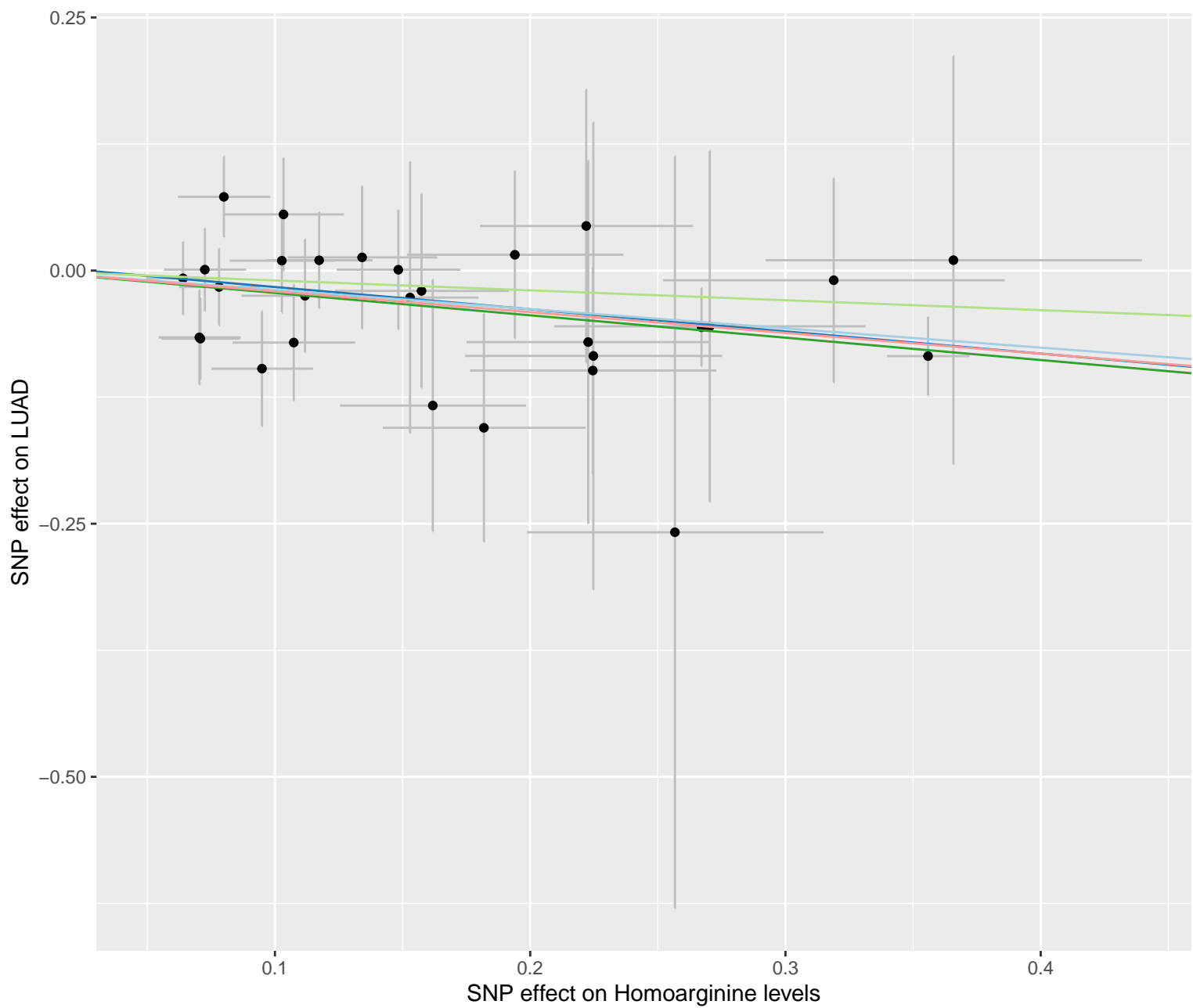

Supplement: Supplementary file 1 [file DataSheet1.zip › supplementary files/S2/GCST90199675/scatter.pdf]

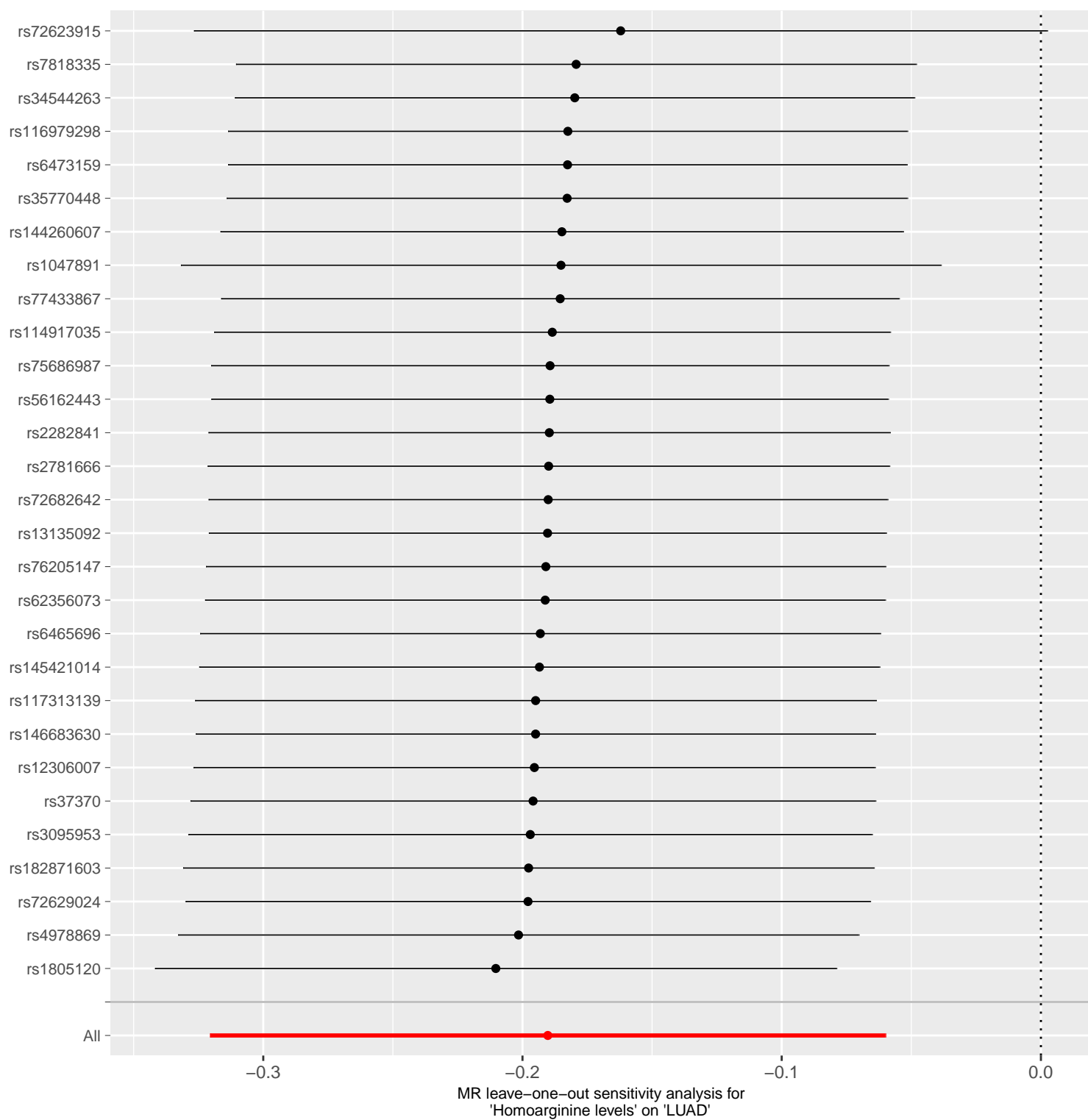

Supplement: Supplementary file 1 [file DataSheet1.zip › supplementary files/S2/GCST90199675/sensitivity-analysis.pdf]

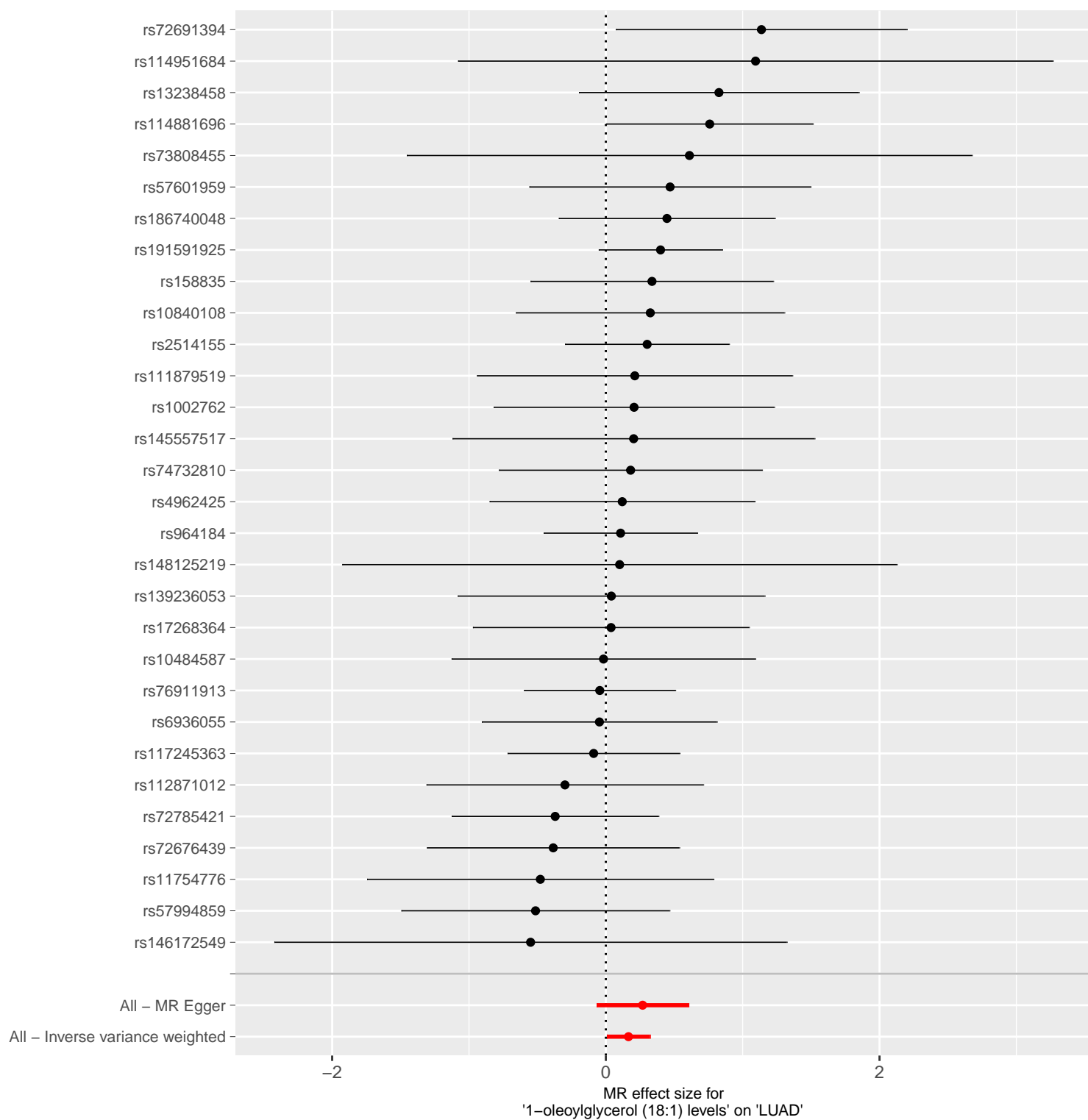

Supplement: Supplementary file 1 [file DataSheet1.zip › supplementary files/S2/GCST90199676/forest.pdf]

# MR Method

- Inverse variance weighted
- MR Egger

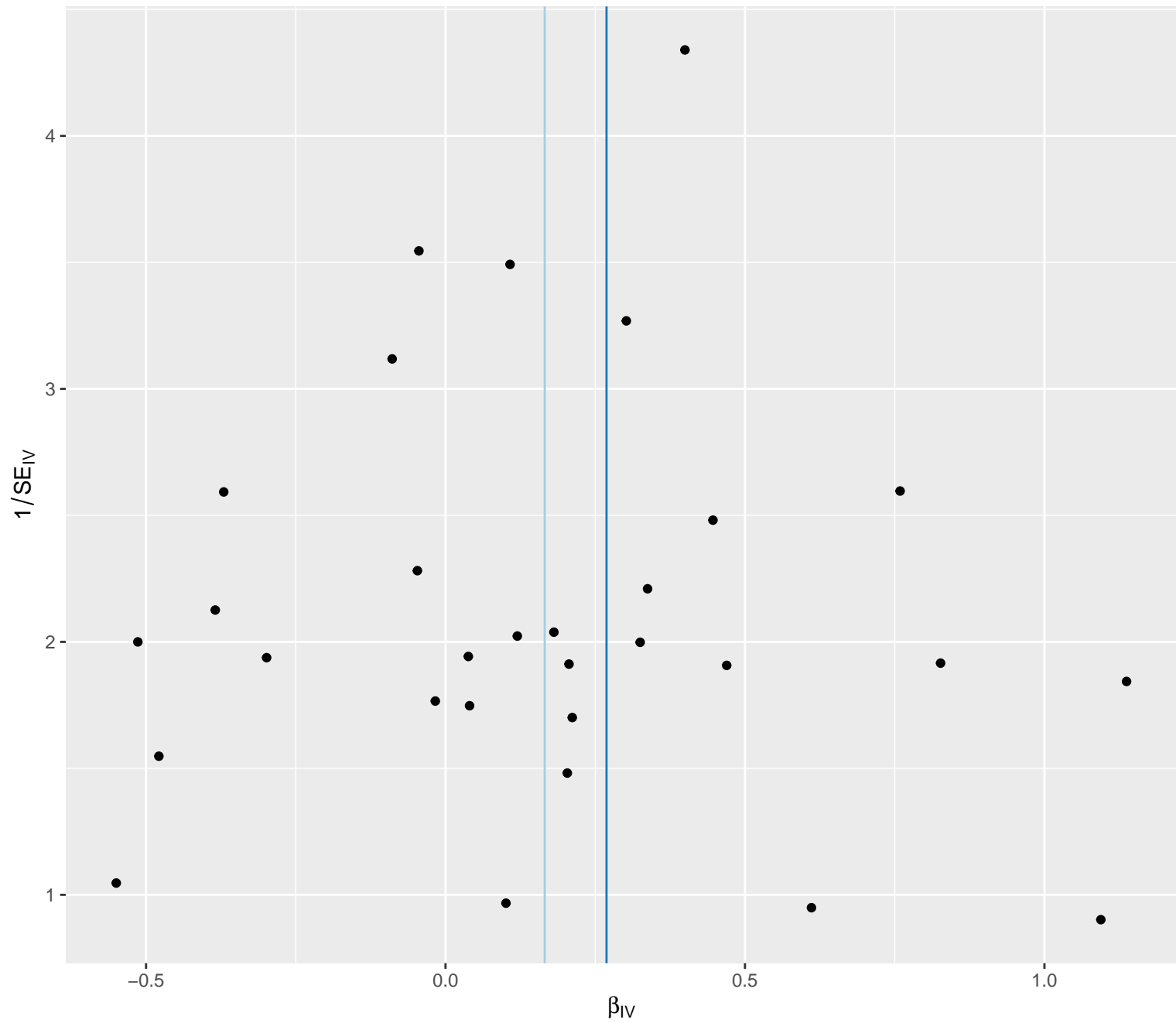

Supplement: Supplementary file 1 [file DataSheet1.zip › supplementary files/S2/GCST90199676/funnelplot.pdf]

# MR Test

- Inverse variance weighted
- MR Egger
- Simple mode
- Weighted median
- Weighted mode

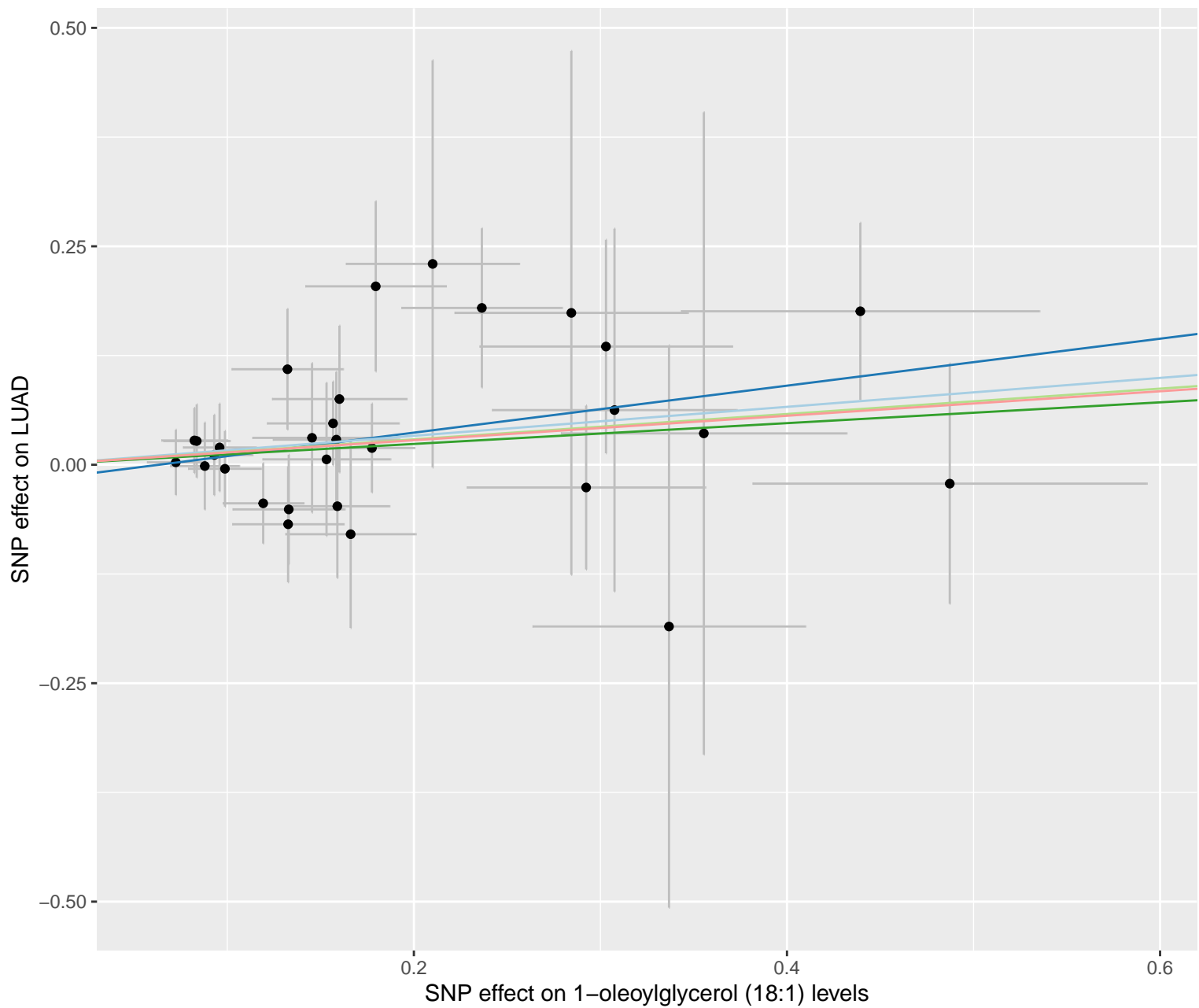

Supplement: Supplementary file 1 [file DataSheet1.zip › supplementary files/S2/GCST90199676/scatter.pdf]

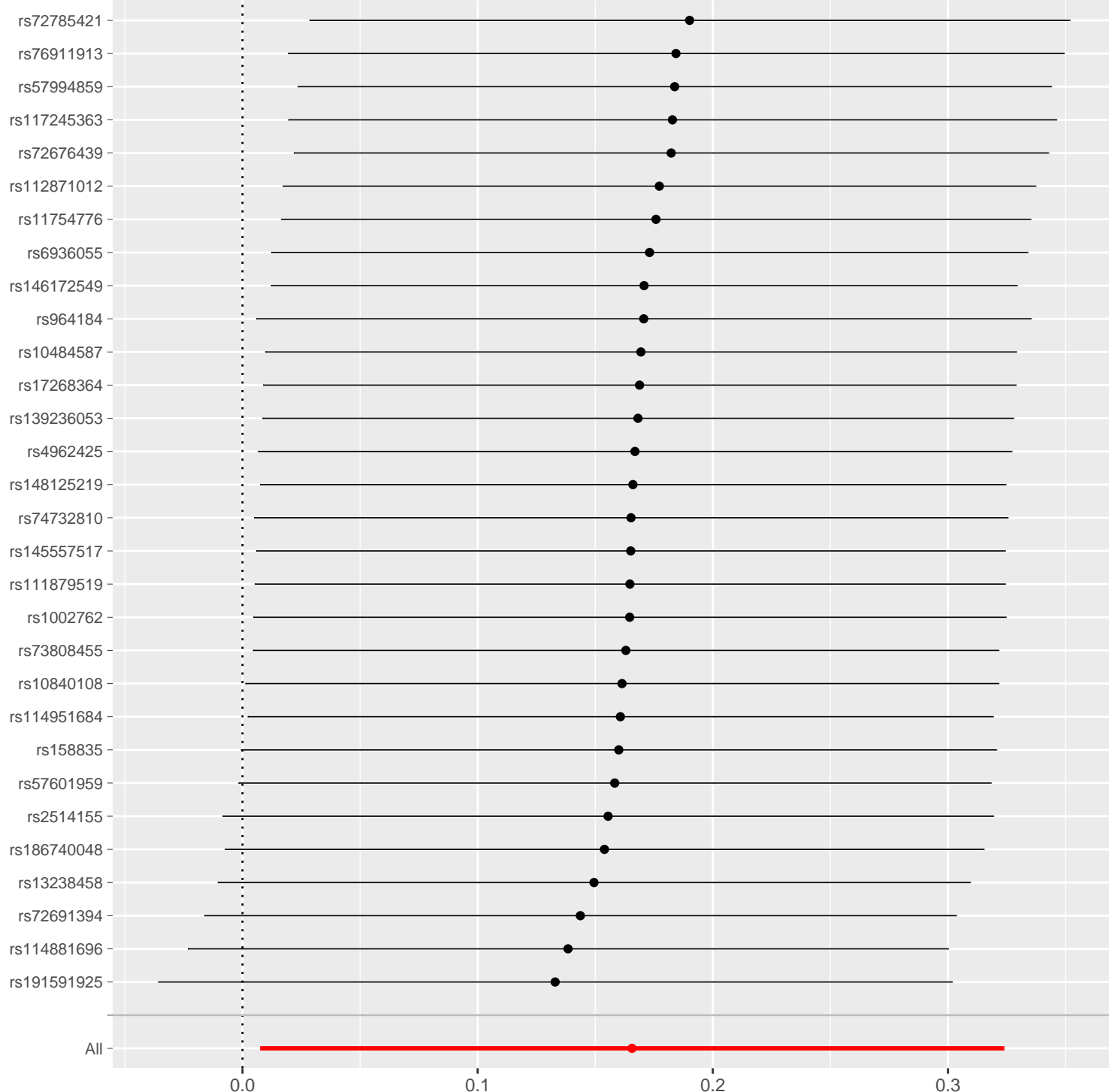

Supplement: Supplementary file 1 [file DataSheet1.zip › supplementary files/S2/GCST90199676/sensitivity-analysis.pdf]

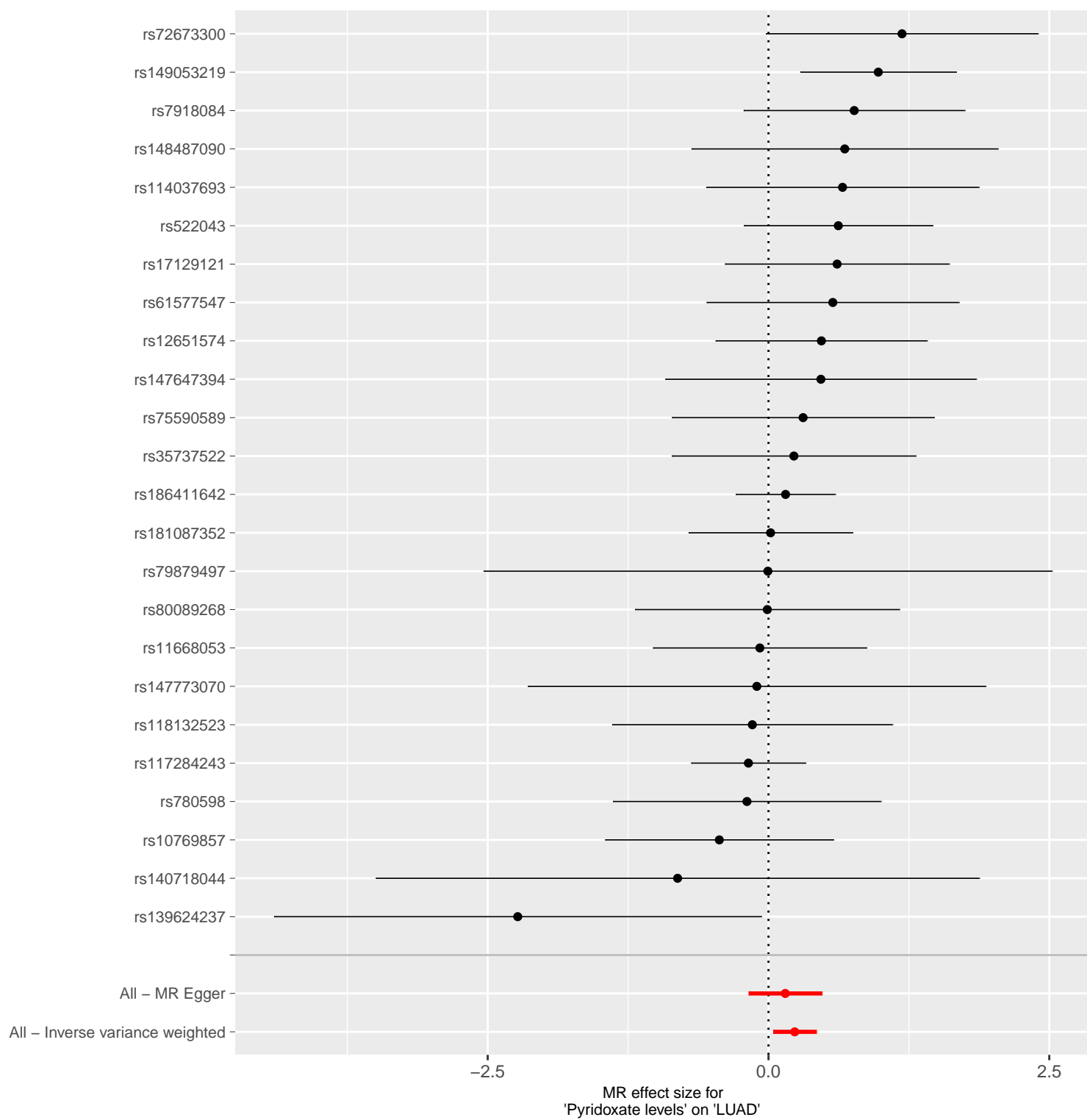

Supplement: Supplementary file 1 [file DataSheet1.zip › supplementary files/S2/GCST90199701/forest.pdf]

# MR Method

- Inverse variance weighted
- MR Egger

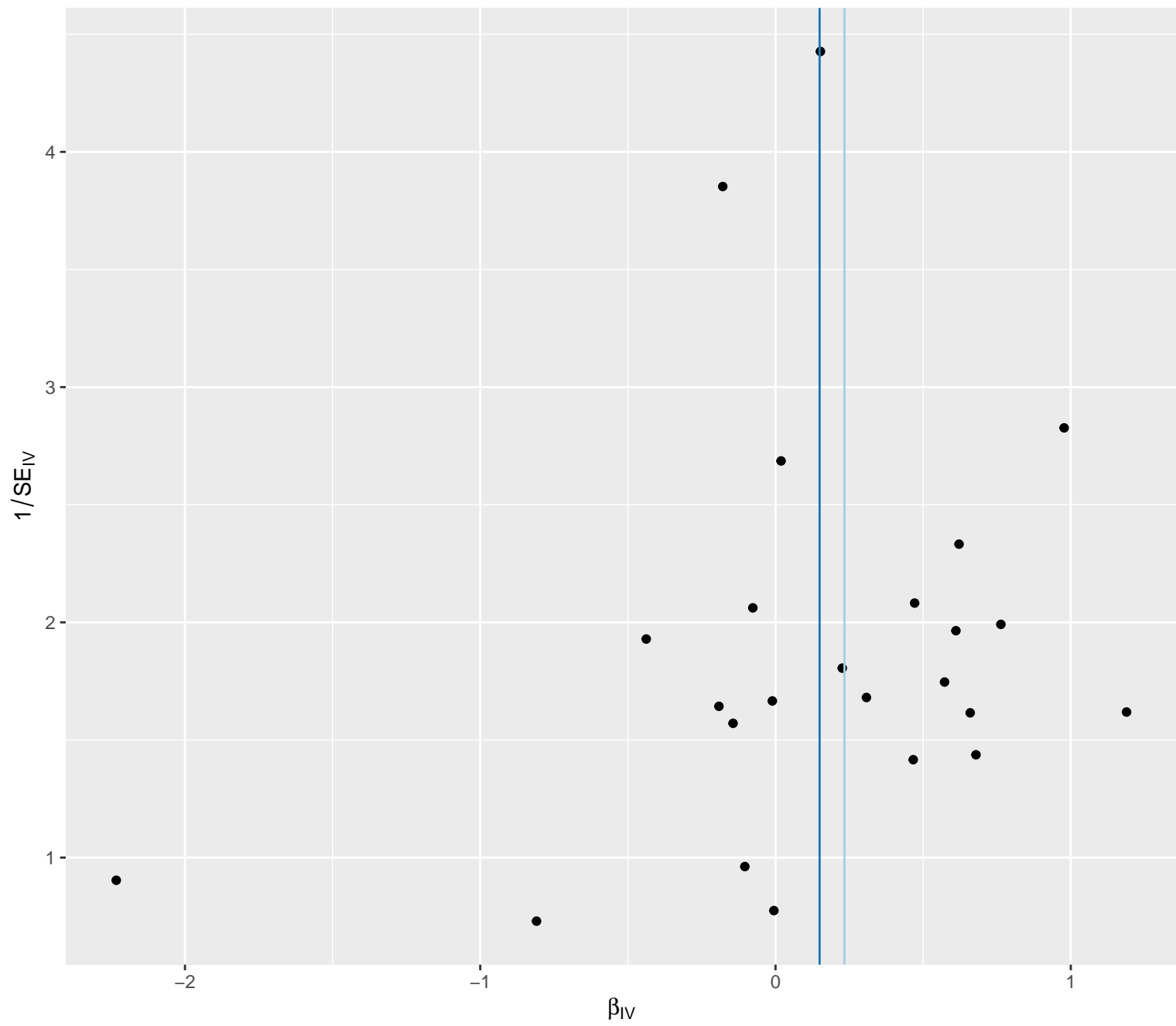

Supplement: Supplementary file 1 [file DataSheet1.zip › supplementary files/S2/GCST90199701/funnelplot.pdf]

# MR Test

- Inverse variance weighted
- MR Egger
- Simple mode
- Weighted median
- Weighted mode

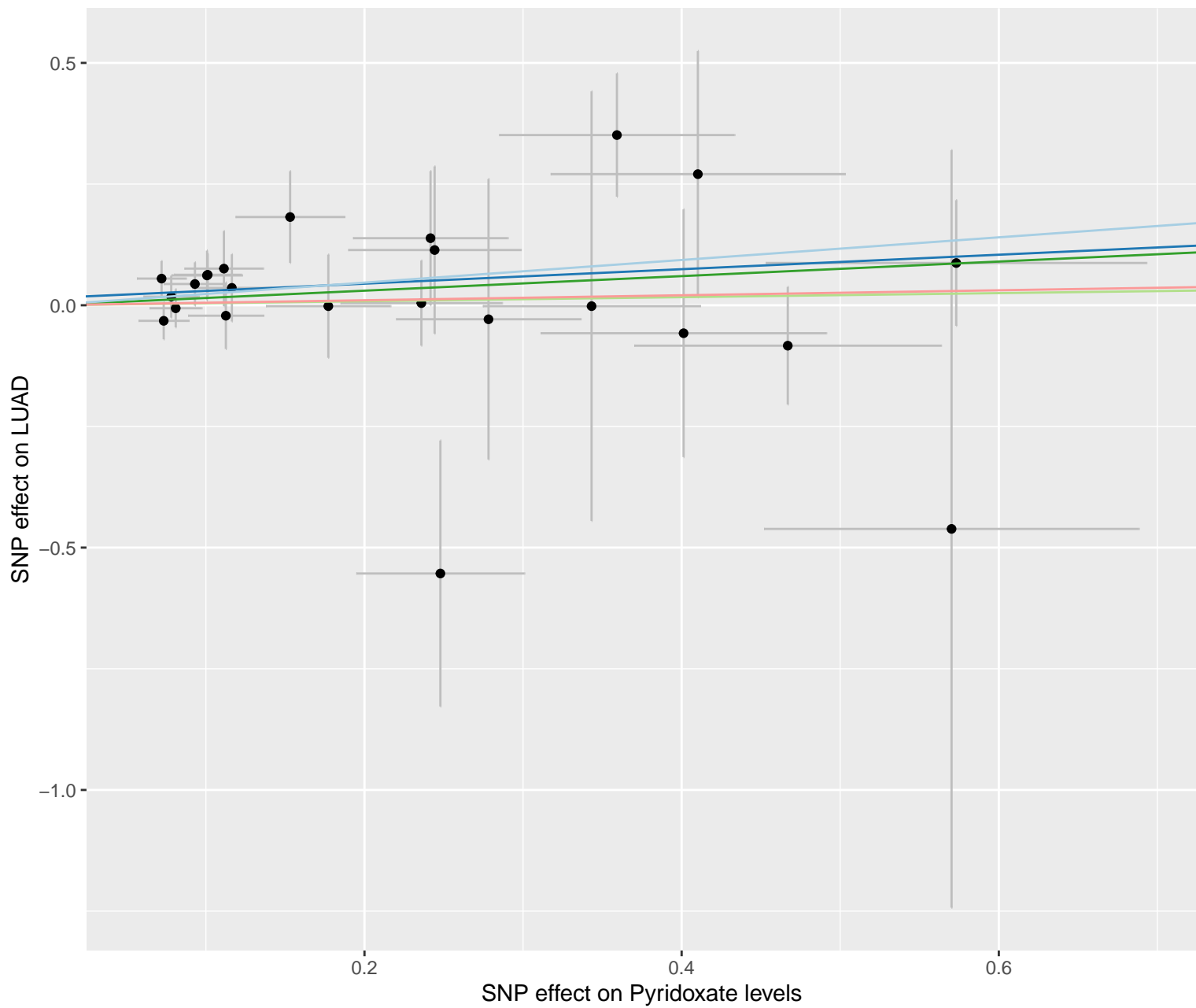

Supplement: Supplementary file 1 [file DataSheet1.zip › supplementary files/S2/GCST90199701/scatter.pdf]

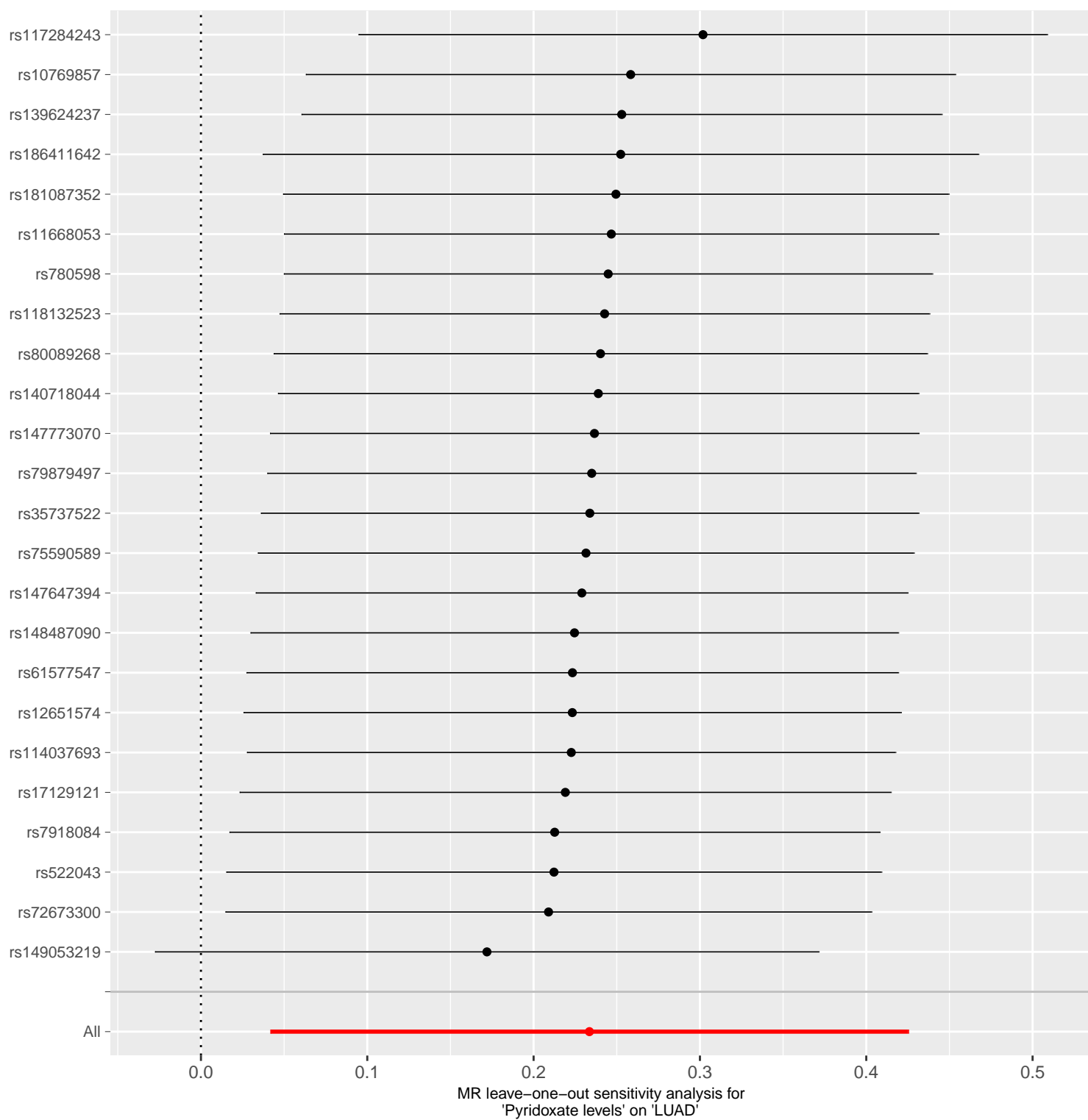

Supplement: Supplementary file 1 [file DataSheet1.zip › supplementary files/S2/GCST90199701/sensitivity-analysis.pdf]

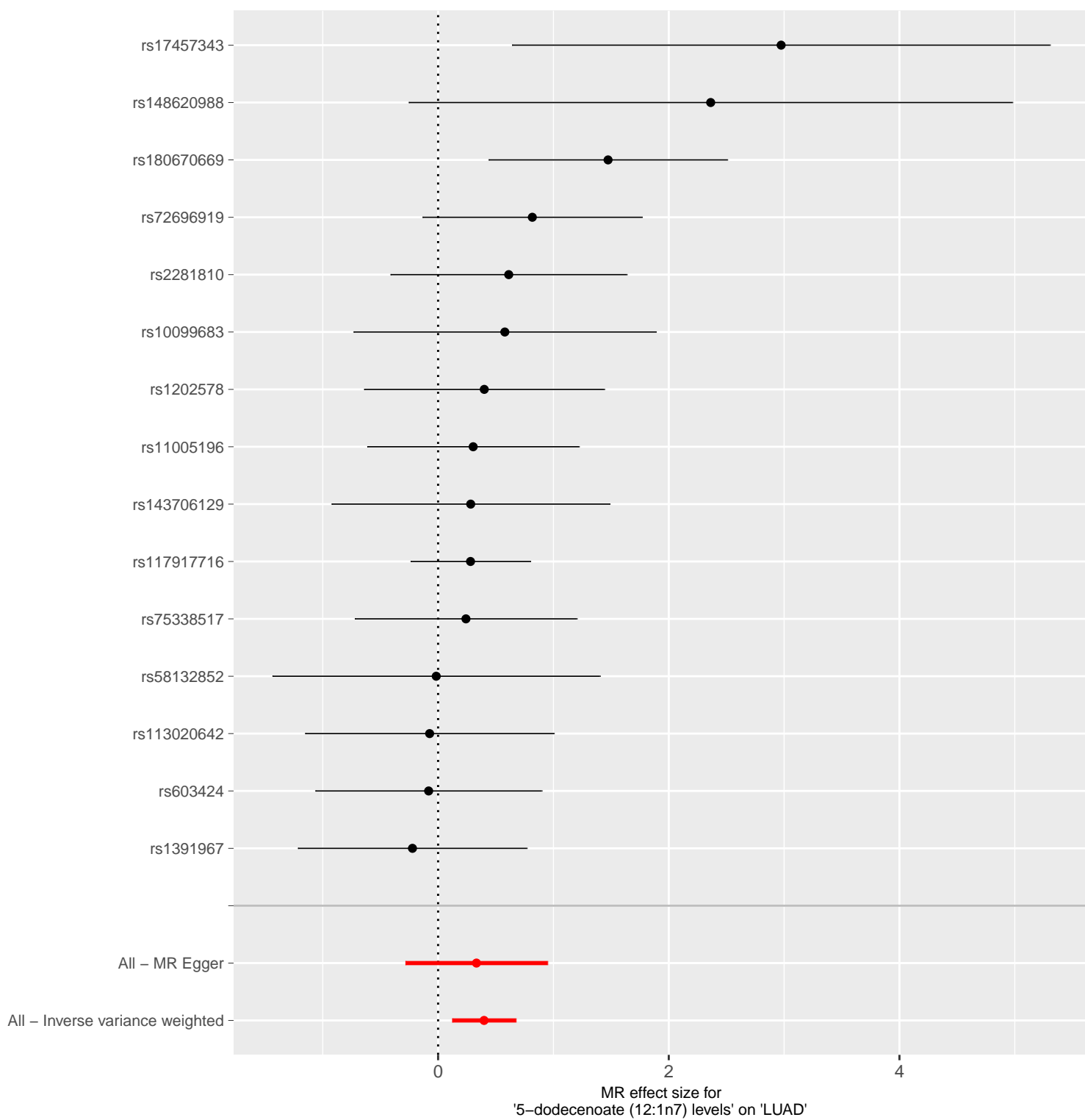

Supplement: Supplementary file 1 [file DataSheet1.zip › supplementary files/S2/GCST90199723/forest.pdf]

# MR Method

- Inverse variance weighted
- MR Egger

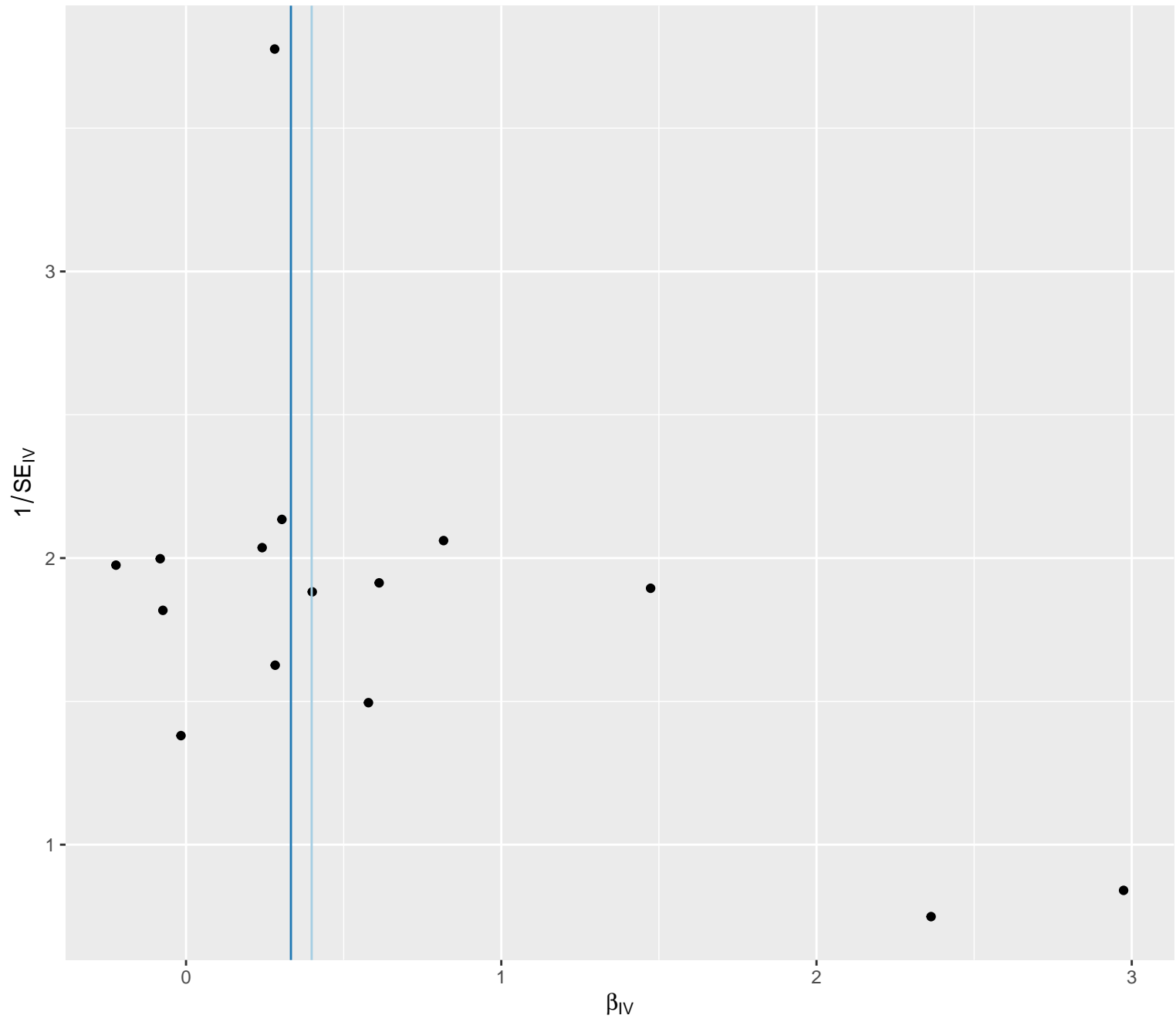

Supplement: Supplementary file 1 [file DataSheet1.zip › supplementary files/S2/GCST90199723/funnelplot.pdf]

# MR Test

- Inverse variance weighted
- MR Egger
- Simple mode
- Weighted median
- Weighted mode

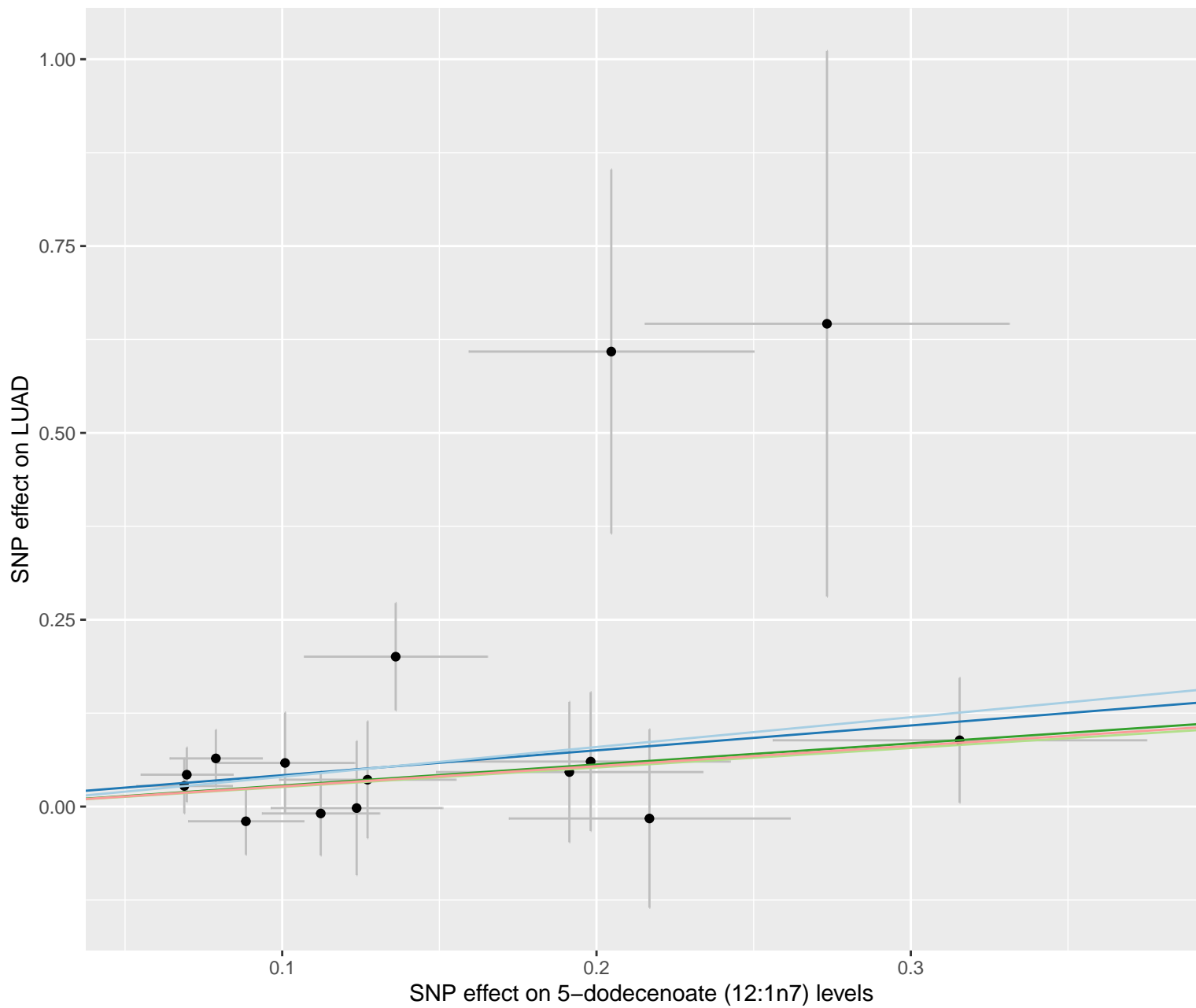

Supplement: Supplementary file 1 [file DataSheet1.zip › supplementary files/S2/GCST90199723/scatter.pdf]

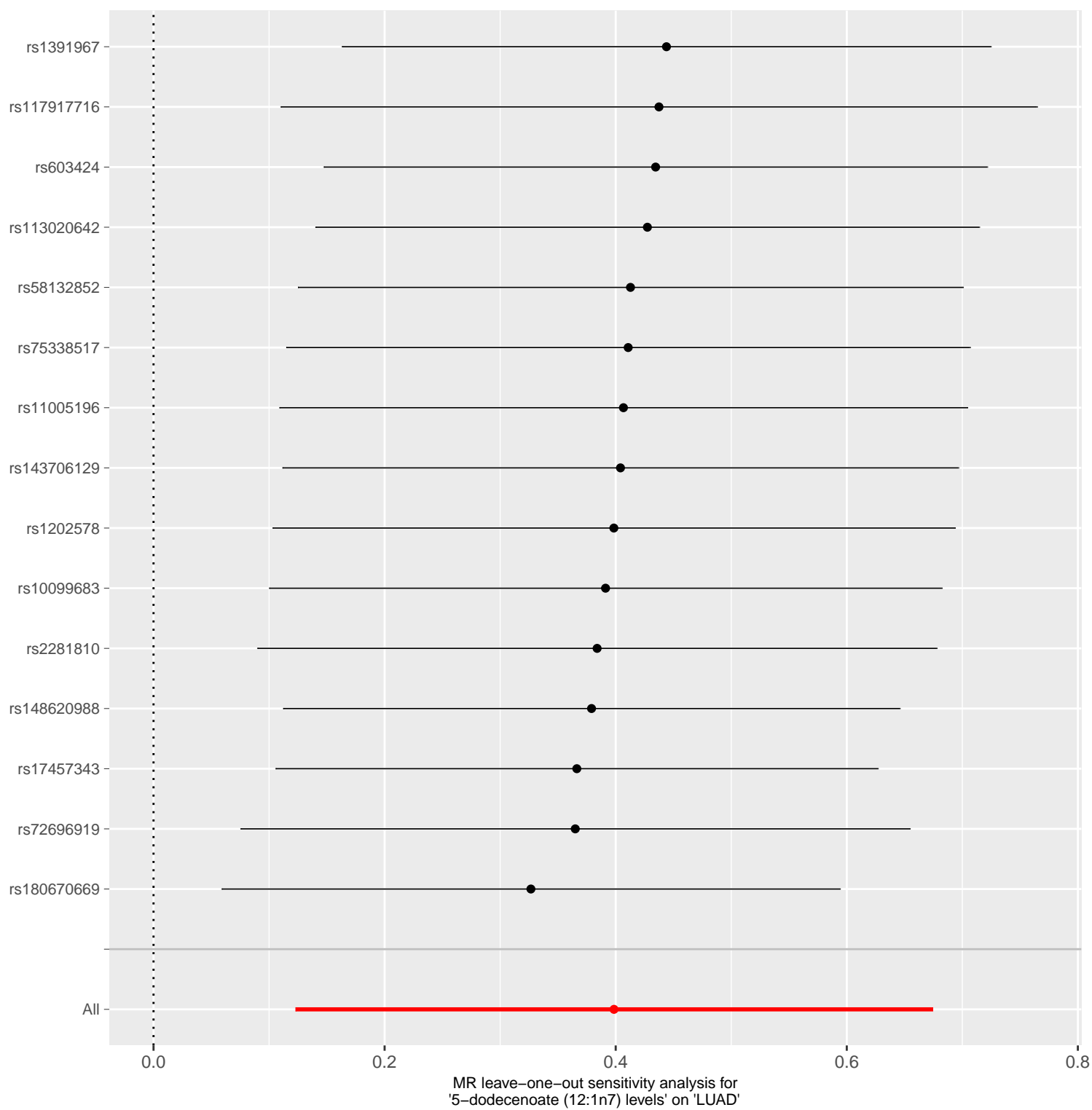

Supplement: Supplementary file 1 [file DataSheet1.zip › supplementary files/S2/GCST90199723/sensitivity-analysis.pdf]

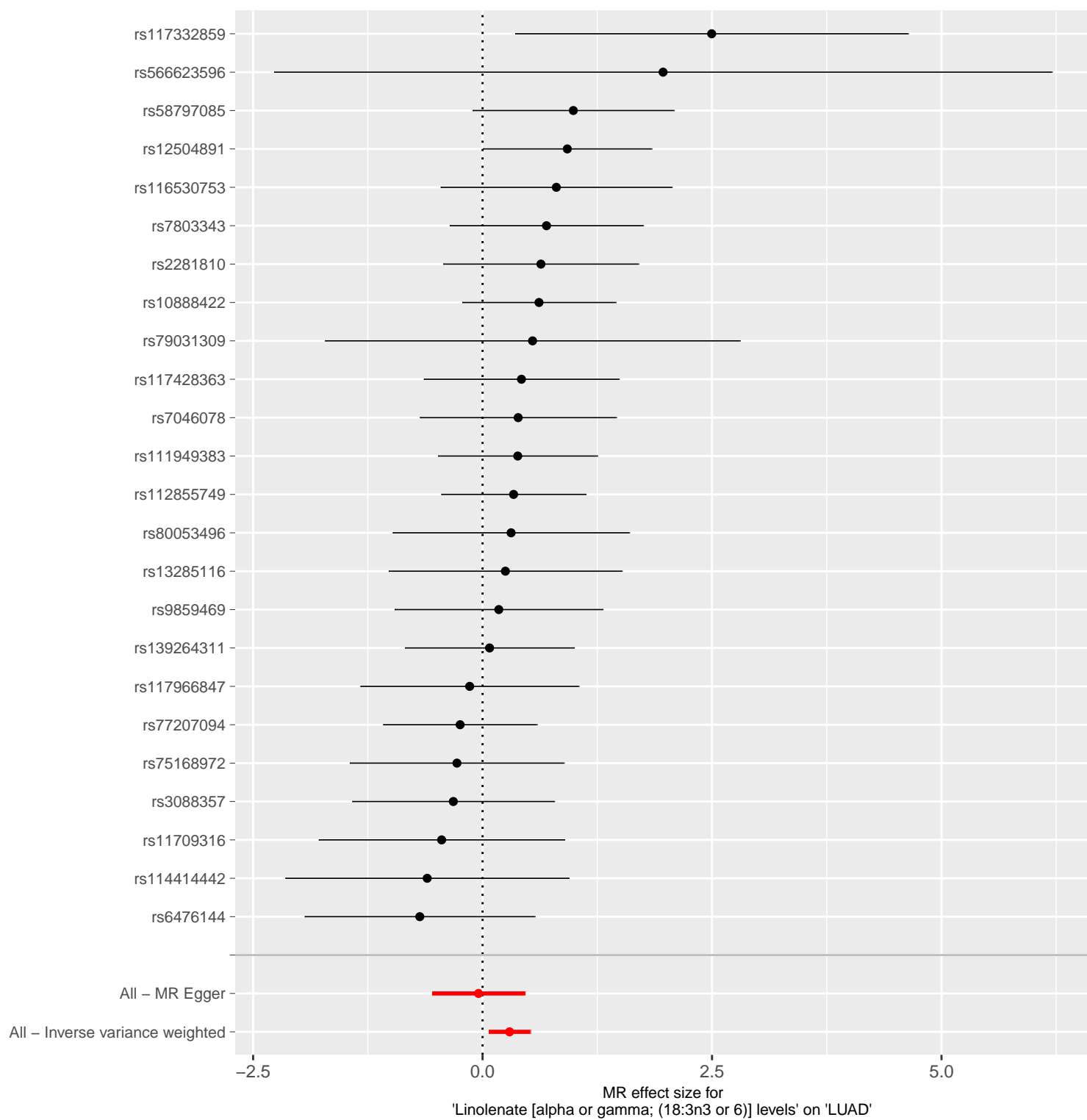

Supplement: Supplementary file 1 [file DataSheet1.zip › supplementary files/S2/GCST90199748/forest.pdf]

# MR Method

- Inverse variance weighted
- MR Egger

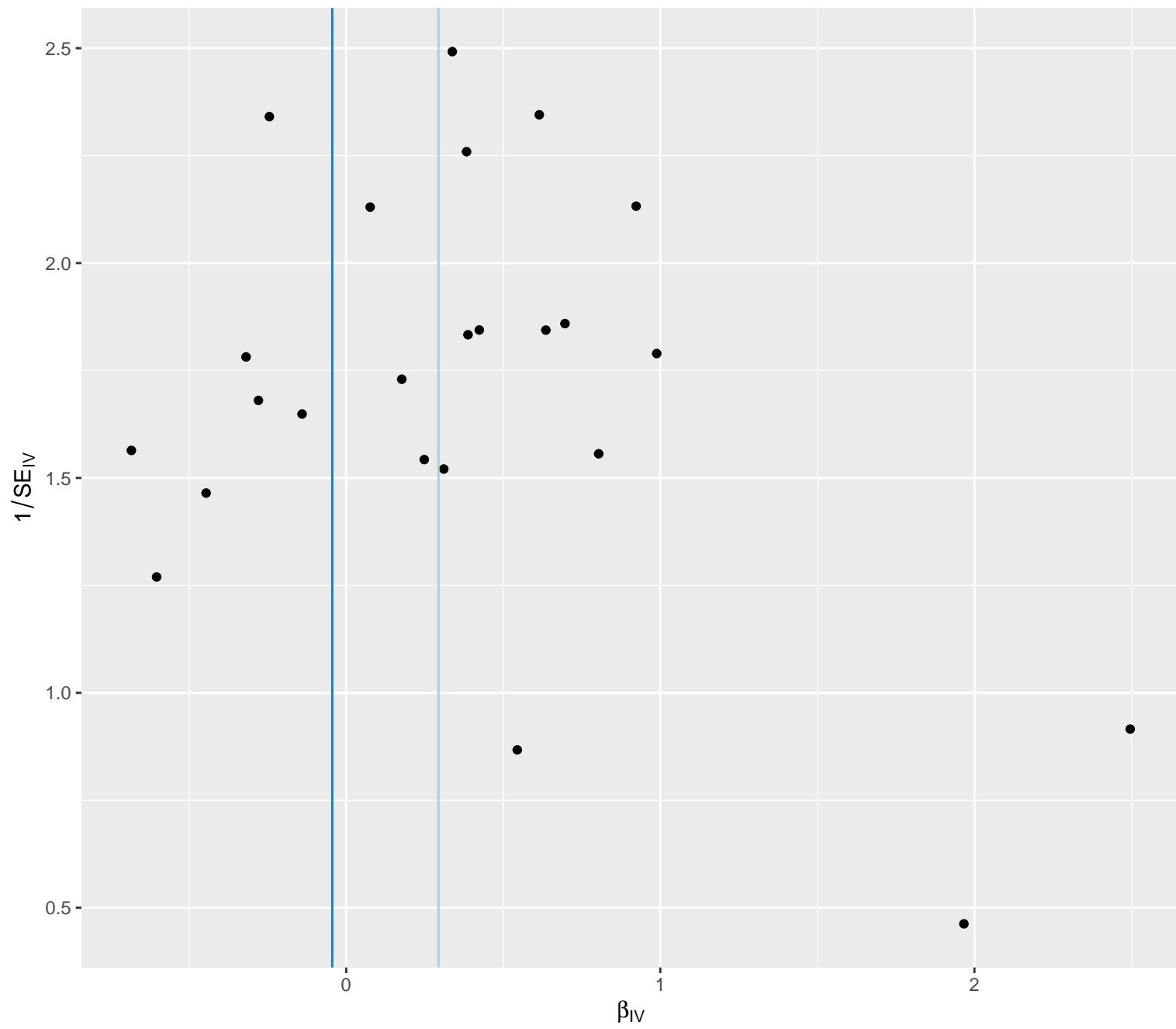

Supplement: Supplementary file 1 [file DataSheet1.zip › supplementary files/S2/GCST90199748/funnelplot.pdf]

# MR Test

- Inverse variance weighted
- MR Egger
- Simple mode
- Weighted median
- Weighted mode

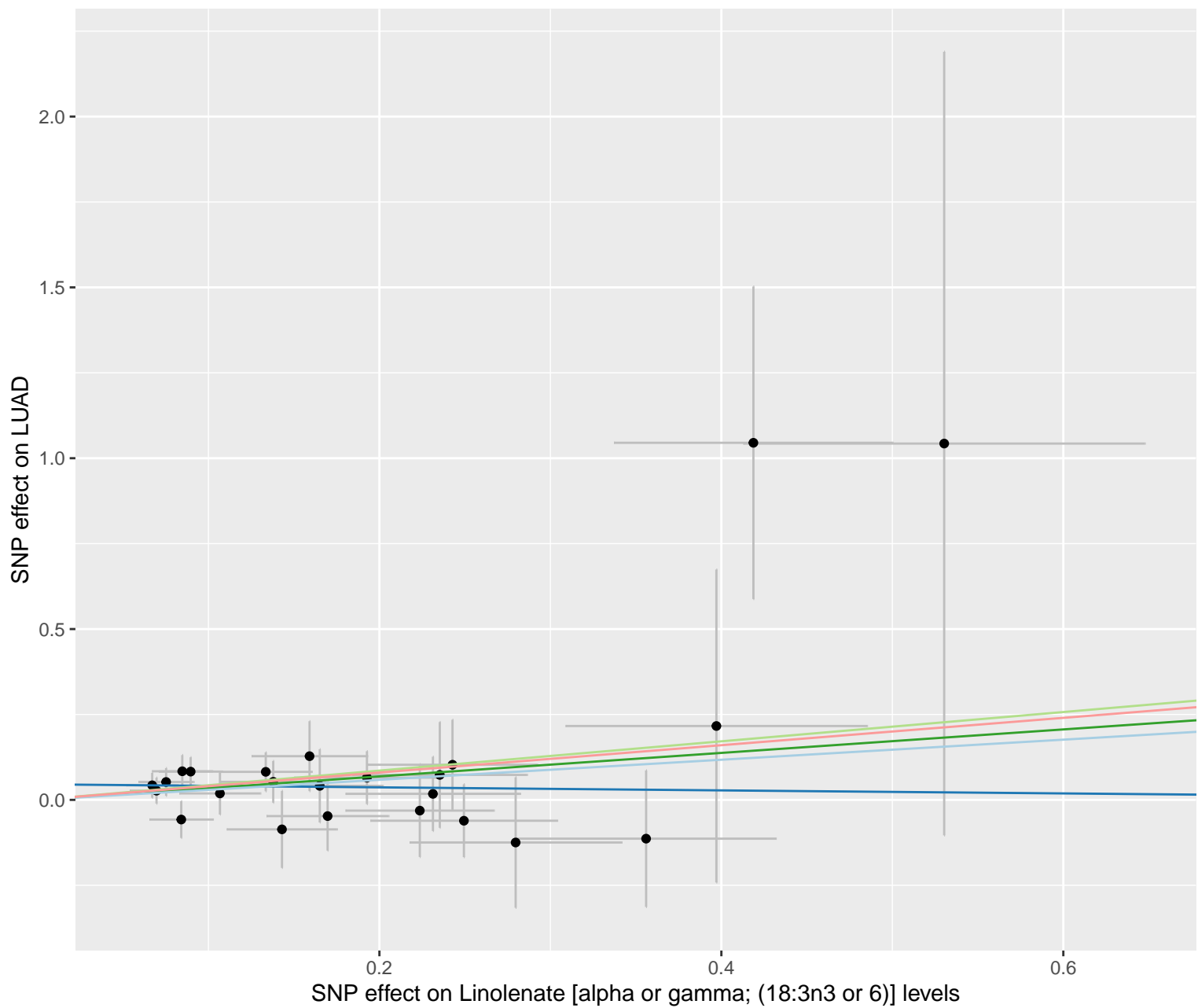

Supplement: Supplementary file 1 [file DataSheet1.zip › supplementary files/S2/GCST90199748/scatter.pdf]

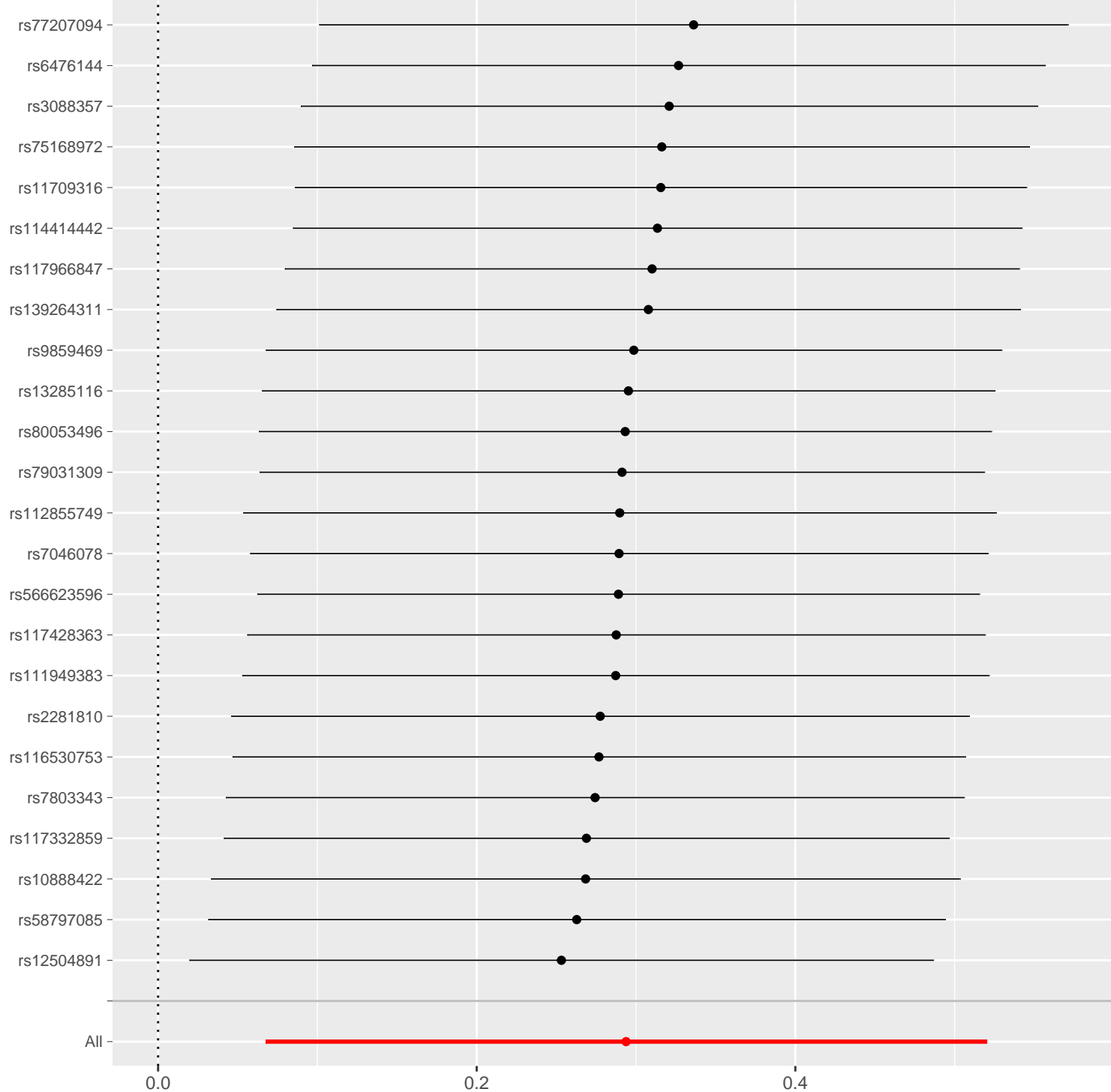

MR leave-one-out sensitivity analysis for  
'Linolenate [alpha or gamma; (18:3n3 or 6)] levels' on 'LUAD'

Supplement: Supplementary file 1 [file DataSheet1.zip › supplementary files/S2/GCST90199748/sensitivity-analysis.pdf]

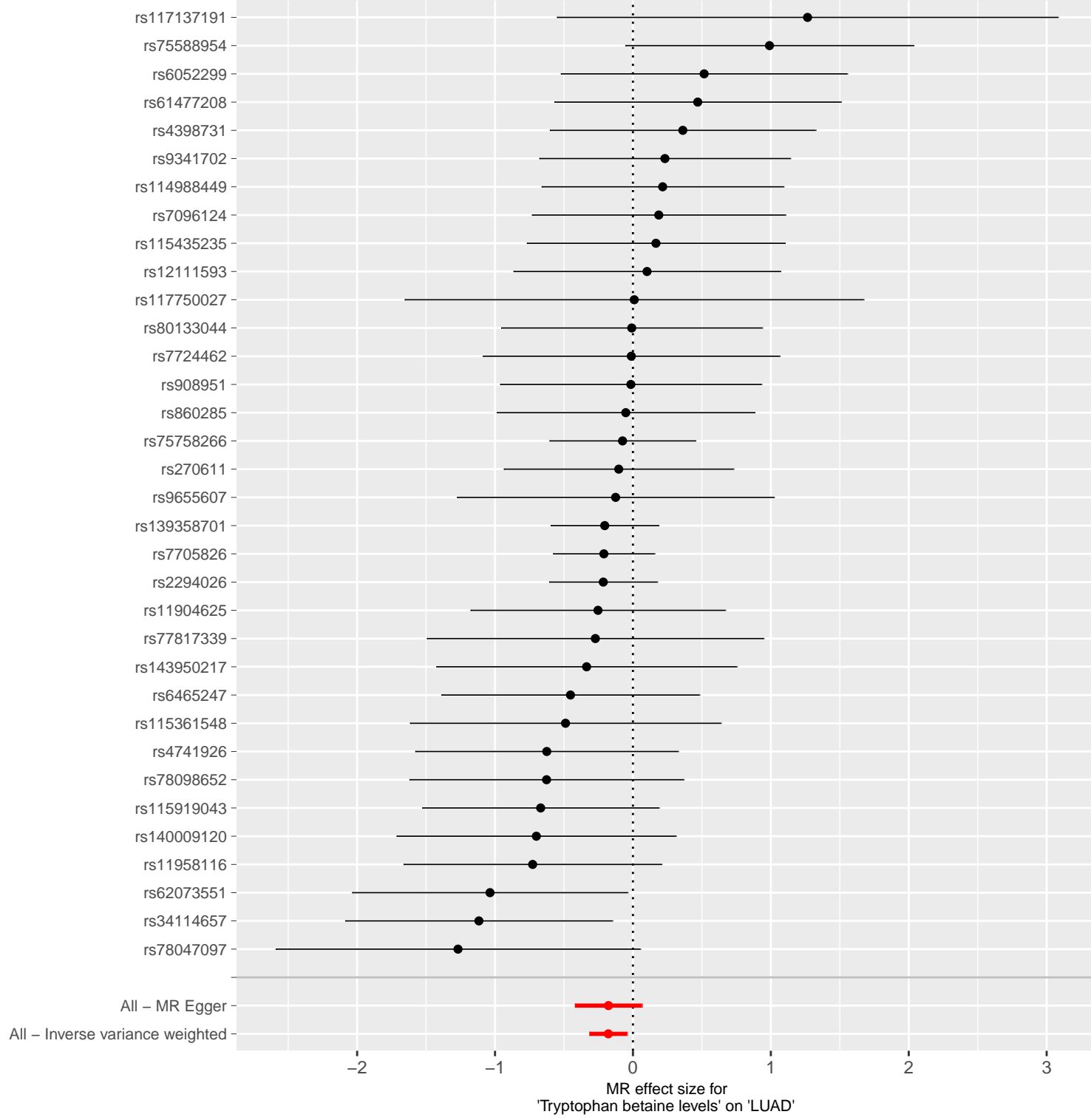

Supplement: Supplementary file 1 [file DataSheet1.zip › supplementary files/S2/GCST90199819/forest.pdf]

# MR Method

- Inverse variance weighted
- MR Egger

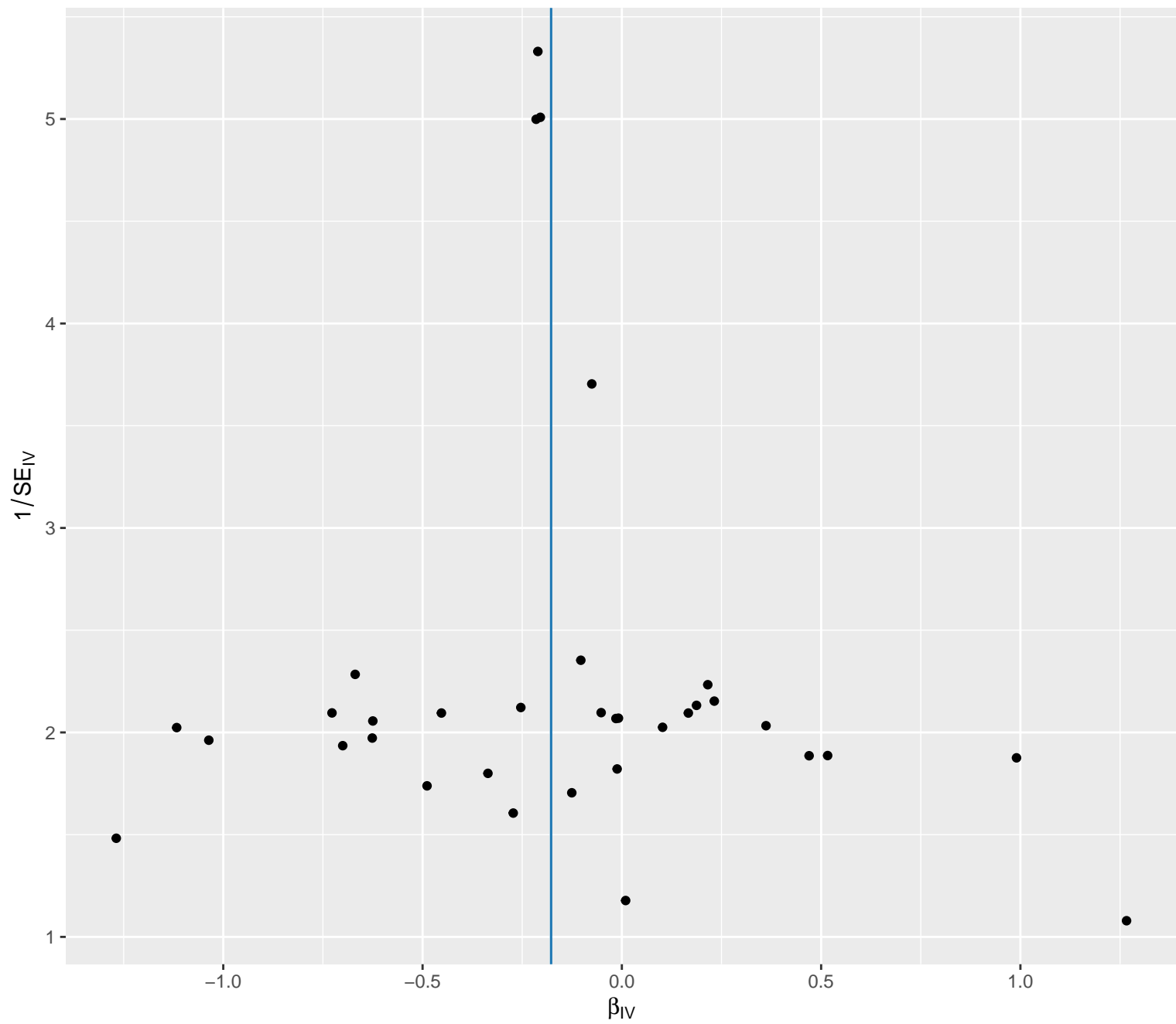

Supplement: Supplementary file 1 [file DataSheet1.zip › supplementary files/S2/GCST90199819/funnelplot.pdf]

## MR Test

- Inverse variance weighted
- MR Egger
- Simple mode

- Weighted median
- Weighted mode

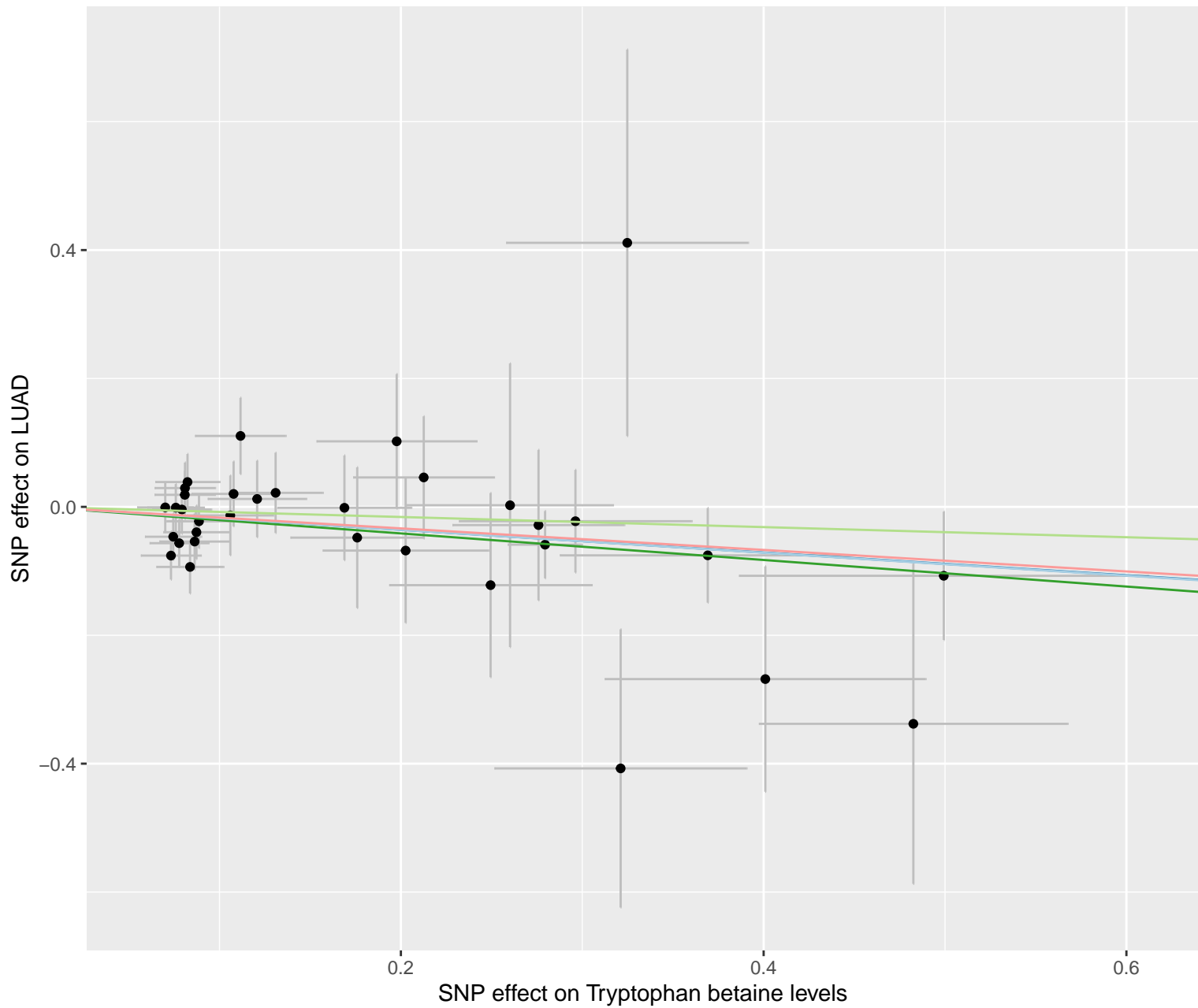

Supplement: Supplementary file 1 [file DataSheet1.zip › supplementary files/S2/GCST90199819/scatter.pdf]

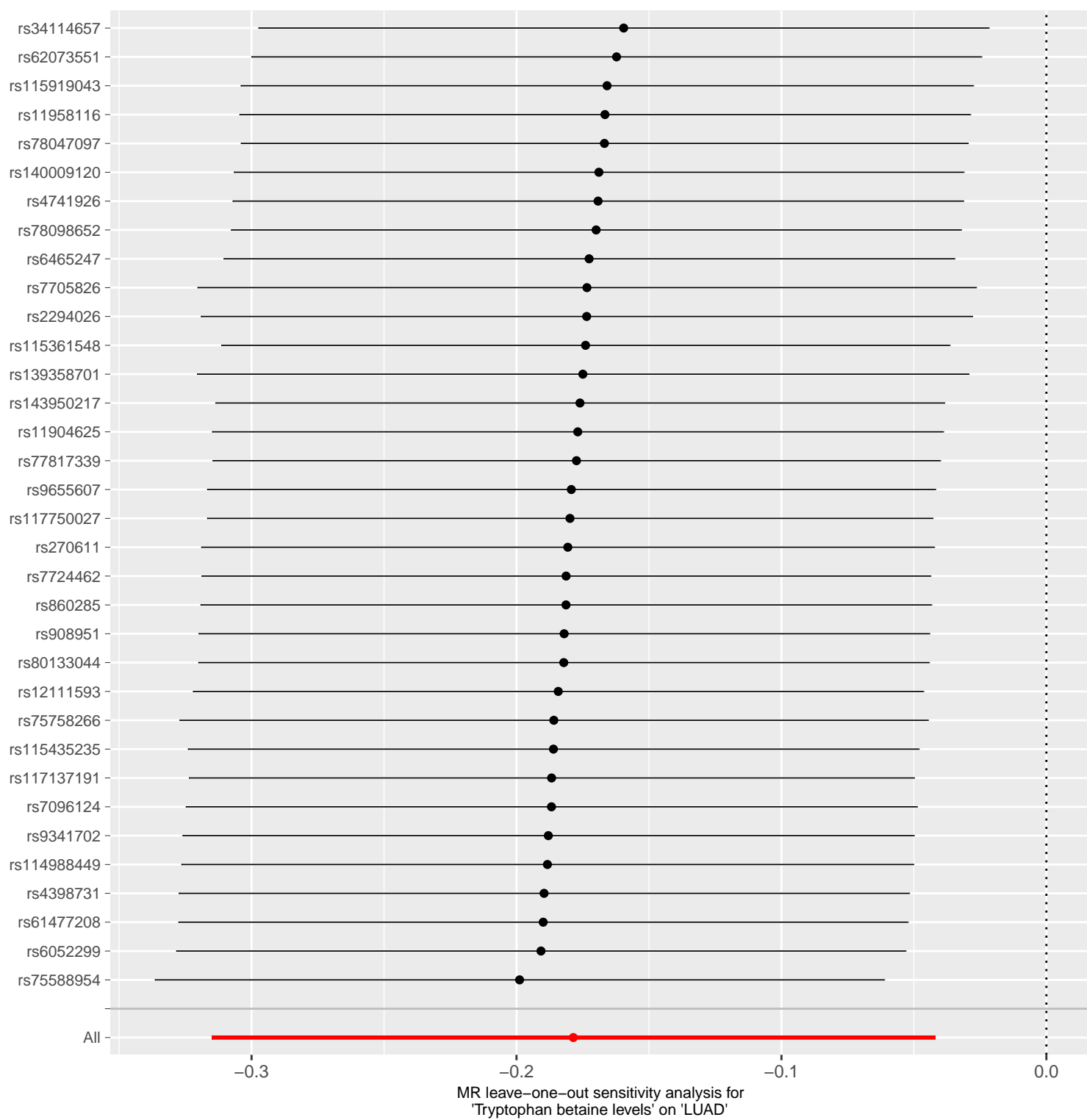

Supplement: Supplementary file 1 [file DataSheet1.zip › supplementary files/S2/GCST90199819/sensitivity-analysis.pdf]

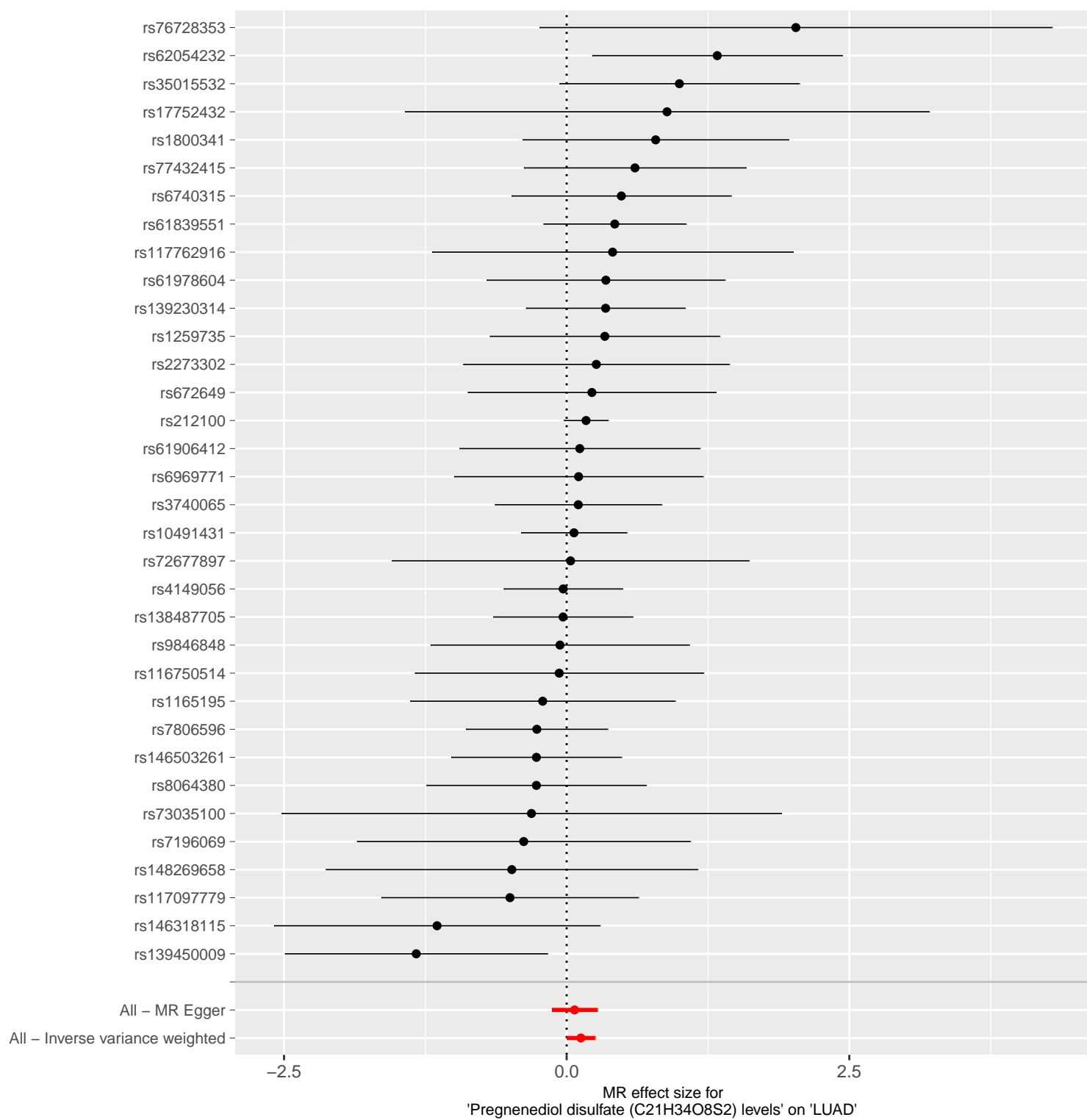

Supplement: Supplementary file 1 [file DataSheet1.zip › supplementary files/S2/GCST90199840/forest.pdf]

# MR Method

Inverse variance weighted  
MR Egger

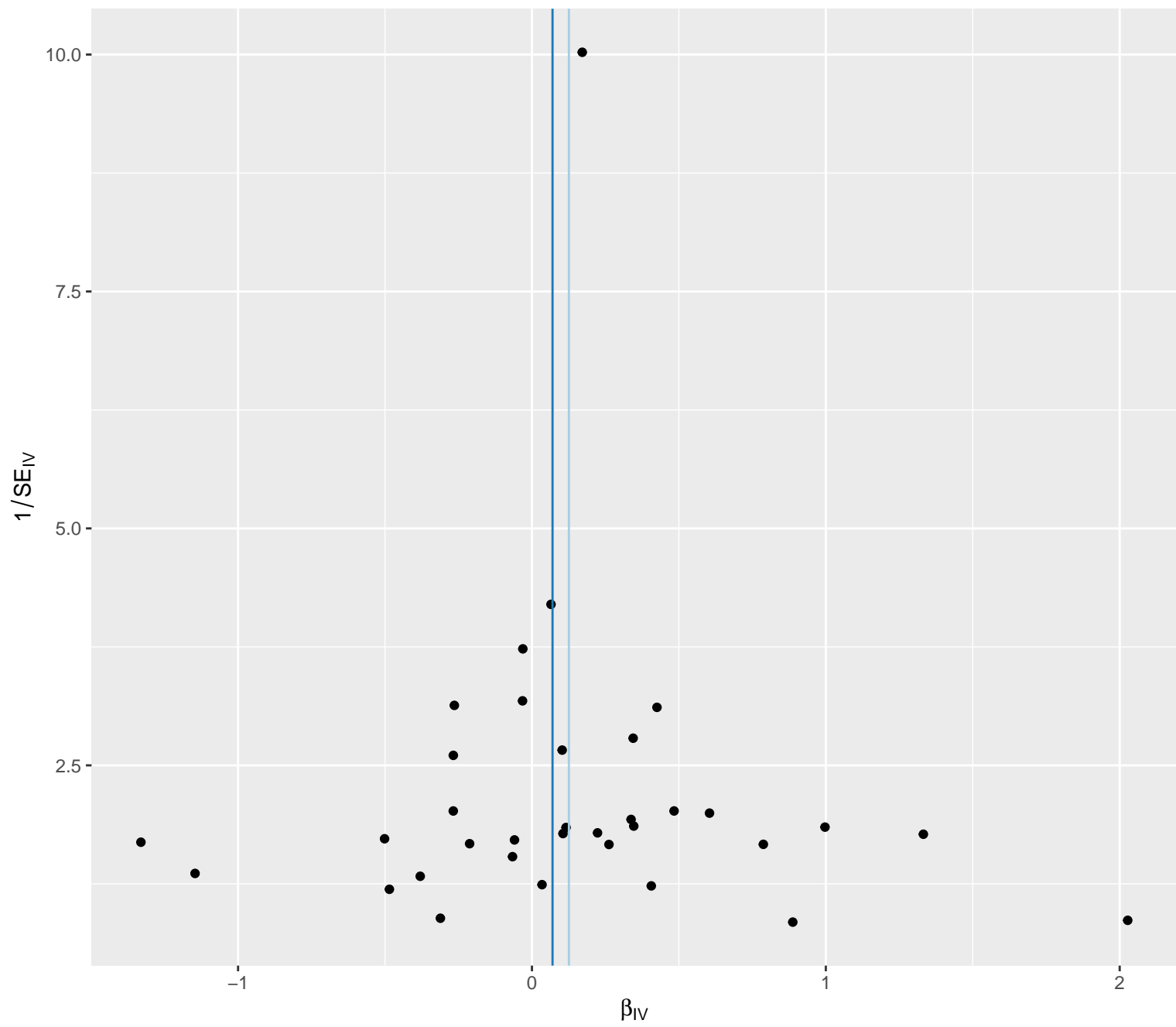

Supplement: Supplementary file 1 [file DataSheet1.zip › supplementary files/S2/GCST90199840/funnelplot.pdf]

# MR Test

- Inverse variance weighted
- MR Egger
- Simple mode
- Weighted median
- Weighted mode

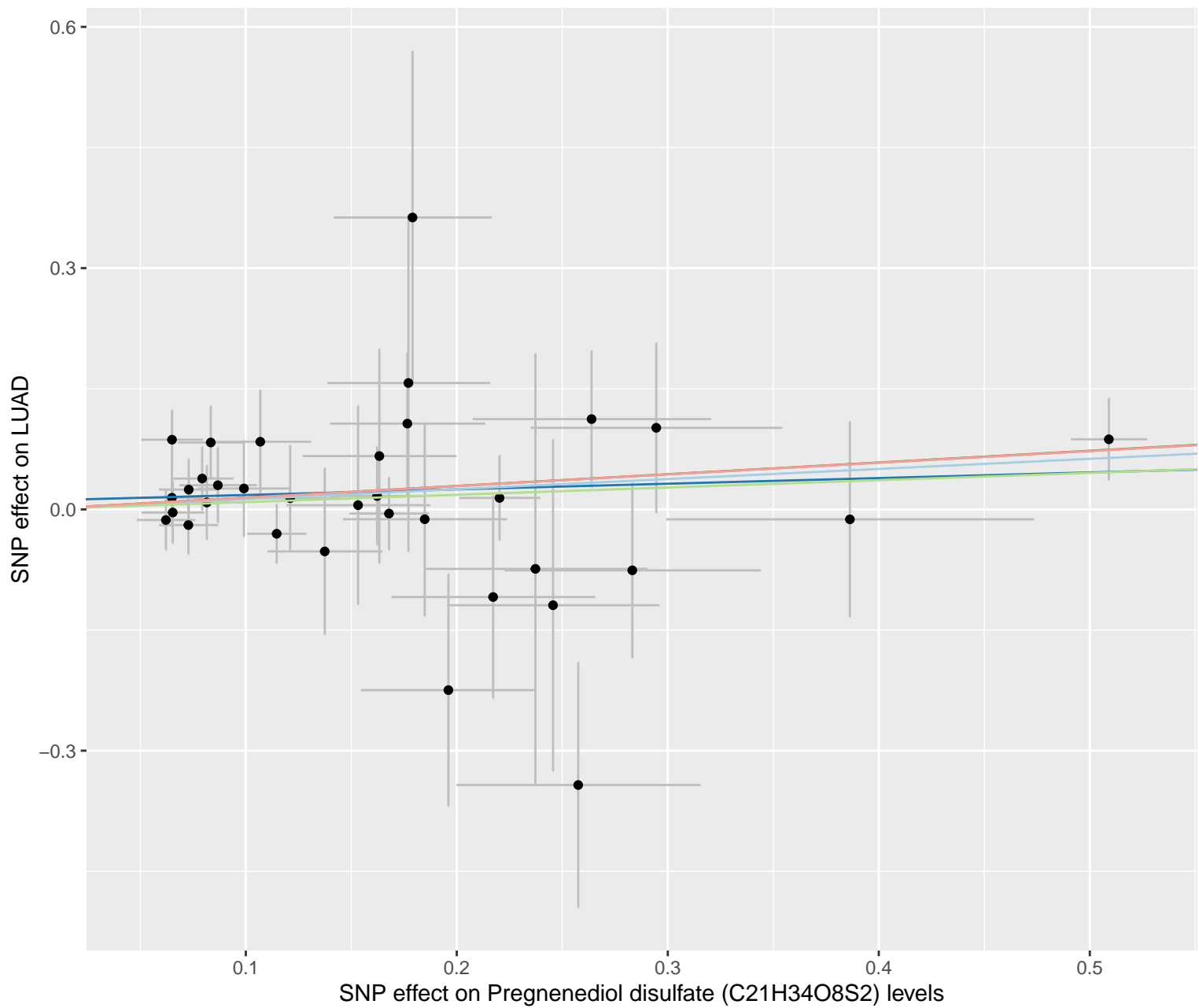

Supplement: Supplementary file 1 [file DataSheet1.zip › supplementary files/S2/GCST90199840/scatter.pdf]

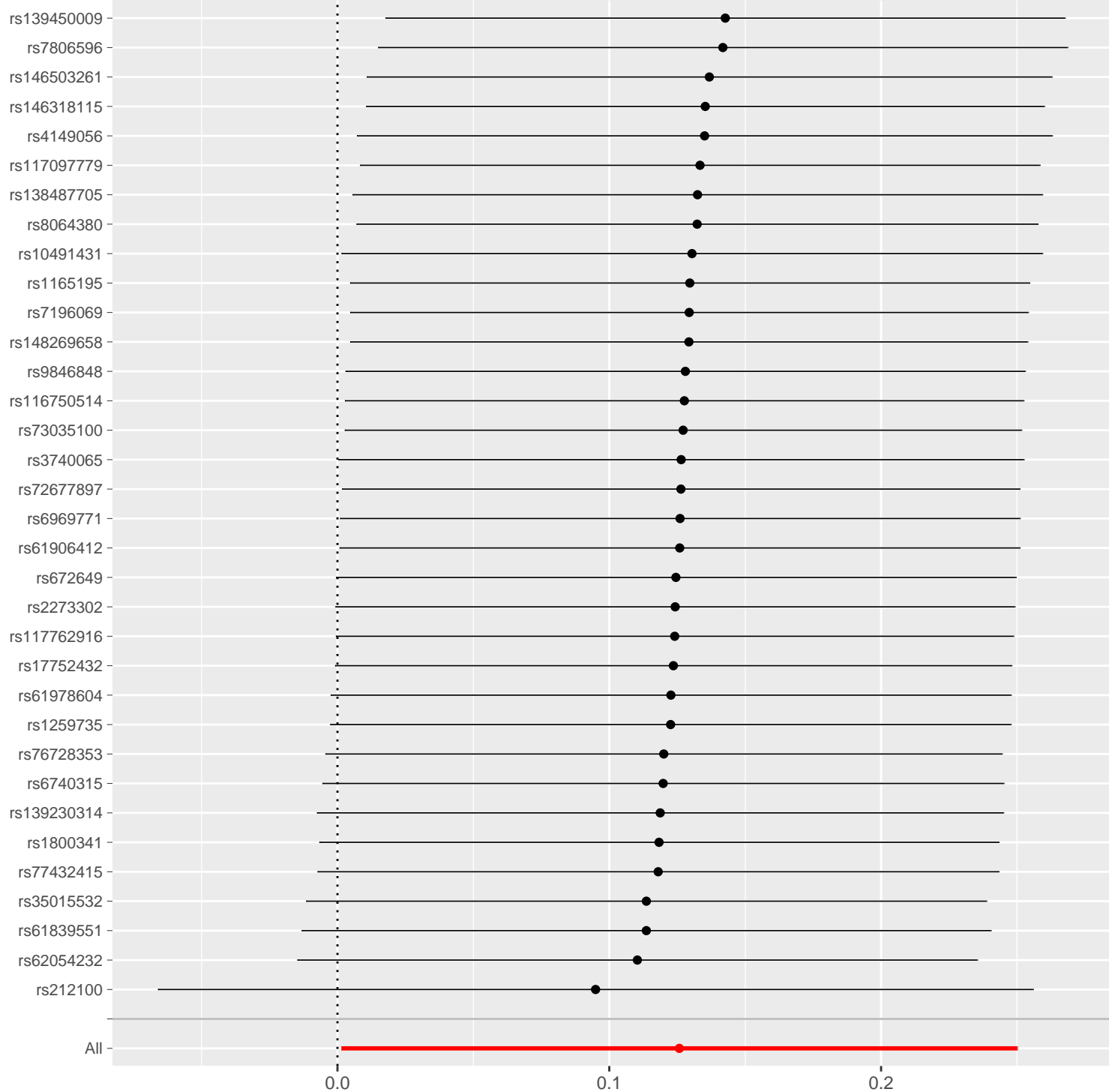

Supplement: Supplementary file 1 [file DataSheet1.zip › supplementary files/S2/GCST90199840/sensitivity-analysis.pdf]

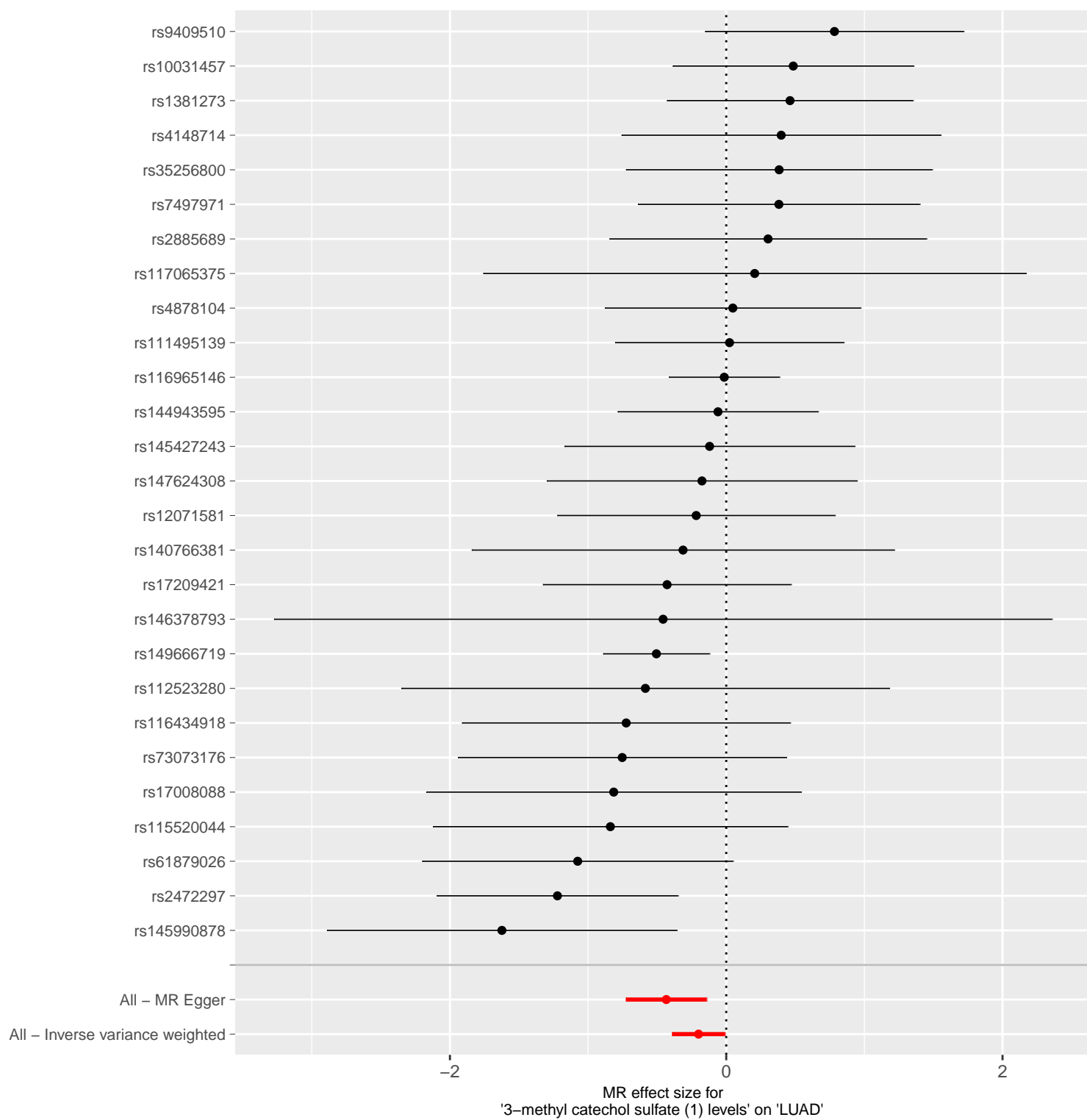

Supplement: Supplementary file 1 [file DataSheet1.zip › supplementary files/S2/GCST90199938/forest.pdf]

# MR Method

- Inverse variance weighted
- MR Egger

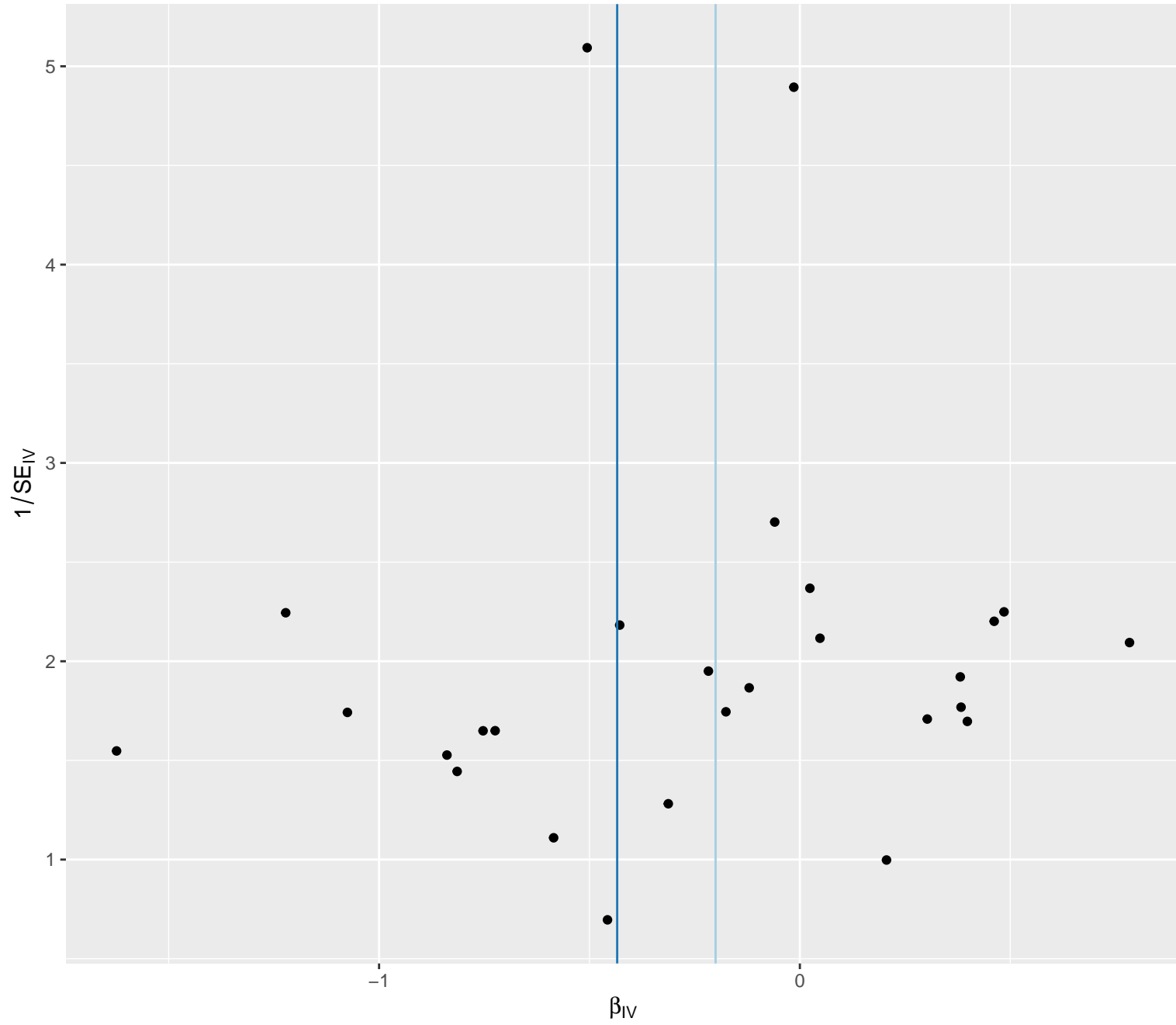

Supplement: Supplementary file 1 [file DataSheet1.zip › supplementary files/S2/GCST90199938/funnelplot.pdf]

# MR Test

- Inverse variance weighted
- MR Egger
- Simple mode
- Weighted median
- Weighted mode

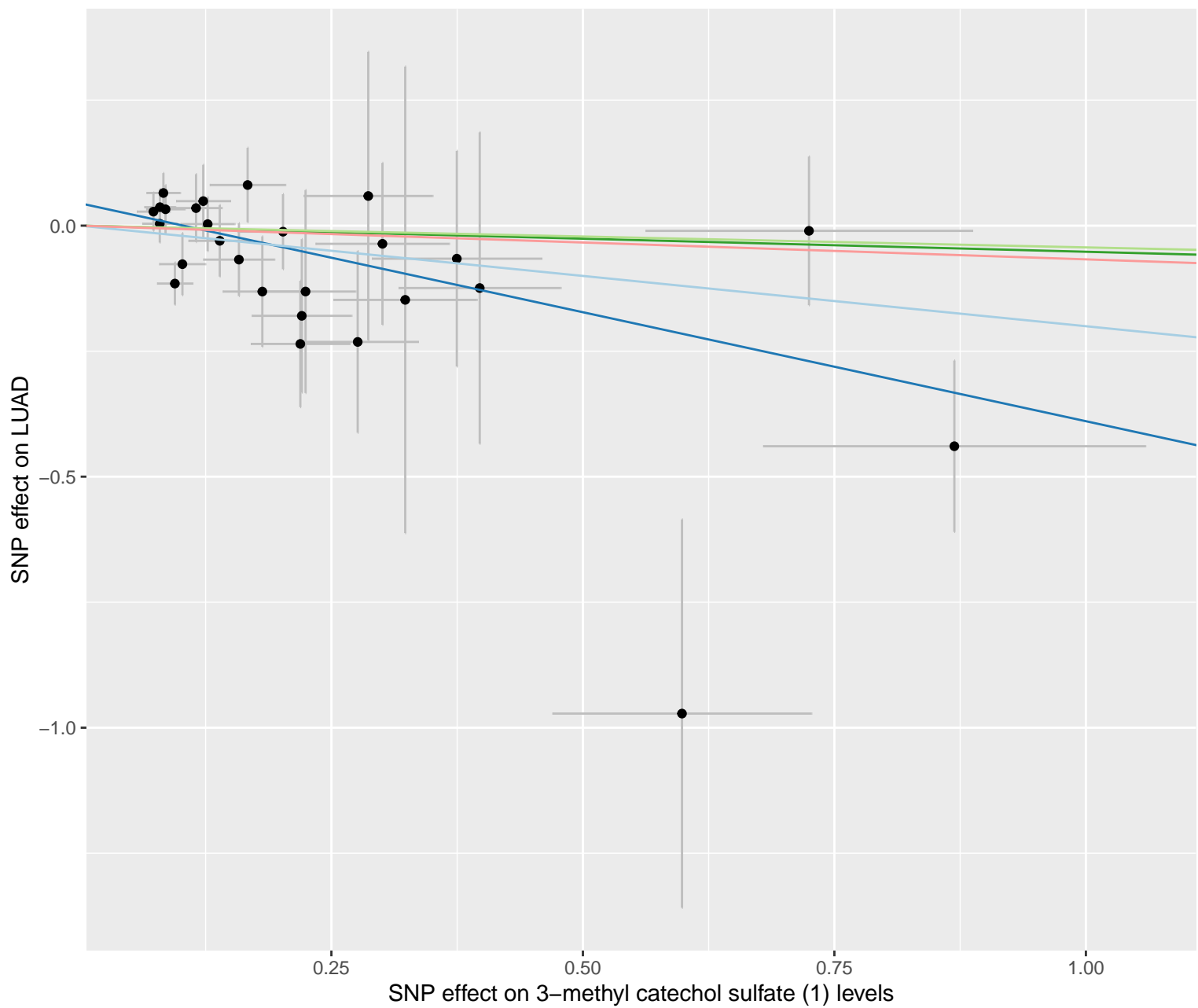

Supplement: Supplementary file 1 [file DataSheet1.zip › supplementary files/S2/GCST90199938/scatter.pdf]

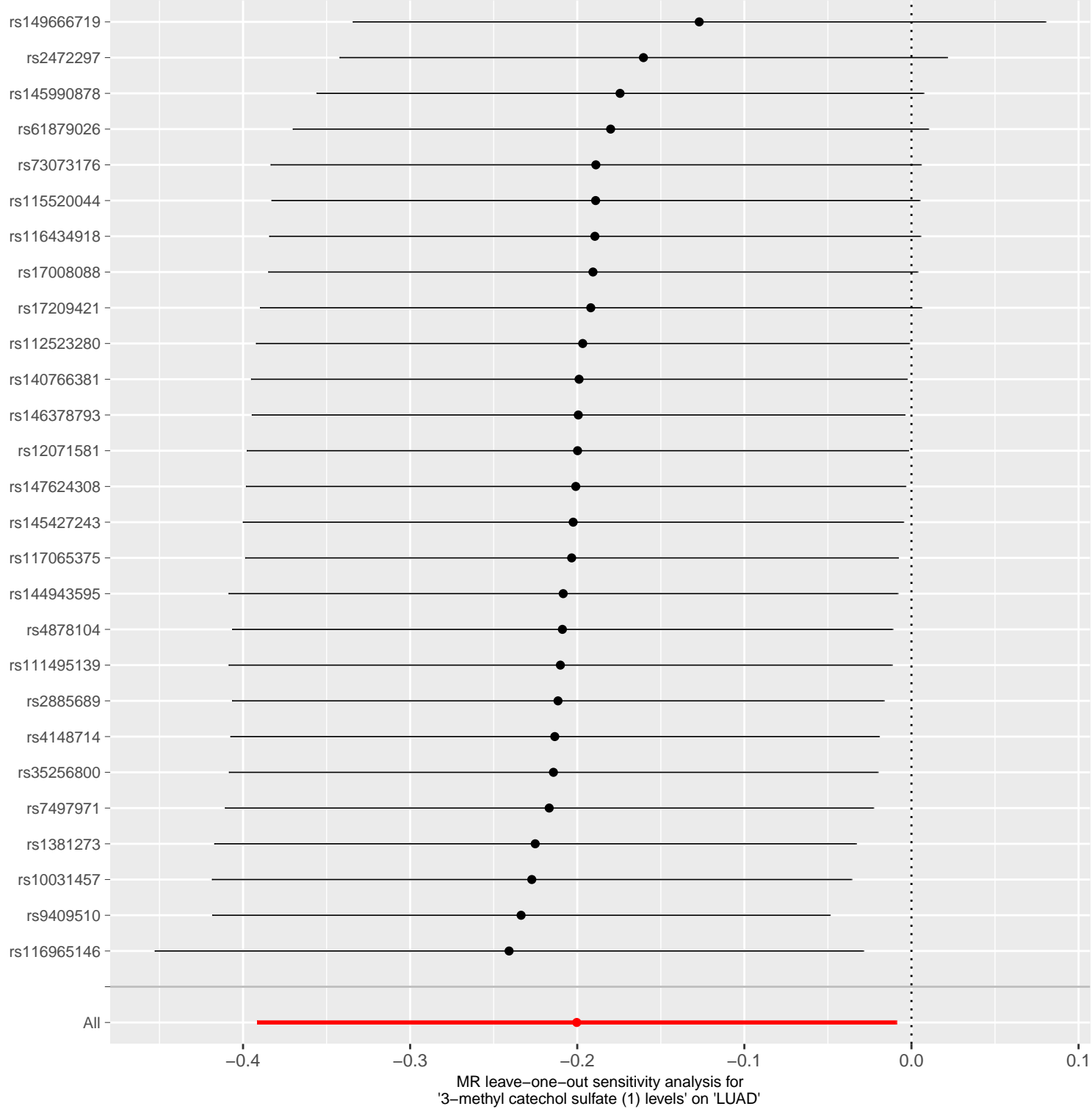

Supplement: Supplementary file 1 [file DataSheet1.zip › supplementary files/S2/GCST90199938/sensitivity-analysis.pdf]

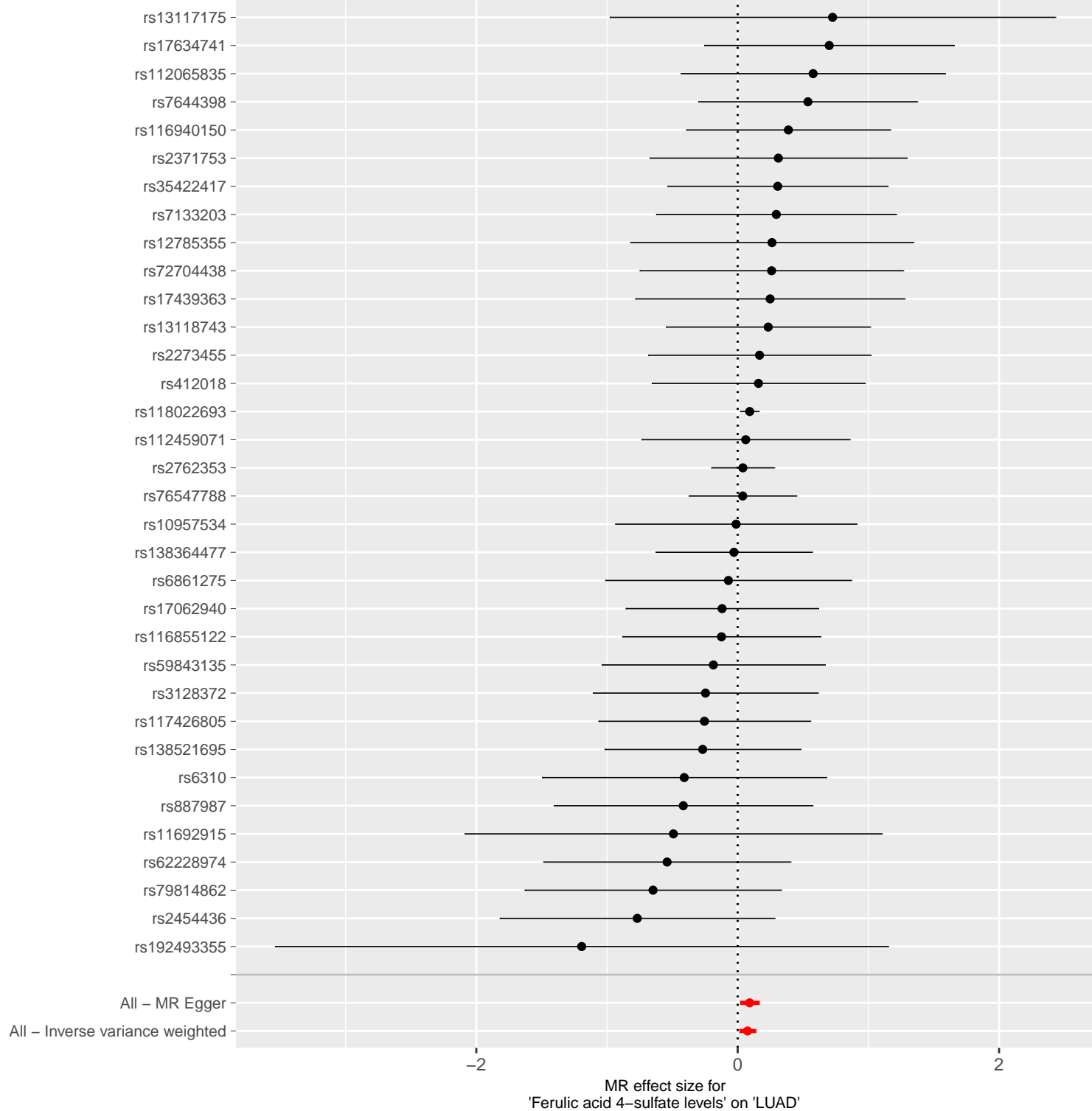

Supplement: Supplementary file 1 [file DataSheet1.zip › supplementary files/S2/GCST90199955/forest.pdf]

# MR Method

- Inverse variance weighted
- MR Egger

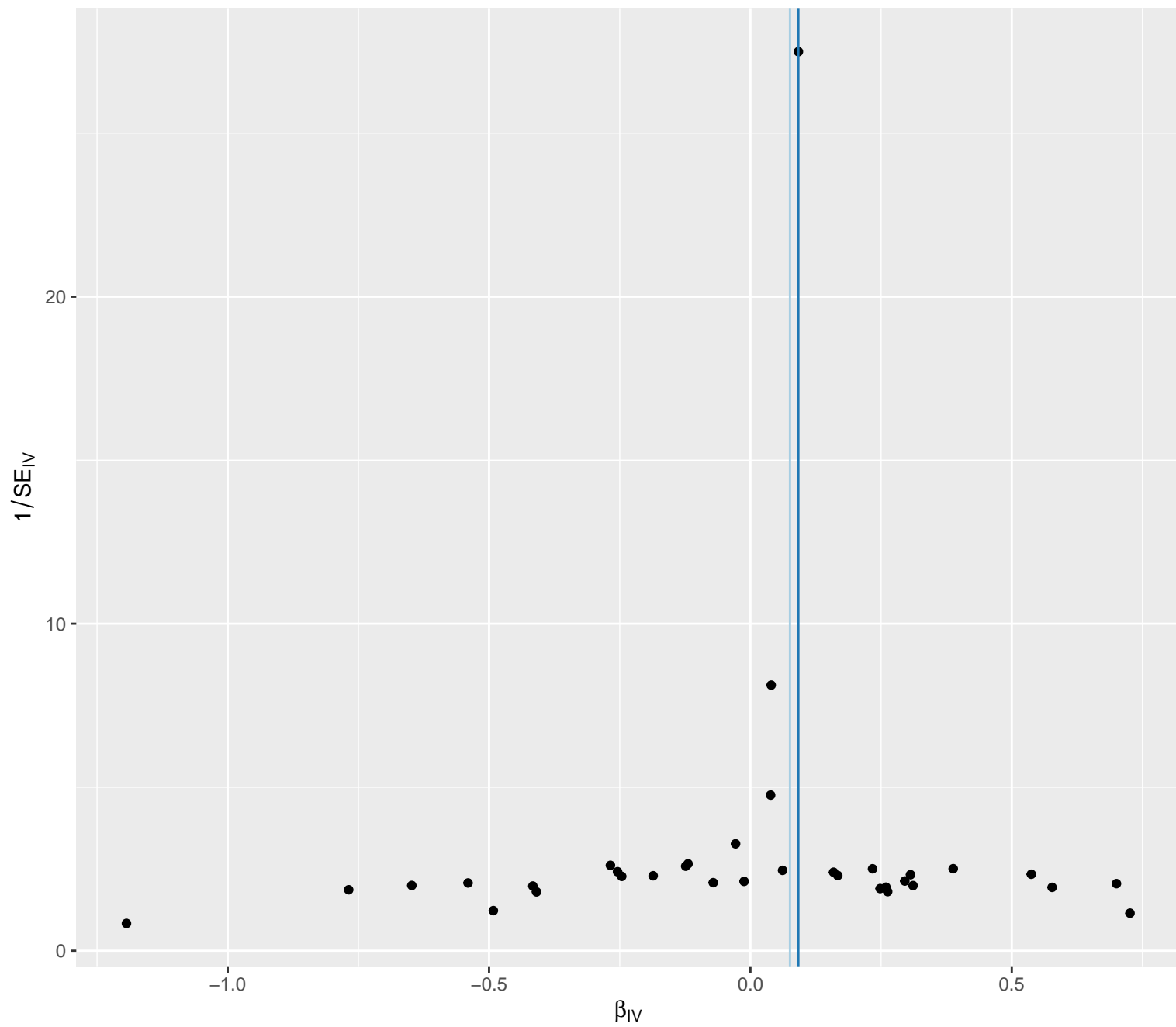

Supplement: Supplementary file 1 [file DataSheet1.zip › supplementary files/S2/GCST90199955/funnelplot.pdf]

# MR Test

- Inverse variance weighted
- MR Egger
- Simple mode
- Weighted median
- Weighted mode

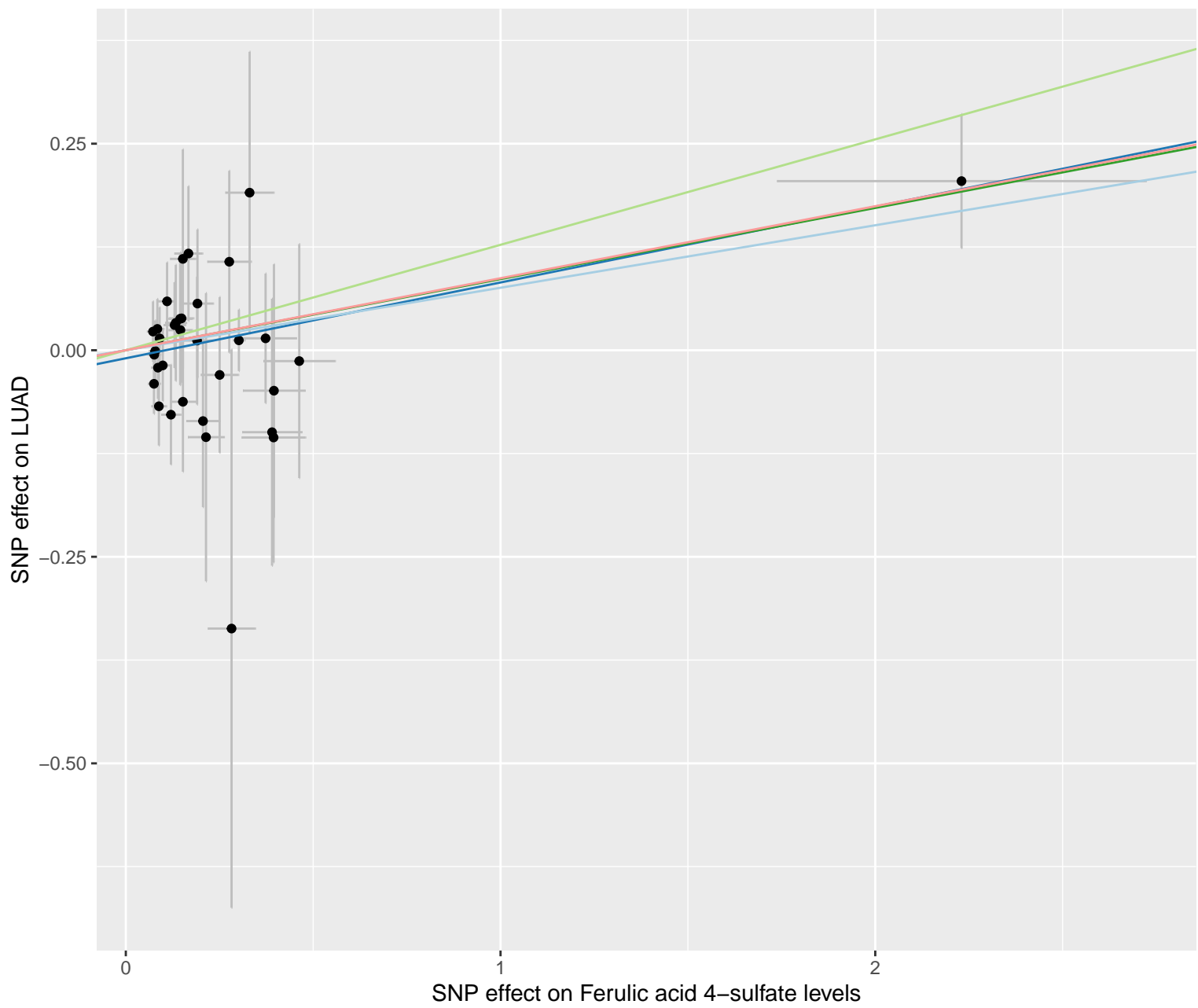

Supplement: Supplementary file 1 [file DataSheet1.zip › supplementary files/S2/GCST90199955/scatter.pdf]

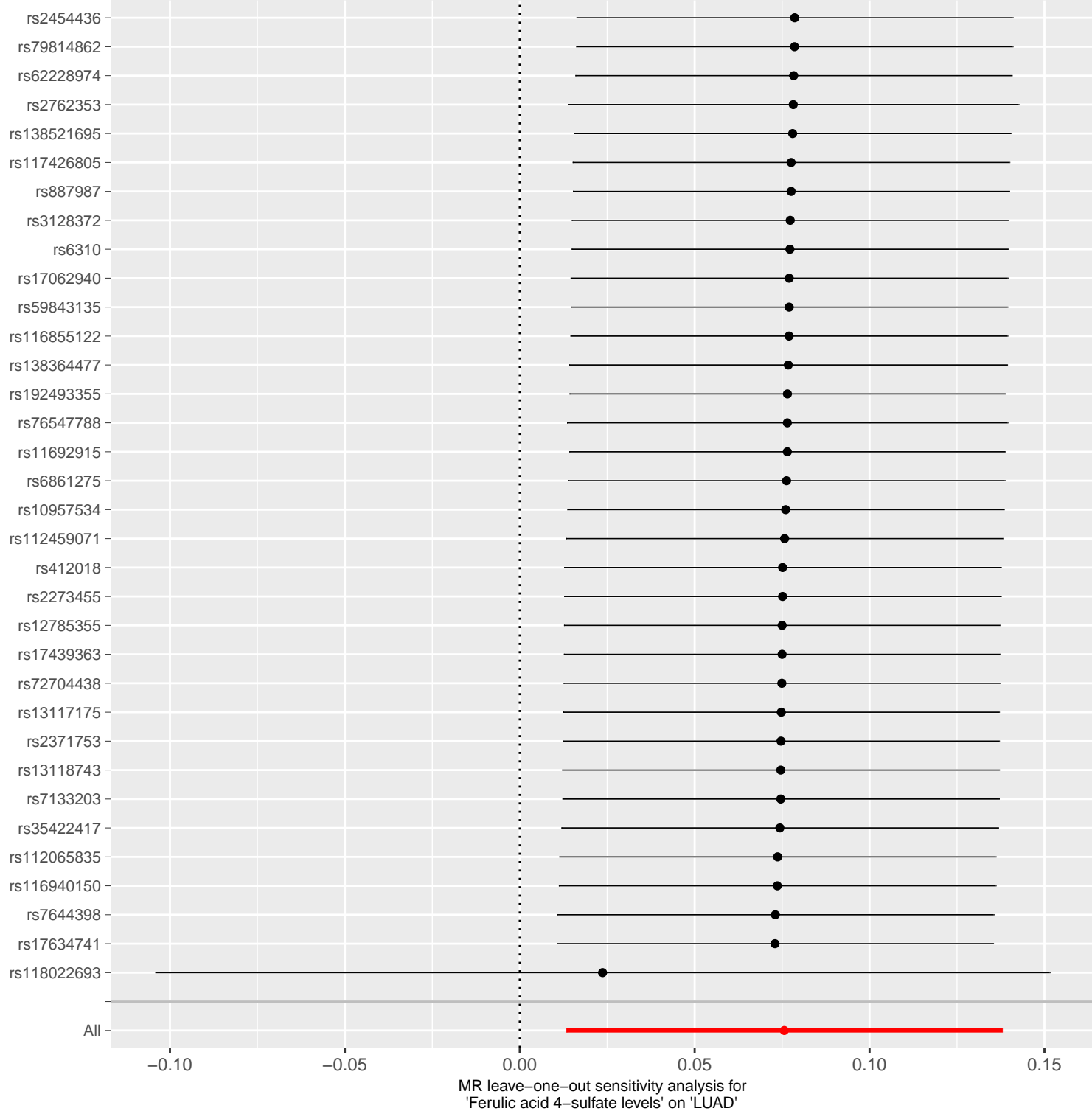

Supplement: Supplementary file 1 [file DataSheet1.zip › supplementary files/S2/GCST90199955/sensitivity-analysis.pdf]

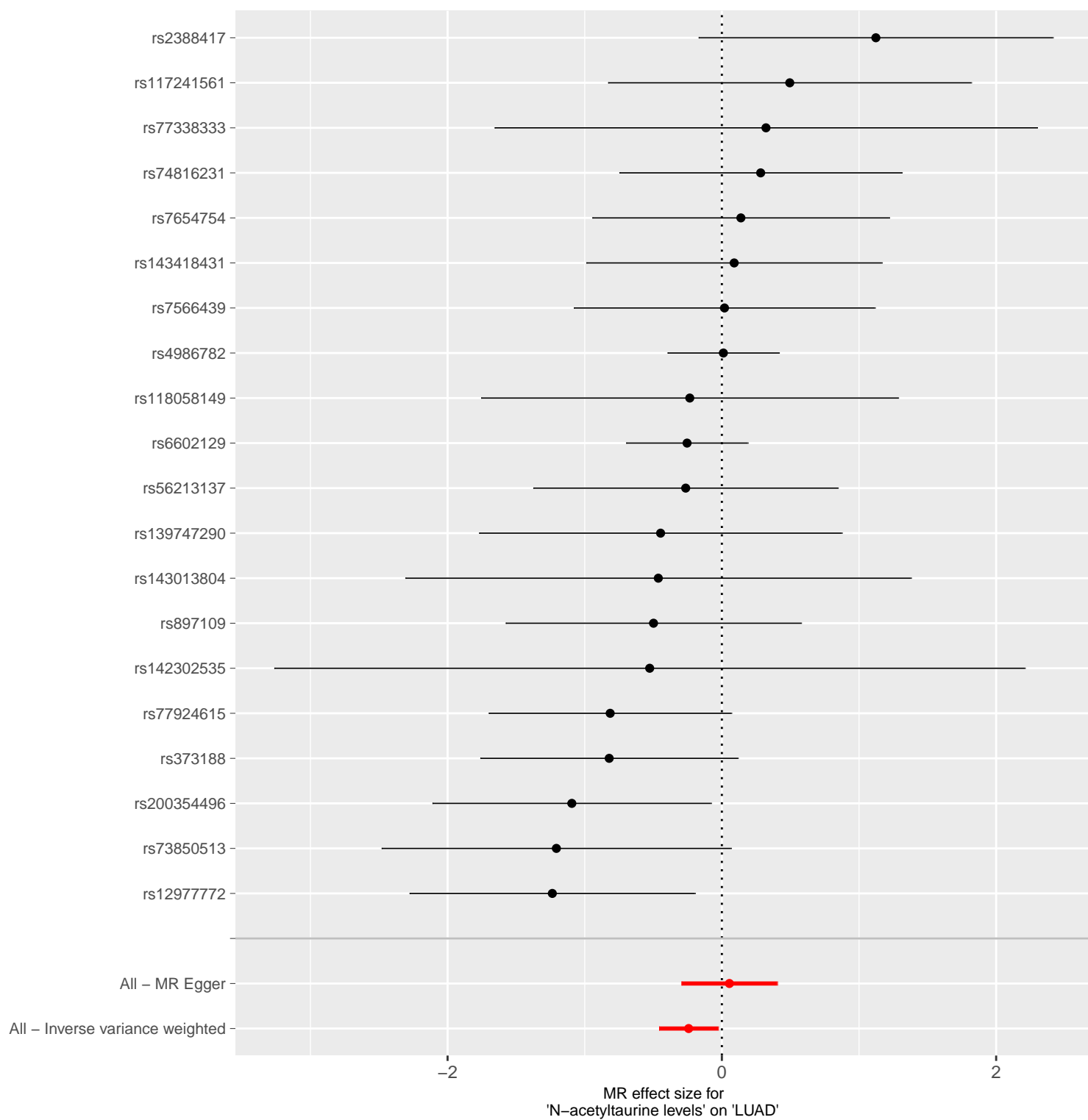

Supplement: Supplementary file 1 [file DataSheet1.zip › supplementary files/S2/GCST90199967/forest.pdf]

# MR Method

- Inverse variance weighted
- MR Egger

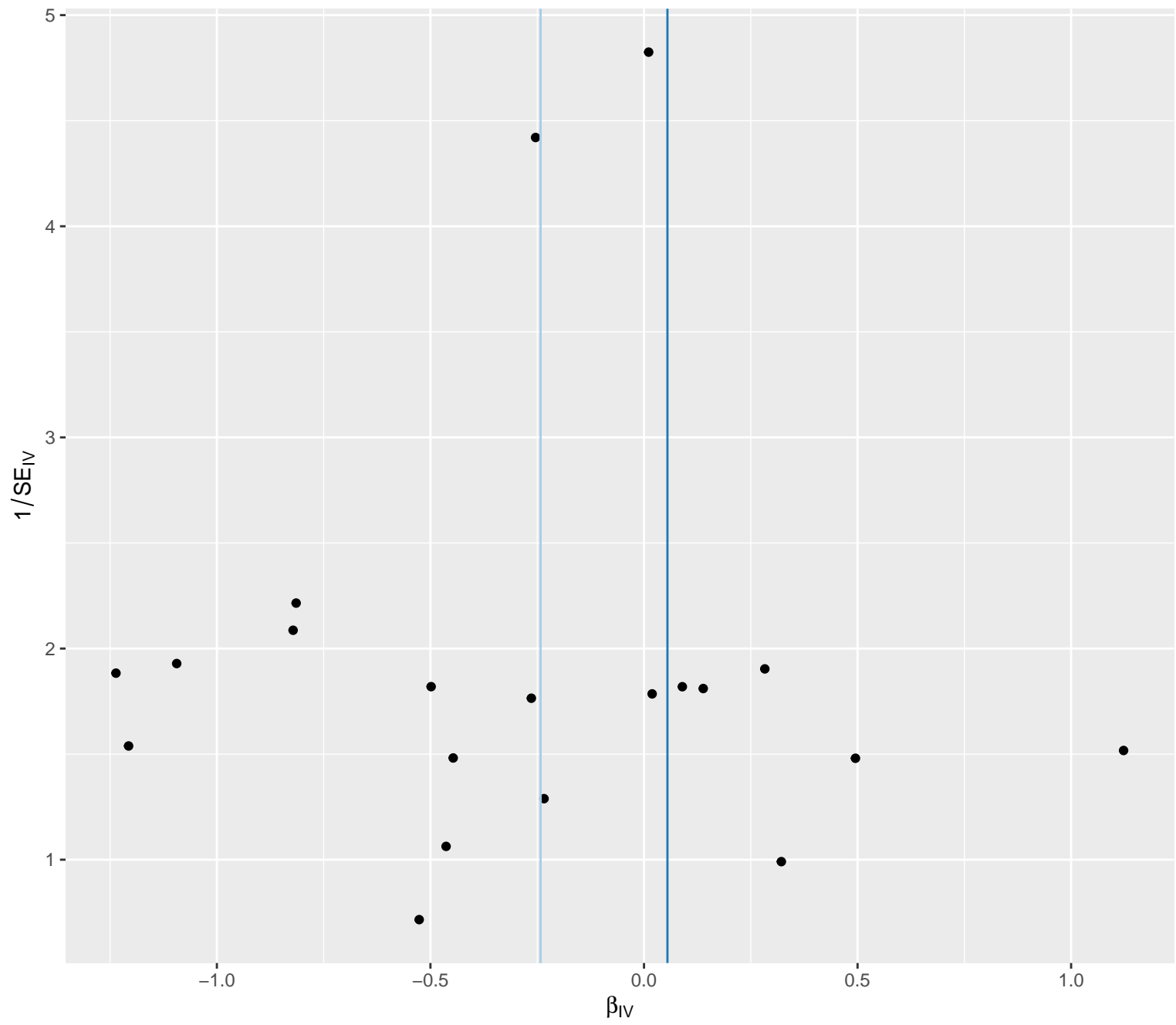

Supplement: Supplementary file 1 [file DataSheet1.zip › supplementary files/S2/GCST90199967/funnelplot.pdf]

# MR Test

- Inverse variance weighted
- MR Egger
- Simple mode
- Weighted median
- Weighted mode

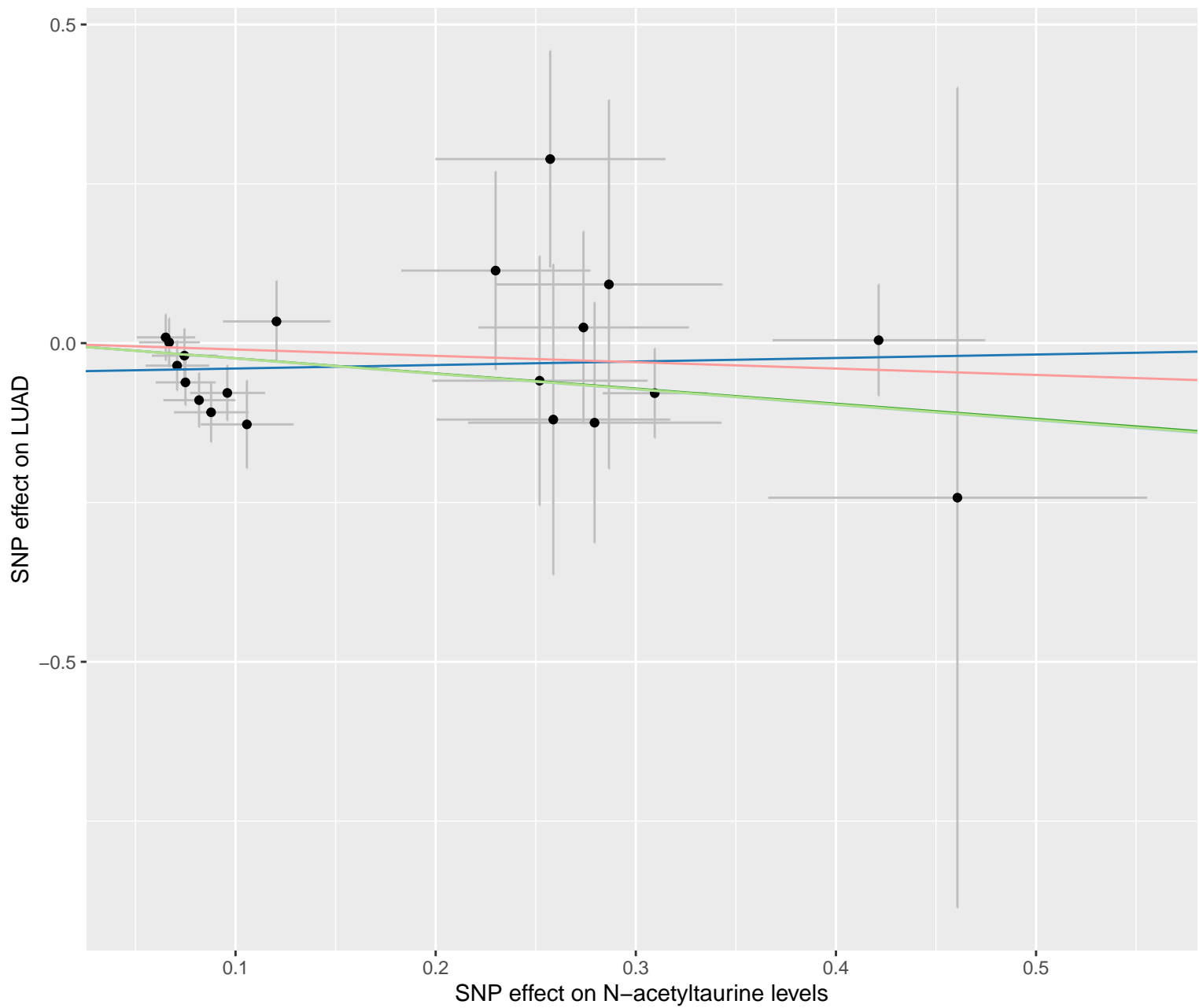

Supplement: Supplementary file 1 [file DataSheet1.zip › supplementary files/S2/GCST90199967/scatter.pdf]

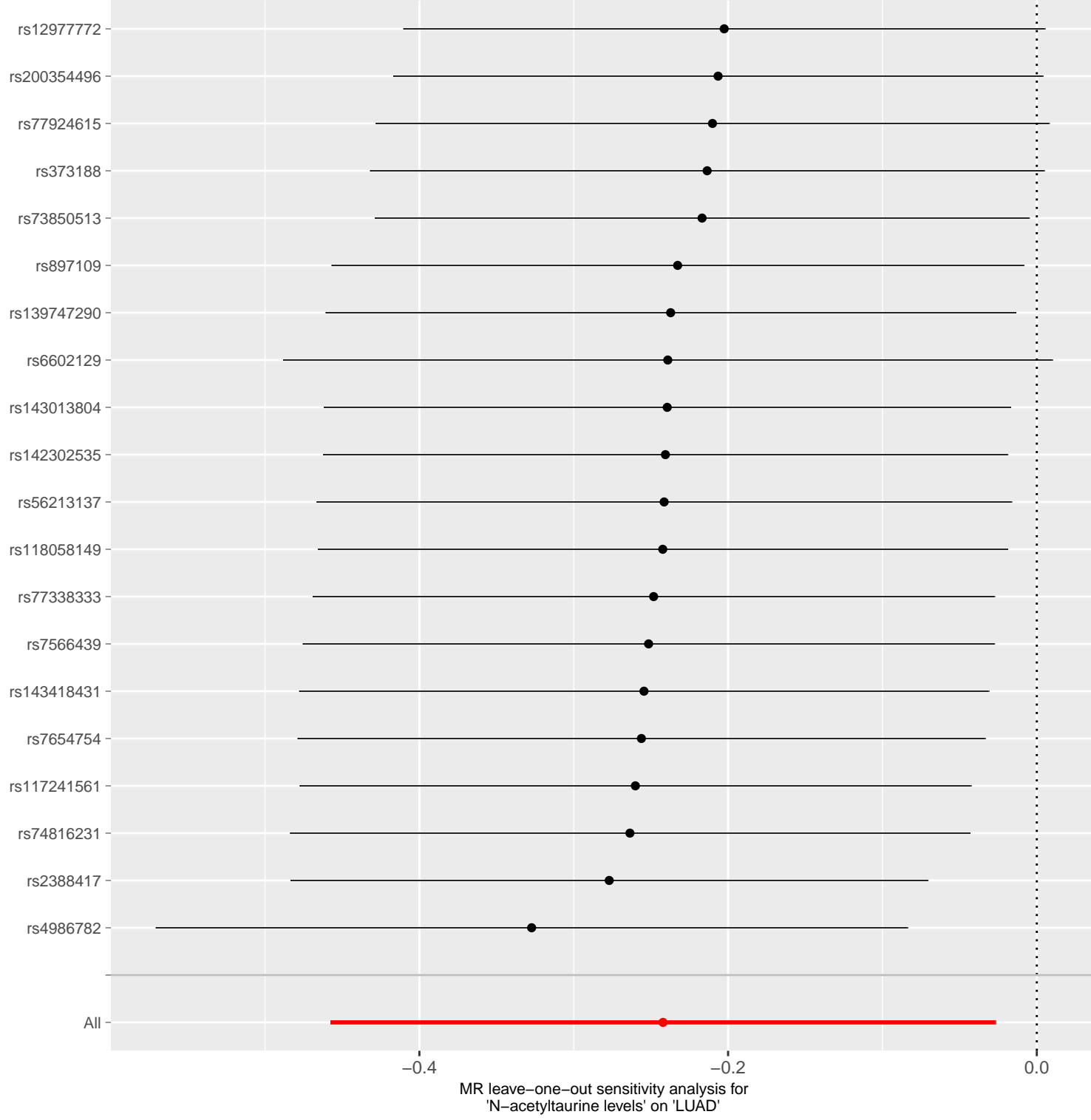

Supplement: Supplementary file 1 [file DataSheet1.zip › supplementary files/S2/GCST90199967/sensitivity-analysis.pdf]

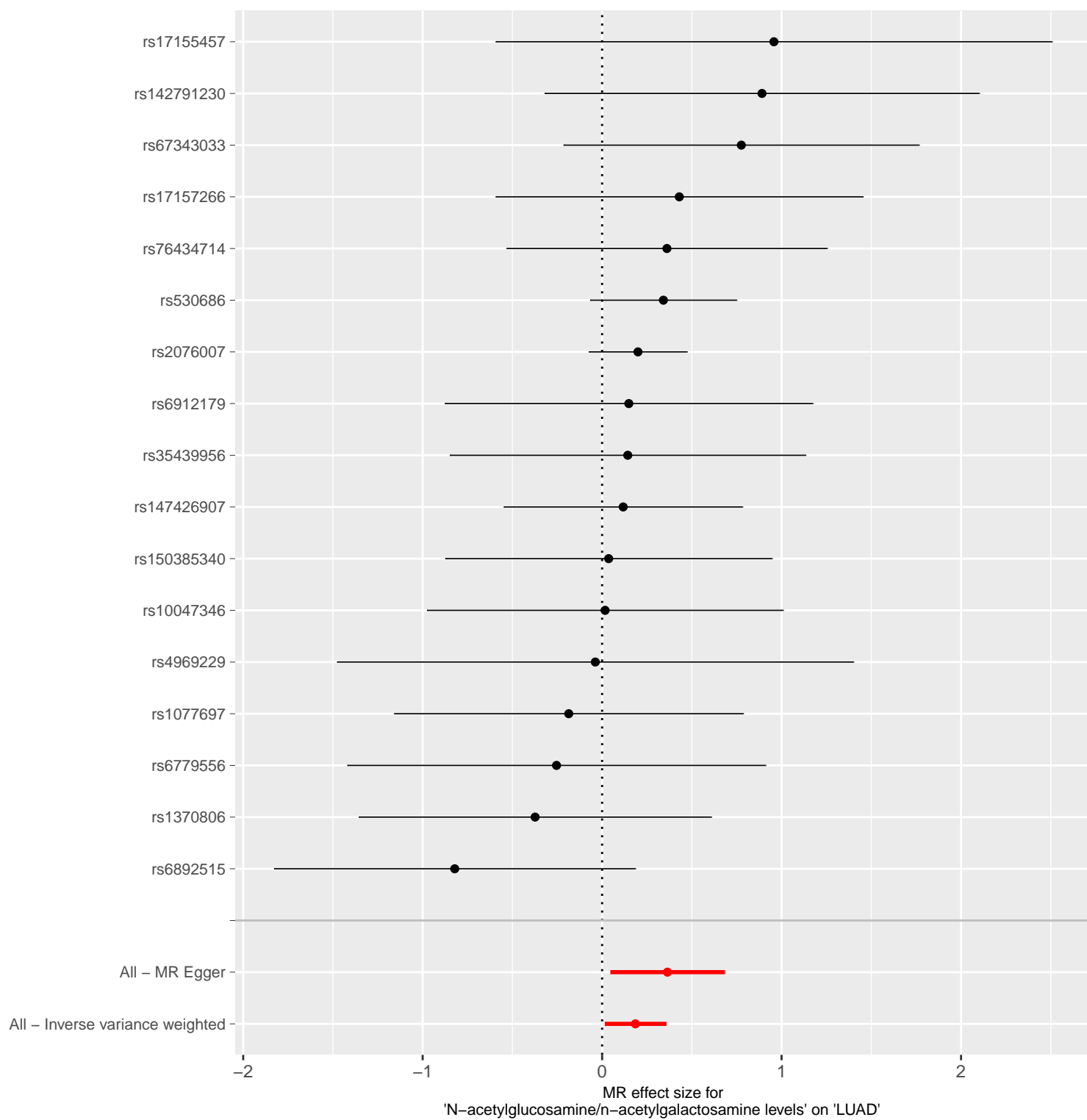

Supplement: Supplementary file 1 [file DataSheet1.zip › supplementary files/S2/GCST90200021/forest.pdf]

# MR Method

- Inverse variance weighted
- MR Egger

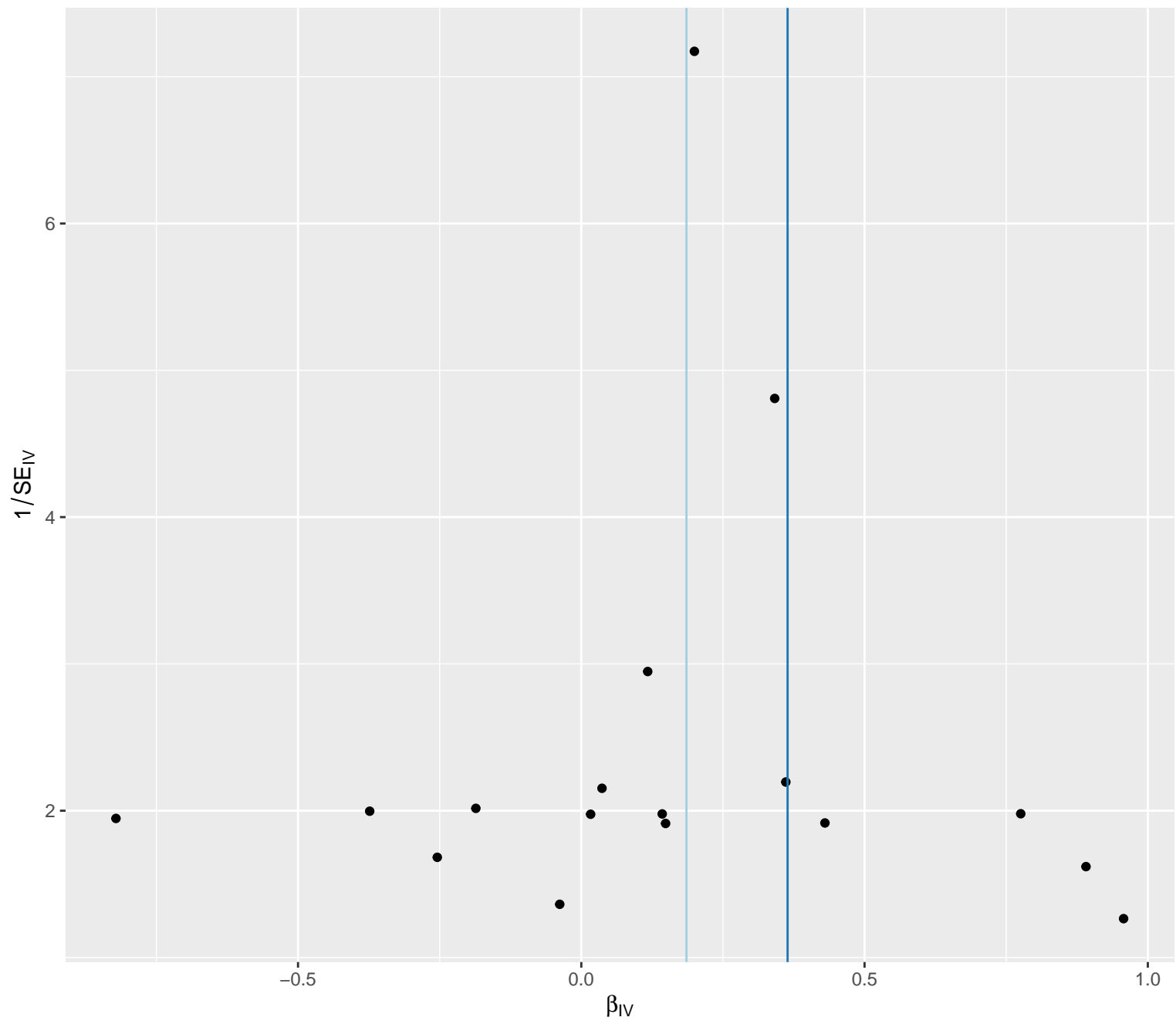

Supplement: Supplementary file 1 [file DataSheet1.zip › supplementary files/S2/GCST90200021/funnelplot.pdf]

# MR Test

- Inverse variance weighted
- MR Egger
- Simple mode
- Weighted median
- Weighted mode

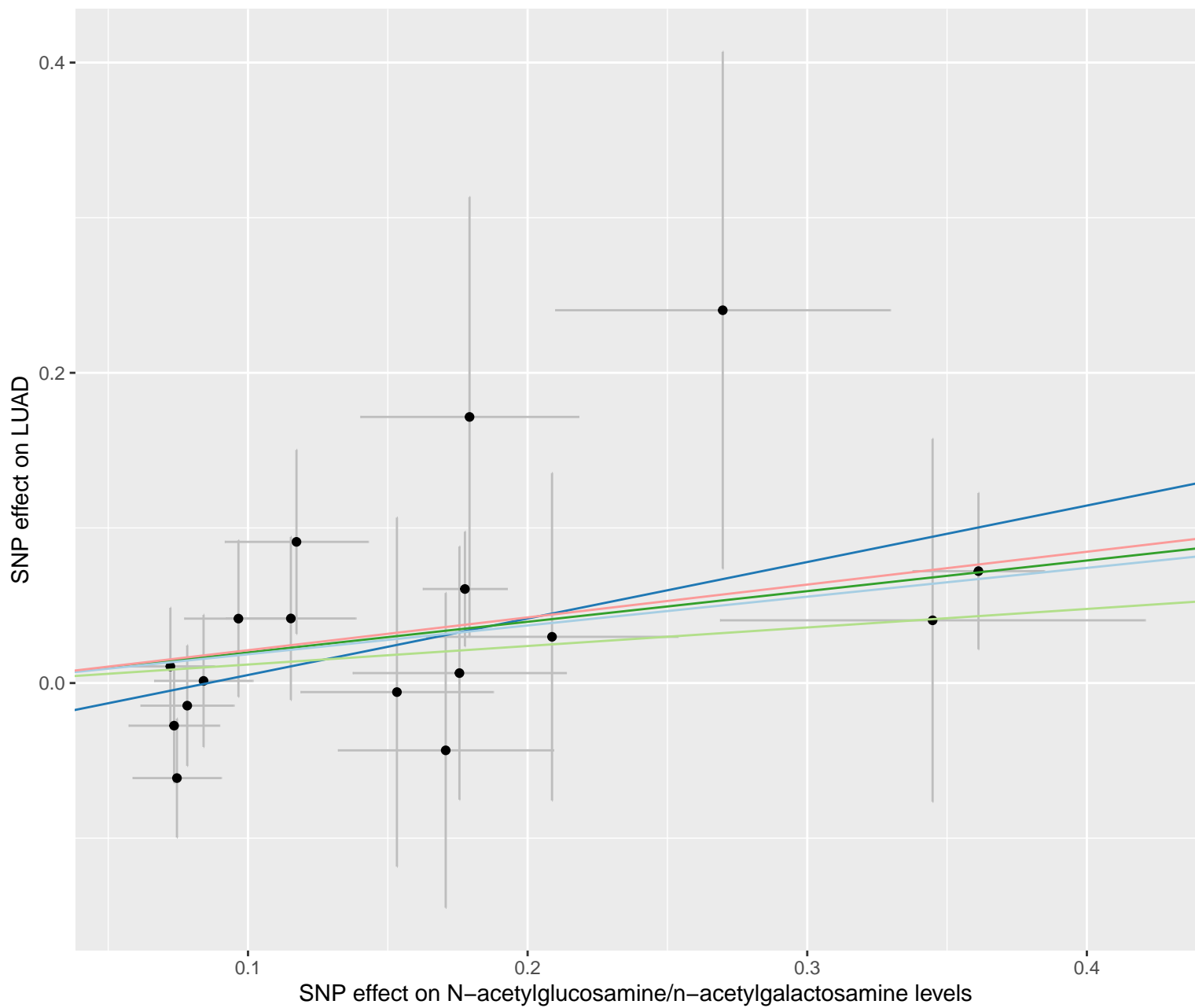

Supplement: Supplementary file 1 [file DataSheet1.zip › supplementary files/S2/GCST90200021/scatter.pdf]

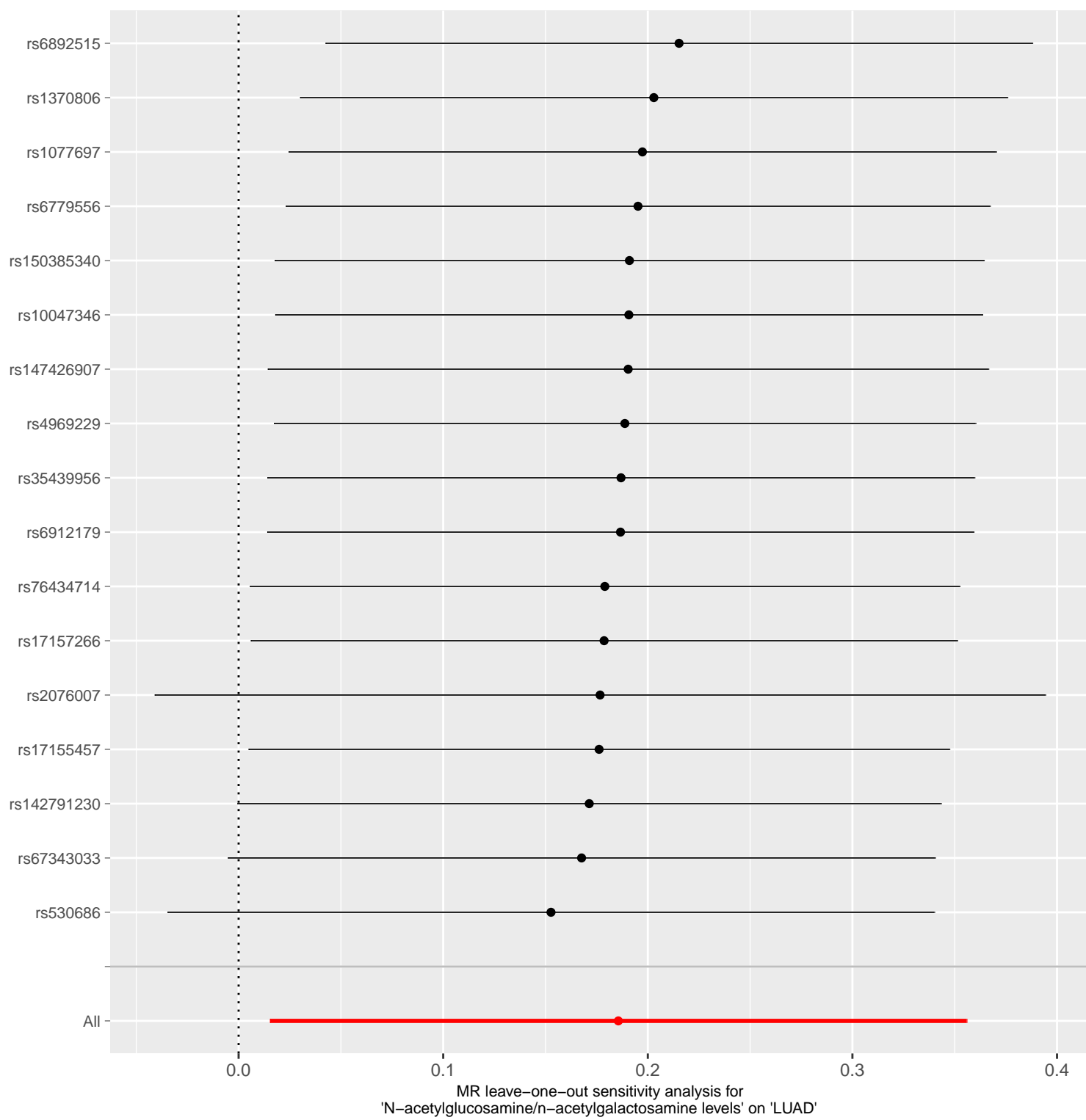

Supplement: Supplementary file 1 [file DataSheet1.zip › supplementary files/S2/GCST90200021/sensitivity-analysis.pdf]

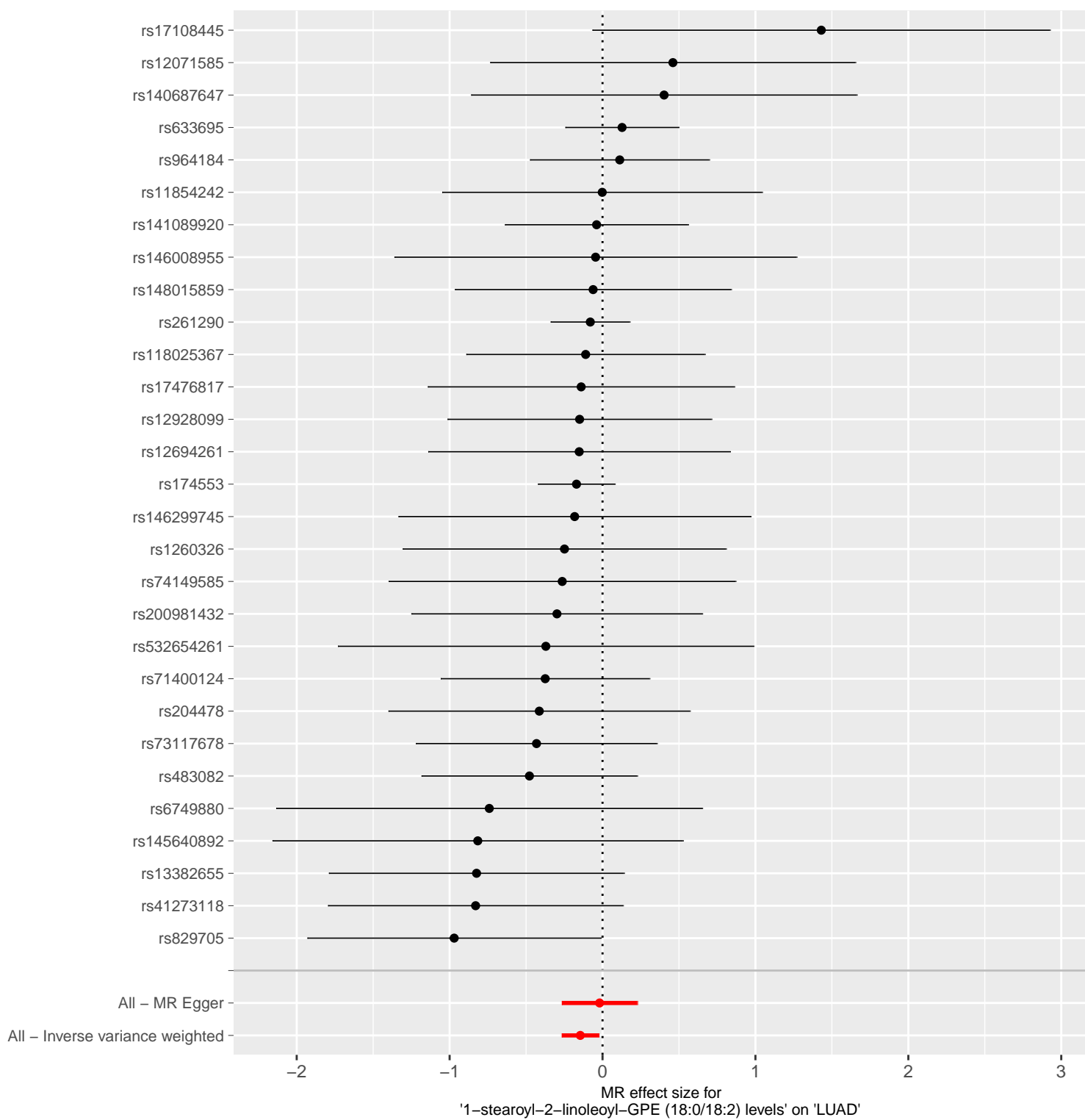

Supplement: Supplementary file 1 [file DataSheet1.zip › supplementary files/S2/GCST90200039/forest.pdf]

# MR Method

- Inverse variance weighted
- MR Egger

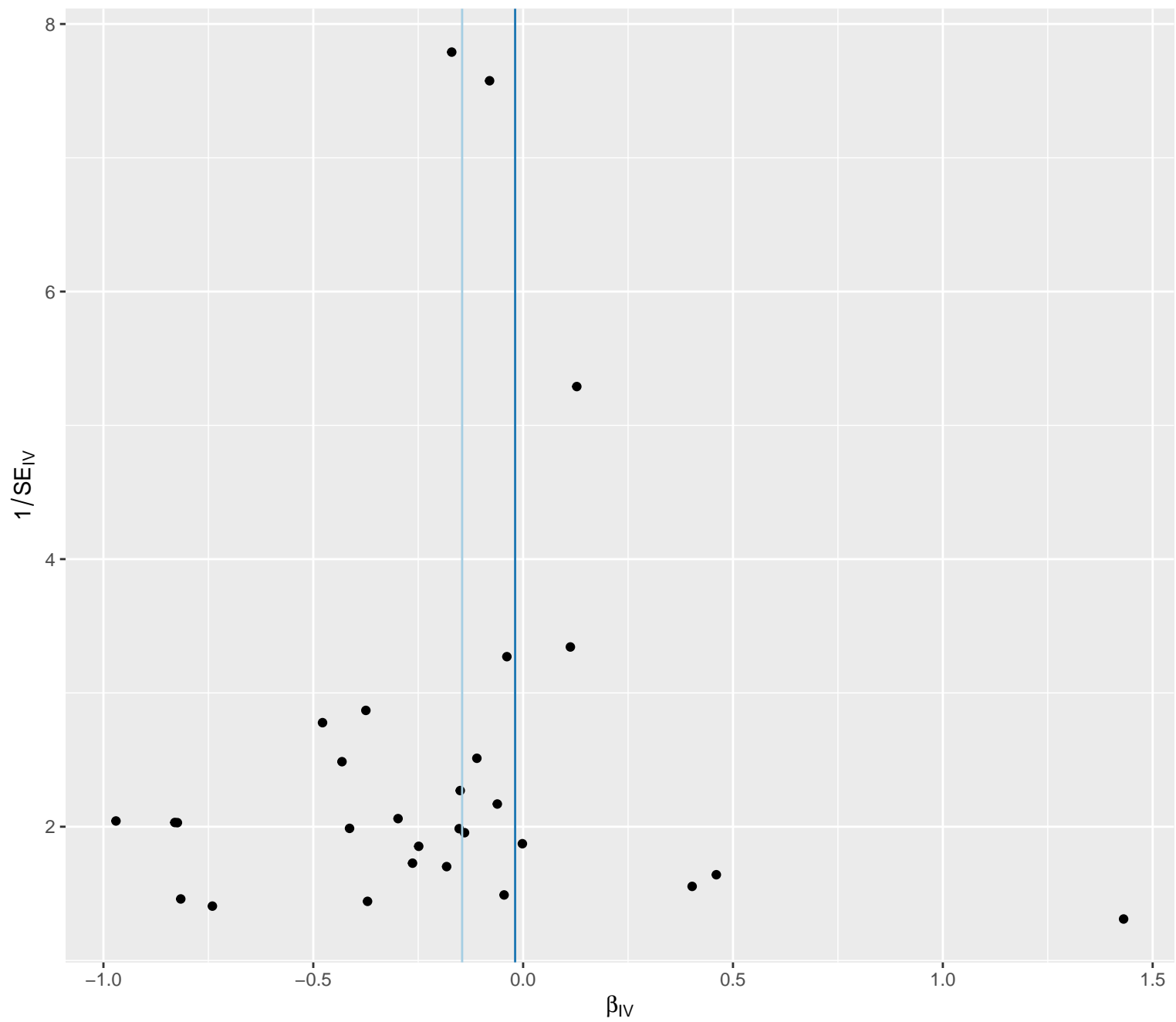

Supplement: Supplementary file 1 [file DataSheet1.zip › supplementary files/S2/GCST90200039/funnelplot.pdf]

# MR Test

- Inverse variance weighted
- MR Egger
- Simple mode
- Weighted median
- Weighted mode

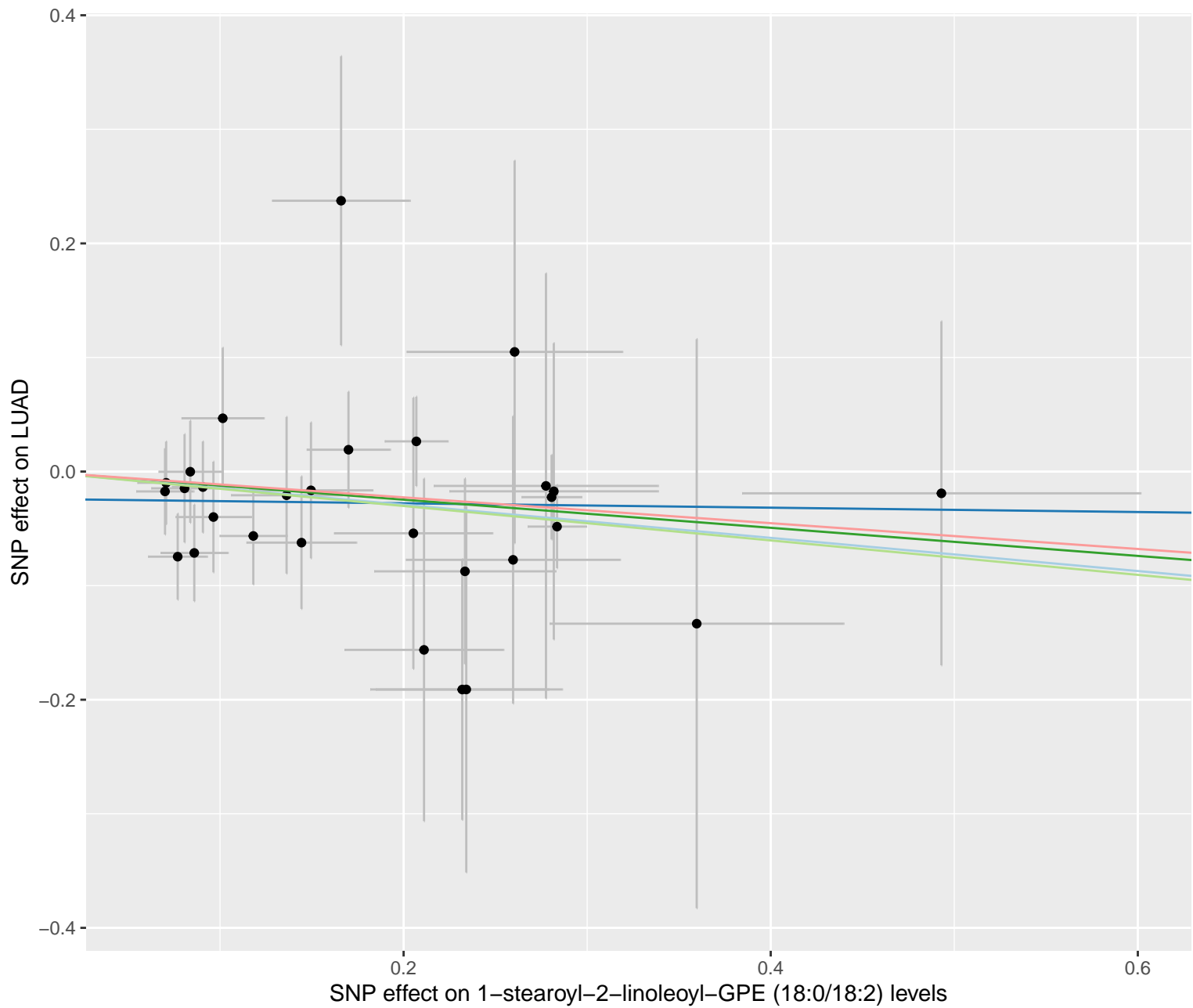

Supplement: Supplementary file 1 [file DataSheet1.zip › supplementary files/S2/GCST90200039/scatter.pdf]

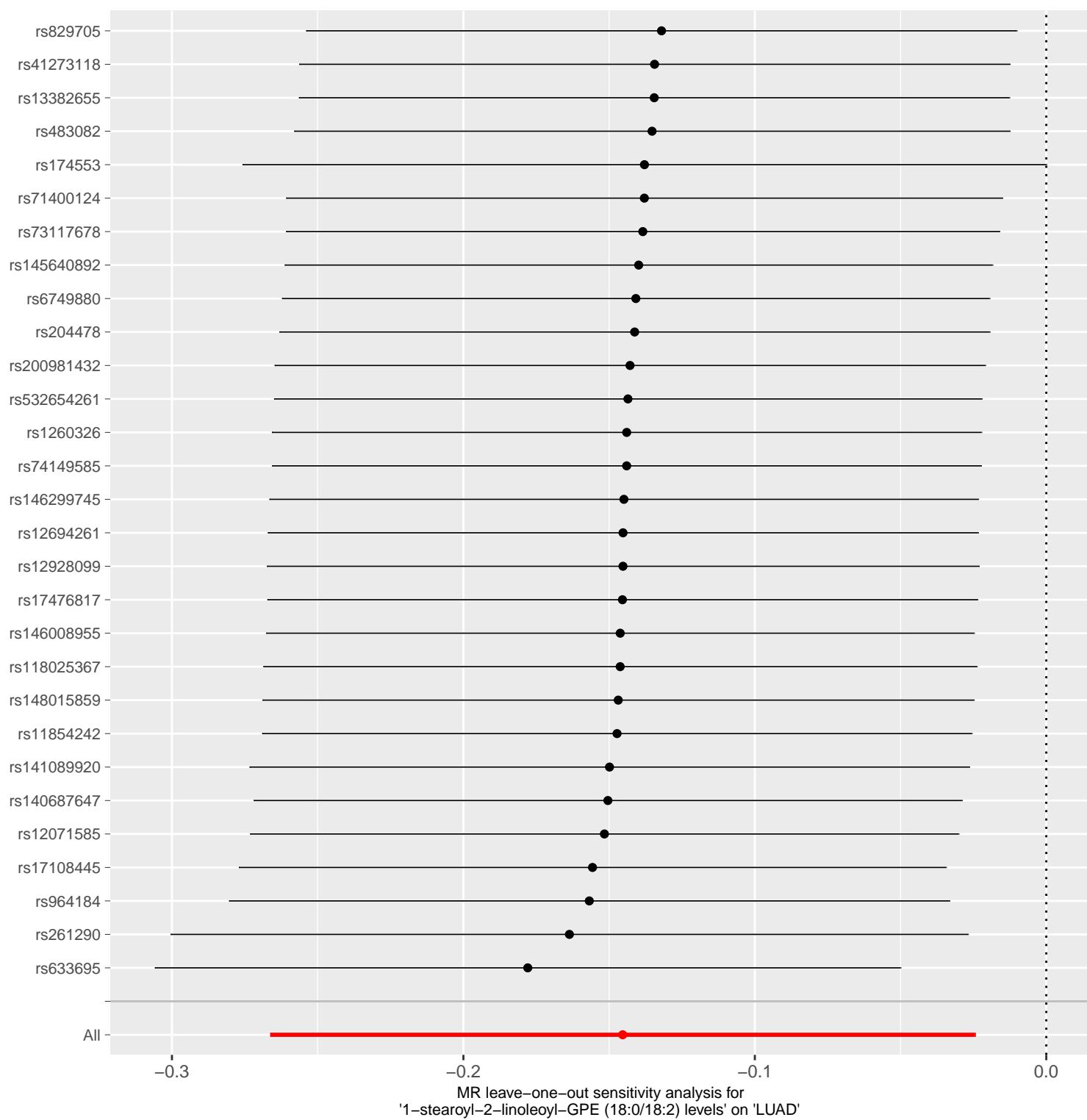

Supplement: Supplementary file 1 [file DataSheet1.zip › supplementary files/S2/GCST90200039/sensitivity-analysis.pdf]

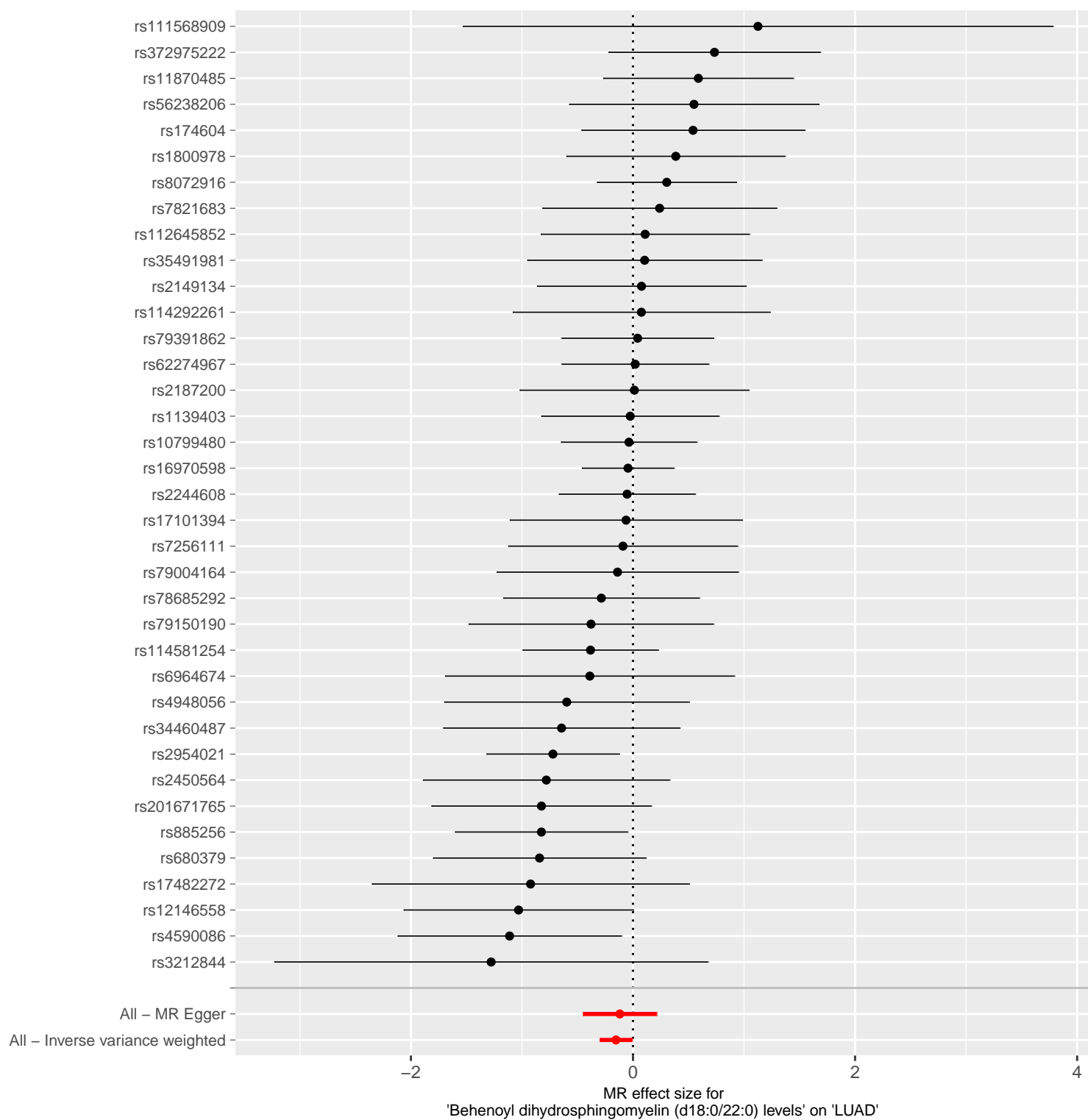

Supplement: Supplementary file 1 [file DataSheet1.zip › supplementary files/S2/GCST90200059/forest.pdf]

# MR Method

- Inverse variance weighted
- MR Egger

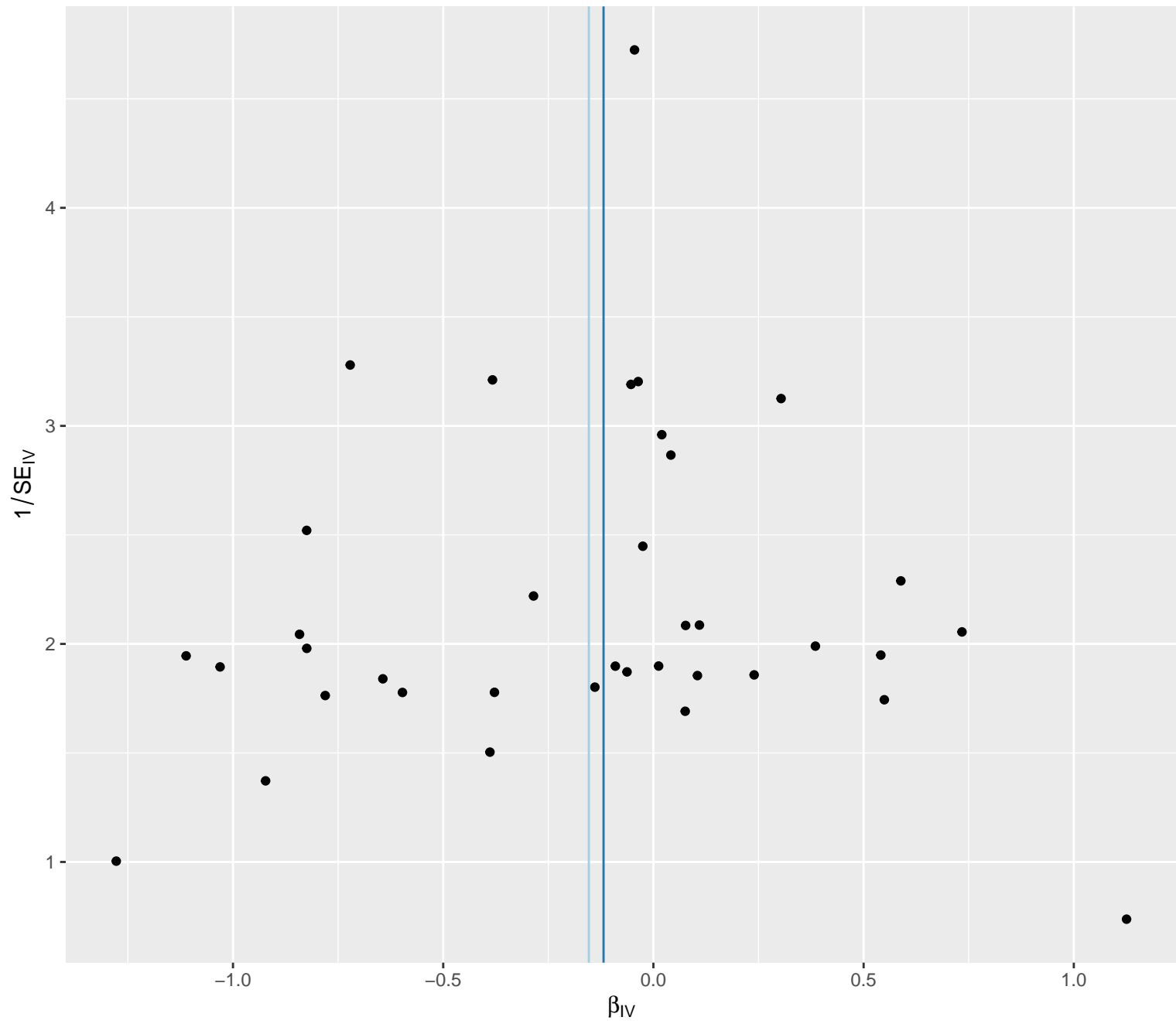

Supplement: Supplementary file 1 [file DataSheet1.zip › supplementary files/S2/GCST90200059/funnelplot.pdf]

# MR Test

- Inverse variance weighted
- MR Egger
- Simple mode
- Weighted median
- Weighted mode

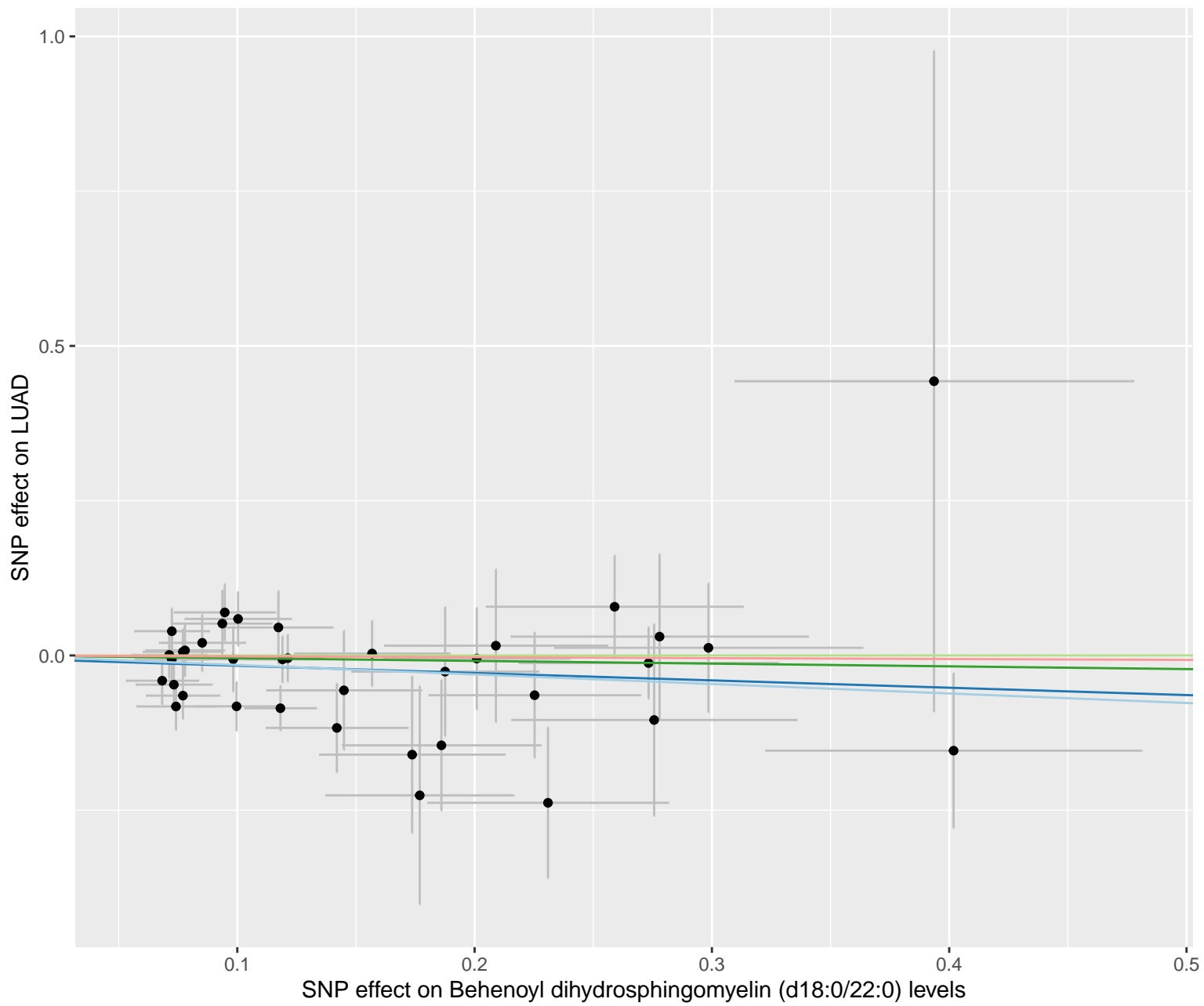

Supplement: Supplementary file 1 [file DataSheet1.zip › supplementary files/S2/GCST90200059/scatter.pdf]

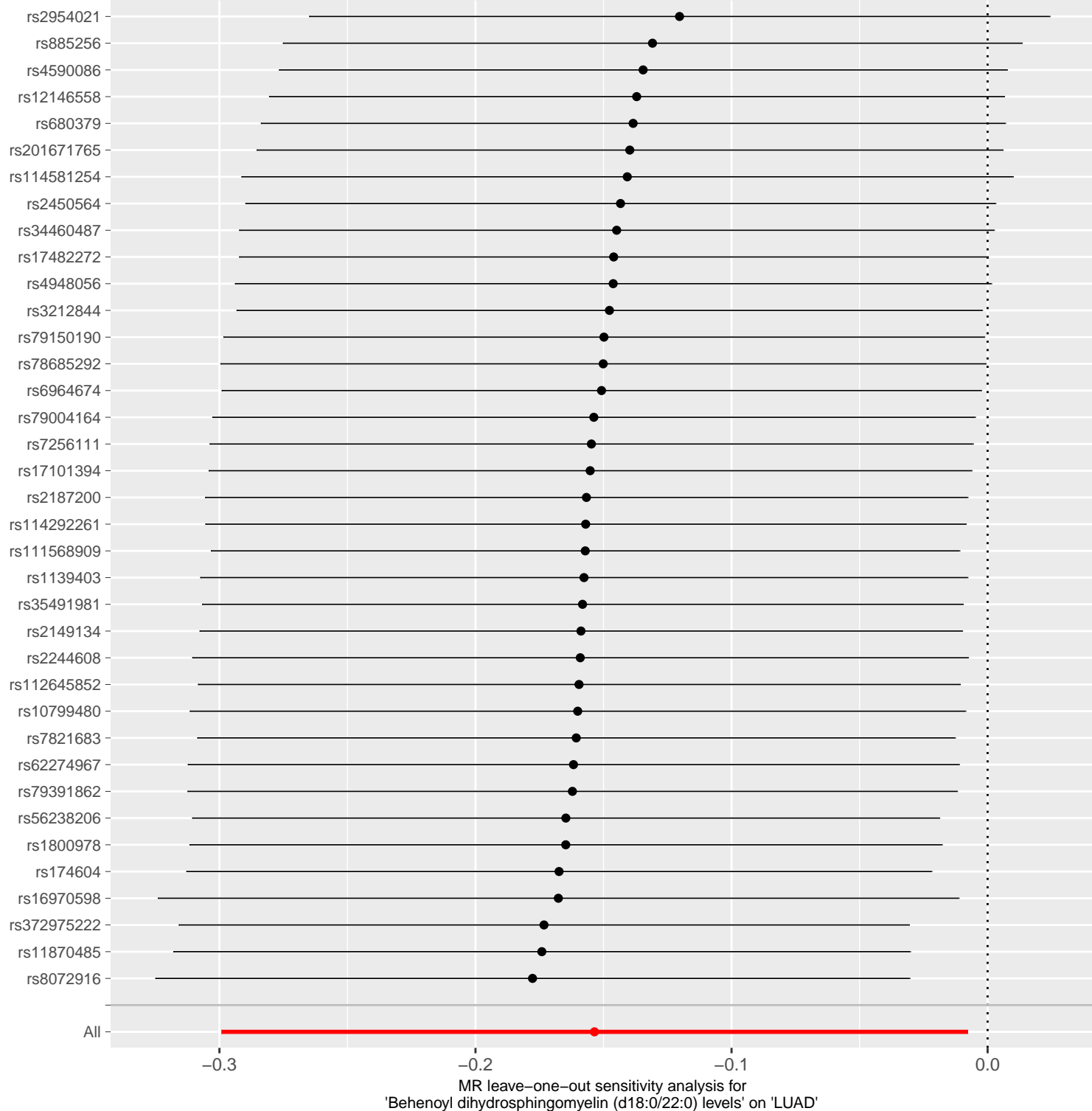

Supplement: Supplementary file 1 [file DataSheet1.zip › supplementary files/S2/GCST90200059/sensitivity-analysis.pdf]

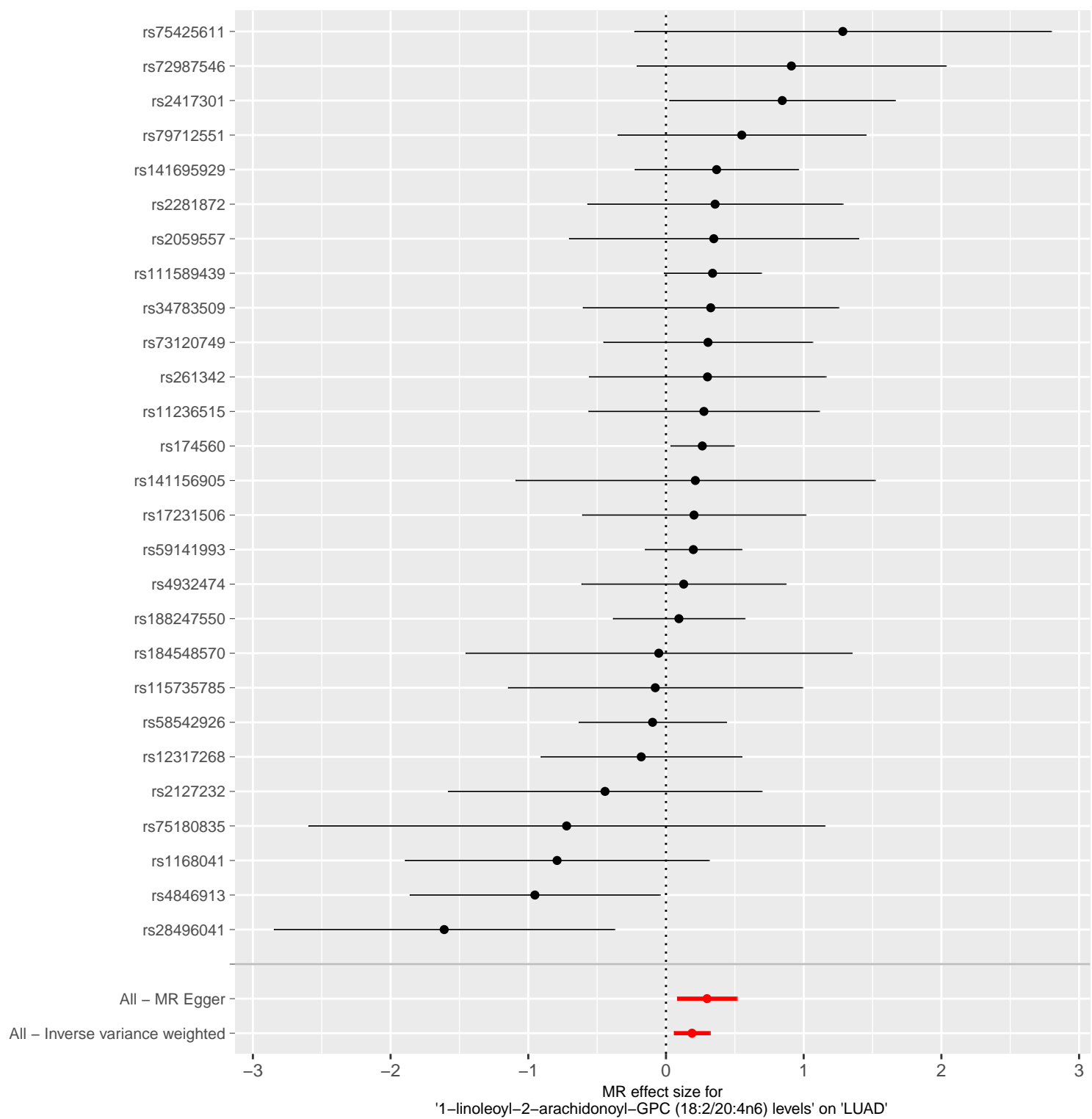

Supplement: Supplementary file 1 [file DataSheet1.zip › supplementary files/S2/GCST90200073/forest.pdf]

# MR Method

- Inverse variance weighted
- MR Egger

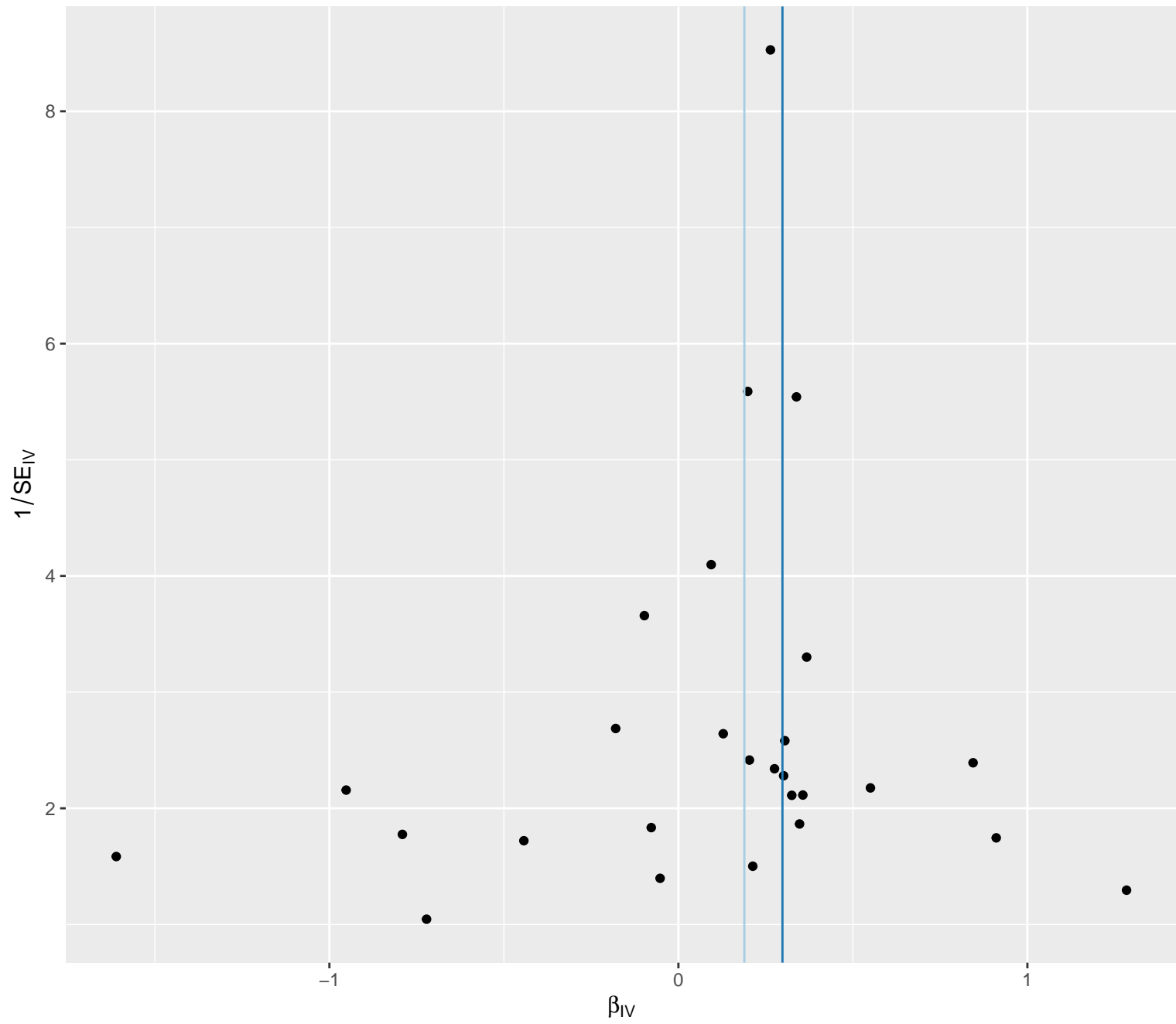

Supplement: Supplementary file 1 [file DataSheet1.zip › supplementary files/S2/GCST90200073/funnelplot.pdf]

# MR Test

- Inverse variance weighted
- MR Egger
- Simple mode
- Weighted median
- Weighted mode

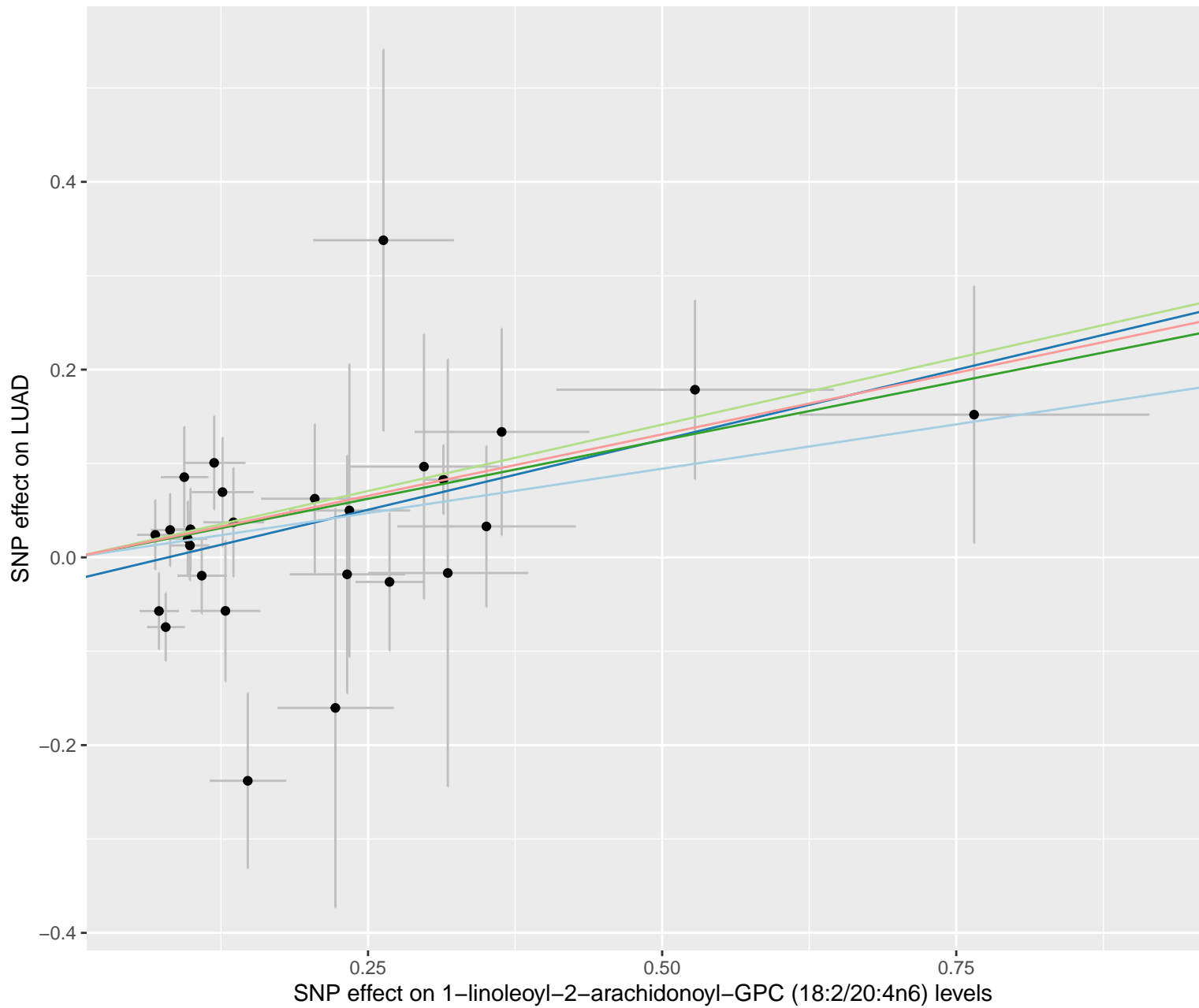

Supplement: Supplementary file 1 [file DataSheet1.zip › supplementary files/S2/GCST90200073/scatter.pdf]

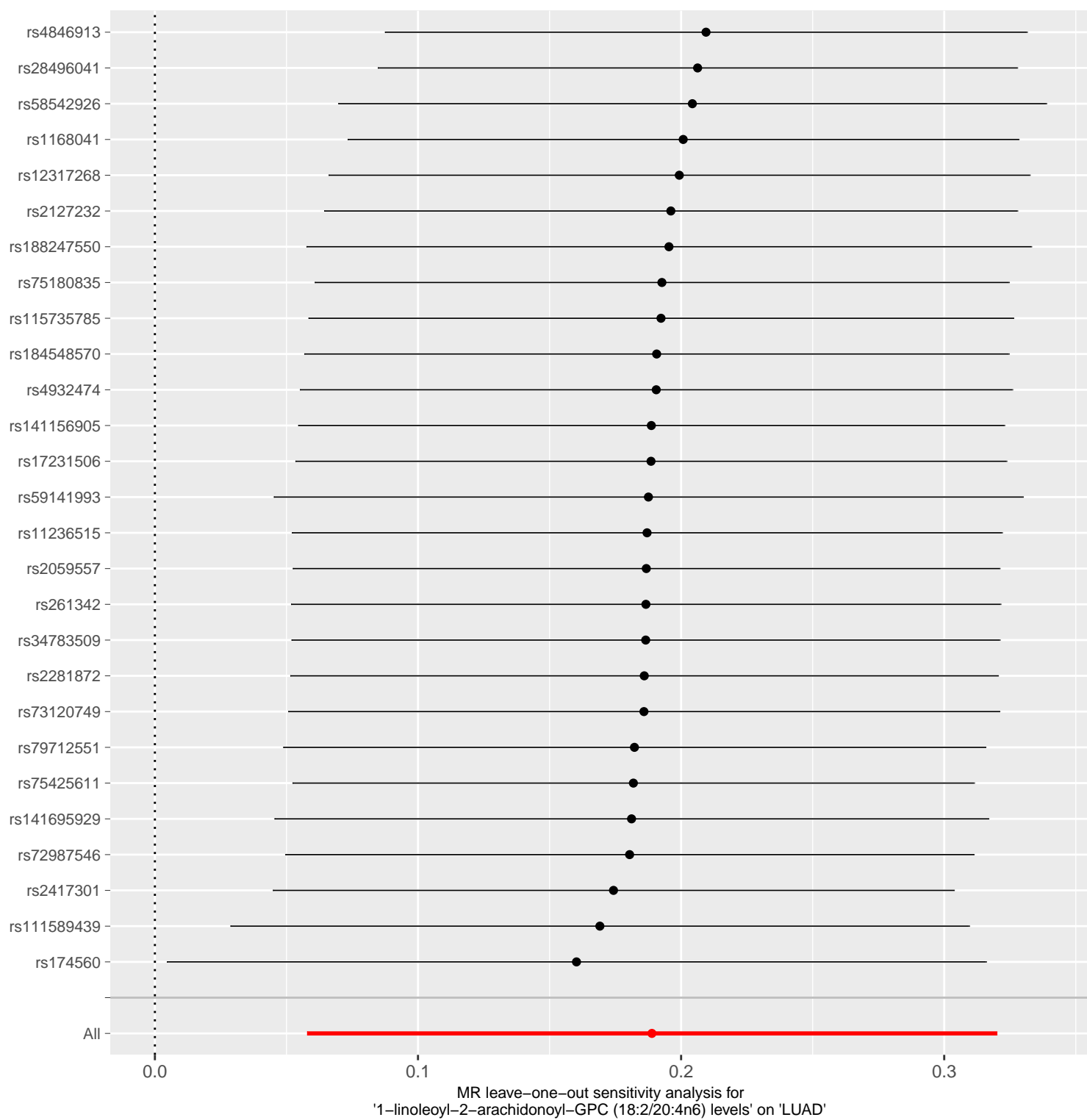

Supplement: Supplementary file 1 [file DataSheet1.zip › supplementary files/S2/GCST90200073/sensitivity-analysis.pdf]
